# Race and Treatment Outcomes in Patients With Metastatic Castration-Sensitive Prostate Cancer: A Secondary Analysis of the SWOG 1216 Phase 3 Trial

**DOI:** 10.1001/jamanetworkopen.2023.26546

**Published:** 2023-08-01

**Authors:** Nicolas Sayegh, Umang Swami, Yeonjung Jo, Georges Gebrael, Benjamin Haaland, Shilpa Gupta, Melissa Plets, Maha H. A. Hussain, David I. Quinn, Primo N. Lara, Ian M. Thompson, Neeraj Agarwal

**Affiliations:** 1 Huntsman Cancer Institute, University of Utah, Salt Lake City; 2 Taussig Cancer Institute, Cleveland Clinic, Cleveland, Ohio; 3 Southwest Oncology Group Statistics and Data Management Center, Seattle, Washington; 4 Northwestern University, Feinberg School of Medicine, Chicago, Illinois; 5 University of Southern California Norris Comprehensive Cancer Center, Los Angeles; 6 University of California Davis Comprehensive Cancer Center, Sacramento; 7 UT Health San Antonio, San Antonio, Texas

## Abstract

**Importance:**

Black patients present with more aggressive disease and experience higher mortality than White patients with prostate cancer. Race and social determinants of health influence prostate cancer-specific mortality and overall survival (OS); however, in a previous trial, Black patients did not have inferior outcomes compared with White patients, possibly because of equitable access to care available in a clinical trial setting.

**Objective:**

To compare differences in survival outcomes of patients with metastatic castration-sensitive prostate cancer (mCSPC) by race in a phase 3 trial with a large proportion of Black patients.

**Design, Setting, and Participants:**

This secondary analysis of patient-level data of a prospective phase 3 randomized clinical trial included patients with newly diagnosed mCSPC enrolled between March 1, 2013, and July 15, 2017. Analysis was conducted between December 2022 and February 2023.

**Interventions:**

Patients receiving androgen deprivation therapy were randomized (1:1) to receive either orteronel 300 mg orally twice daily (experimental group) or bicalutamide 50 mg orally daily (control group).

**Main Outcomes and Measures:**

OS, with progression-free survival (PFS) as a secondary end point.

**Results:**

Among 1313 participants, 135 (10%) identified as Black and 1077 (82%) as White, with an equal racial distribution between groups. Black patients were younger (median [IQR] age, 65.8 [60-70] vs 68.4 [62.5-74.1] years; *P* = .001) and had a higher median (IQR) baseline prostate-specific antigen response rate than White patients (54.7 [19.8-222.0] vs 26.7 [9.2-96.0] ng/mL; *P* < .001). At a median follow-up of 4.9 years, Black and White patients had similar median PFS (2.3 years; 95% CI, 1.8-1.4 years vs 2.9 years; 95% CI, 2.5-3.3 years; *P* = .71) and OS (5.5 years; 95% CI, 4.8-NR vs 6.3 years; 95% CI, 5.7-NR; *P* = .65). The multivariable analysis confirmed similar PFS and OS after adjusting for known prognostic factors. No interaction between race and treatment was observed.

**Conclusions and Relevance:**

In this secondary analysis of a randomized clinical trial studying androgen deprivation therapy with first- or second-generation androgen receptor pathway inhibitors, both Black and White patients demonstrated similar OS and PFS. Equitable access to care may reduce historical differences in outcomes between Black and White patients with advanced prostate cancer.

**Trial Registration:**

ClinicalTrials.gov Identifier: NCT01809691

## Key Points

**Question:**

Do Black patients with metastatic castration-sensitive prostate cancer have worse outcomes than White patients when treated with systemic androgen deprivation therapy combined with a first- or a second-generation androgen receptor pathway inhibitor?

**Findings:**

In this secondary analysis of a randomized phase 3 clinical trial with 1313 patients with metastatic castration-sensitive prostate cancer, there was no statistically significant difference in overall survival and progression-free survival between Black and White patients.

**Meaning:**

These results suggest that providing fair and equal access to health care may reduce the disparities in treatment outcomes between Black and White patients with advanced prostate cancer.

## Teaser

This secondary analysis of a randomized clinical trial evaluates the association of race with survival among patients with metastatic castration-sensitive prostate cancer.

## Introduction

Black patients present with more aggressive disease and experience higher mortality than White patients with prostate cancer.^1^ Race and social determinants of health (SDOH) influence prostate cancer-specific mortality and overall survival (OS). Black patients with prostate cancer have historically been underrepresented in clinical trials, leading to insufficient data on the effectiveness and safety of new treatments for this group.^1^ In a phase II trial involving 100 patients with metastatic castration-resistant prostate cancer (mCRPC), 50 Black patients had similar outcomes receiving abiraterone acetate in the first-line setting compared with White patients.^2^ These hypothesis-generating data suggest that equitable access to care available in a clinical trial setting may negate historically worse outcomes in Black patients with advanced prostate cancer. Herein, we sought to validate these findings using patient-level data from SWOG 1216, a large National Cancer Institute (NCI)–funded randomized phase 3 clinical trial that investigated the effectiveness of orteronel, a nonsteroidal CYP17A1 inhibitor in patients with metastatic castration-sensitive prostate cancer (mCSPC).^3^ To our knowledge, this trial enrolled the highest proportion of Black patients with mCSPC (10.3%) compared with any other phase 3 trial in this setting to date.^1^

## Methods

This is a secondary analysis of a multicenter randomized, open-label, phase 3 study in which patients with mCSPC, enrolled between March 1, 2013, and July 15, 2017, were randomly assigned in a 1:1 ratio to receive either androgen deprivation therapy (ADT) with orteronel 300 mg orally twice daily (experimental group) or ADT with bicalutamide 50 mg orally daily (control group). This secondary analysis only included patients who identified themselves as either Black or White. The NCI’s central institutional review board approved the study. The study was conducted in accordance with the International Conference on Harmonization of Good Clinical Practice guidelines and the principles of the Declaration of Helsinki.^8^ Signed written consent was obtained from all participants. This study followed the Consolidated Standards of Reporting Trials (CONSORT) reporting guideline.

The categorization of race was based on patient self-report. The primary end point was OS, defined as the time from randomization to death from any cause. Secondary end points included progression-free survival (PFS) and prostate-specific antigen (PSA) response rate at 7 months. PFS was defined as the time from randomization to biochemical, radiographic, or clinical progression (per Prostate Cancer Working Group 2 criteria) or death from any cause. PSA responses were categorized as complete response (CR; PSA below 0.2 ng/mL), partial response (PR; PSA between 0.2 and 4.0 ng/mL), and no response (NR; PSA above 4.0 ng/mL [to convert PSA to micrograms per liter, multiply by 1]). A detailed methodology and the primary results of the trial have been previously published (Supplement 1).^3^

### Statistical Analysis

Baseline demographic and clinical characteristics were summarized using descriptive statistics, median (with IQR) for continuous characteristics and count and percentage for categorical characteristics. To test differences in baseline characteristics by race, a χ^2^ test was used for categorical characteristics and a Wilcoxon rank sum test was used for continuous characteristics. The Kaplan-Meier method was used to estimate median PFS and OS and their 95% CIs, and a Cox proportional hazards model was used to perform a univariable and multivariable analysis that adjusted for disease characteristics such as treatment (orteronel vs bicalutamide), disease volume (high vs low), Gleason score (below 8 vs 8 or higher), and PSA. PSA values were log base 2–transformed to normalize a highly skewed distribution. In the multivariable Cox proportional model, we tested for the interaction effect between race and treatment. The analysis was performed using R version 4.2.3 (R Project for Statistical Computing).

## Results

Of 1313 patients enrolled, 135 (10.3%) self-identified as Black and 1077 (82%) as White (Table 1; eFigure 1 in Supplement 2). Treatment assignment, performance status at baseline, and proportion of patients with a Gleason score of 8 or higher were similar between the 2 groups. Extensive disease (per SWOG criteria^3^) was present in 65 Black patients (48.1%) and 524 White patients (48.7%) (*P* = .98). The proportion of patients with visceral metastases was higher among Black patients, with the difference not reaching statistical significance (23 [17.0%] vs 118 [11.0%]; *P* = .05). Black patients were significantly younger at diagnosis of mCSPC (median [IQR] age, 65.8 [60.1-70.4] vs 68.4 [62.5-74.1] years; *P* = .001) and had significantly greater PSA values at baseline (median [IQR], 54.7 [19.8-222.0] vs 26.7 [9.2-96.0] ng/mL; *P* < .001). Bone pain before randomization was present among 34 Black patients (25.2%) and 249 White patients (23.1%), respectively (*P* = .61).

**Table 1.** Baseline Characteristics and PSA Responses

Variable	Patients, No. (%)		*P* value
	Black (n = 135)	White (n = 1077)	
Age, median (IQR), y	65.8 (60.1-70.4)	68.4 (62.5-74.1)	.001
Zubrod performance status			
Fully active	89 (65.9)	726 (67.5)	.68
Restricted activity	39 (28.9)	313 (29.1)	
No work, ambulatory	6 (4.4)	33 (3.1)	
Limited self-care	1 (0.7)	3 (0.3)	
Gleason score ≥8	76 (61.3)	632 (62.1)	.93
High volume disease^a^	65 (48.1)	524 (48.7)	.98
Visceral metastases	23 (17.0)	118 (11.0)	
Log 2 PSA at baseline, median (IQR), ng/mL	5.77 (4.31-7.79)	4.74 (3.19-6.59)	<.001
Bone pain	34 (25.2)	249 (23.1)	
Treatment group			
Bicalutamide	71 (52.6)	538 (50)	.63
Orteronel	64 (47.4)	539 (50)	
PSA response at 7 mo			
Complete response	63 (55.8)	558 (62.8)	.30
Confirmed partial response	34 (30.1)	236 (26.6)	
No response	16 (14.2)	94 (10.6)	

Abbreviation: PSA, prostate-specific antigen.

^a^ Defined as greater than minimal involvement of vertebrae, pelvic bones, and/or lymph nodes.

At a median follow-up of 4.9 years, the median OS for Black patients was 5.5 years (95% CI, 4.8 to not reached), compared with 6.3 years (95% CI, 5.7 to not reached) for White patients (Figure, A). However, the difference was not statistically significant on univariable analysis (hazard ratio, 0.94; 95% CI, 0.71-1.24; *P* = .65). Similarly, the median PFS was 2.3 years (95% CI, 1.8-4.1 years) for Black patients and 2.9 years (95% CI, 2.5-3.3 years) for White patients overall (HR, 0.96; 95% CI, 0.76-1.20; *P* = .71) (Figure, B). There was no observed interaction between race and treatment (*P* value for interaction: PFS, *P* = .77 and OS, *P* = .91) (eFigure 2 in Supplement 2). After adjusting for the treatment group (orteronel vs bicalutamide), extent of disease, Gleason score, age, and baseline PSA, PFS, and OS rates remained comparable in both Black and White patients (Table 2).

**Figure.**
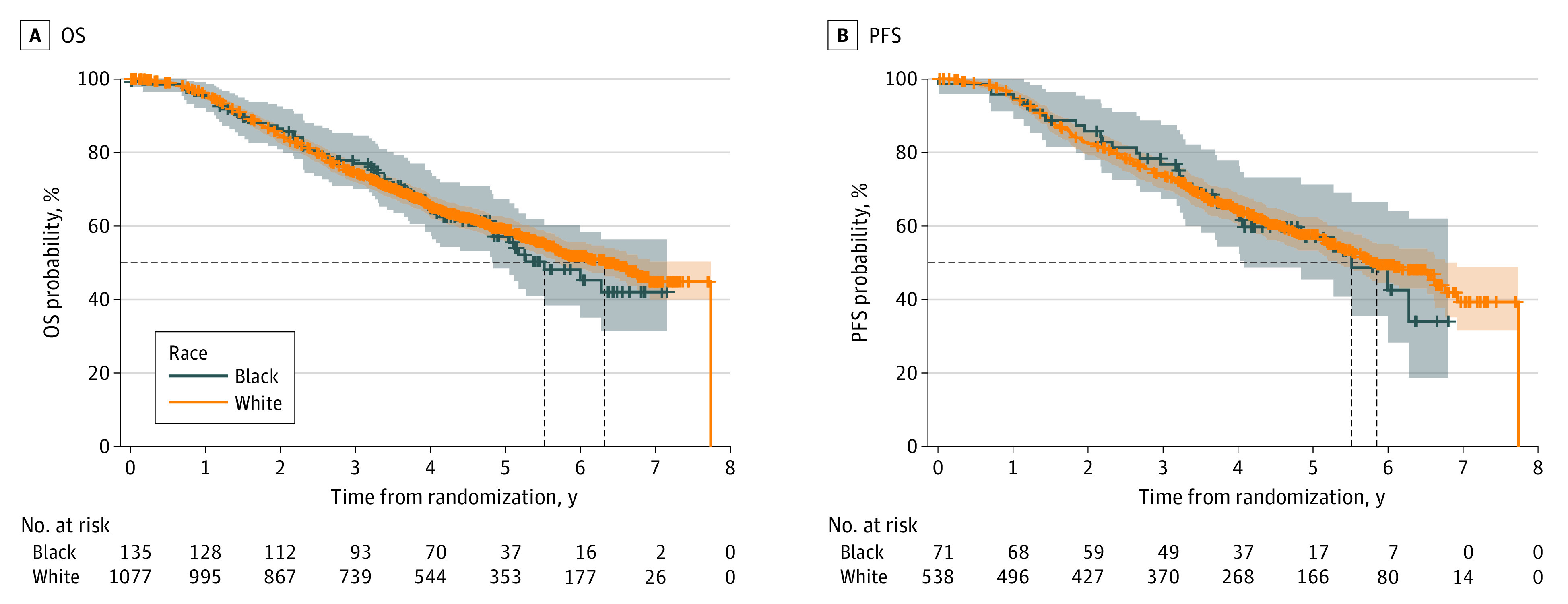
Kaplan-Meier Estimates of Overall Survival (OS) and Progression-Free Survival (PFS) by Race in the Overall Population Dashed lines indicate median time participants experienced OS (panel A) or PFS (panel B).

**Table 2.** Multivariable Analysis of Progression-Free Survival (PFS) and Overall Survival (OS)

Characteristic	PFS, HR (95% CI)	*P* value	OS, HR (95% CI)	*P* value	
Race					
White	1 [Reference]	NA	1 [Reference]	NA	
Black	1.13 (0.88-1.43)	.30	0.99 (0.74-1.33)	>.99	
Black (univariable)	0.96 (0.76-1.2)	.71	0.94 (0.71-1.24)	.65	
Treatment					
Bicalutamide	1 [Reference]	NA	1 [Reference]	NA	
Orteronel	0.58 (0.5-0.67)	<.001	0.87 (0.72-1.05)	.14	
High volume disease (vs low volume disease)^a^	1.92 (1.65-2.24)	<.001	2.03 (1.67-2.48)	<.001	
Gleason score ≥8	1.23 (1.05-1.44)	.01	1.20 (0.98-1.46)	.07	
Log 2 PSA	1.14 (1.10-1.18)	<.001	1.09 (1.05-1.14)	<.001	
Age (per 1 y)	1.00 (0.99-1.00)	.30	1.01 (1.00-1.02)		.30

Abbreviations: NA, not applicable; PSA, prostate-specific antigen.

^a^ Defined as greater than minimal involvement of vertebrae, pelvic bones, and/or lymph nodes.

Among Black patients, 63 of 135 (55.8%) had a complete PSA response at 7 months and 16 (14.2%) had no PSA response at 7 months, compared with 558 (62.8%) and 94 (10.6%) of 1077 White patients, respectively. However, the difference was not statistically significant (*P* = .30) (Table 1).

## Discussion

This secondary analysis of the SWOG 1216 trial found that ADT plus bicalutamide or ADT plus a novel androgen receptor pathway inhibitor (ARPI) elicited similar PFS, PSA response, and OS in Black and White patients with mCSPC, despite Black patients presenting with more aggressive disease features (younger age and higher baseline PSA level). In a race-stratified trial enrolling 100 patients with mCRPC (50 Black and 50 White patients), patients were treated with abiraterone acetate and prednisone as first-line therapy in addition to ongoing ADT.^2^ The median radiographic PFS and OS were similar at 16.6 vs 16.8 months and 35.9 vs 35.7 months in Black and White patients, respectively. Additionally, a retrospective study analyzed the clinical outcomes of 3808 patients treated with second-generation ARPIs in the first-line mCRPC setting.^4^ With the caveat of a short median follow-up of 13 months, the study did not show worse outcomes for Black patients compared with White patients. Another retrospective study of 168 patients^5^ (of whom 92 were Black) with mCSPC treated with intensified ADT did not show any difference in survival outcomes between Black and White patients with either abiraterone or docetaxel. To summarize, these hypothesis-generating data from a phase 2 trial and multiple retrospective clinical studies showed similar survival outcomes in Black and White patients treated similarly.

Historically, registration phase 3 clinical trials for metastatic prostate cancer have accrued disproportionately low numbers of Black patients, and this trend has worsened in the last 3 decades, especially in industry-sponsored trials.^6^ The SWOG 1216 trial was a large phase 3, federally funded trial in the mCSPC setting that enrolled a proportionate number of Black patients.

Our findings from the patient-level data from a prospective phase 3 trial validate the previous population-based research findings indicating that Black patients with advanced prostate cancer achieve comparable outcomes when health care access is equalized and treatment is standardized. A 2023 meta-analysis of more than 1 million patients with prostate cancer demonstrated that race was associated with prostate cancer-specific mortality and OS.^7^ When SDOH such as age, comorbidities, insurance status, income status, extent of disease, geography, standardized treatment, and equitable and harmonized insurance benefits were accounted for, there was no difference in prostate cancer-specific mortality or OS between Black and White patients across all disease stages.

### Limitations

This study had several limitations. In the SWOG 1216 trial, orteronel improved PFS but not OS, and therefore is not being used in the current practice, which may have limited the generalizability of this study in the clinical setting compared with other ARPIs. Also, while to our knowledge this trial enrolled the largest proportion of Black patients in the mCSPC setting to date, the sample size of the primary trial may not be sufficient for the subgroup analyses of Black patients or to detect small effect sizes. This can limit the statistical power of the secondary analysis. We acknowledge that this is an exploratory analysis in nature and should be interpreted with caution.

## Conclusions

In this secondary analysis of a large randomized, multicenter phase 3 clinical trial, we showed using high-quality patient-level data that Black patients had similar survival outcomes to White patients with mCSPC. These results support the hypothesis that equitable access to care as available in a clinical trial setting negates disparities in outcomes previously associated with Black patient populations. Possible avenues for action include accounting for variables linked to SDOH in research and tackling modifiable cultural, economic, and geographic factors in clinical settings.^1^

## Notes

Supplement 1.
Trial Protocol and Statistical Analysis Plan

Supplement 2. **eFigure 1.** CONSORT Diagram for the S1216 Trial **eFigure 2.** Kaplan-Meier Estimates of Overall Survival and Progression-Free Survival by Race

Supplement 3.
Data Sharing Statement
