# Supplementary material for: Race and Treatment Outcomes in Patients With Metastatic Castration-Sensitive Prostate Cancer: A Secondary Analysis of the SWOG 1216 Phase 3 Trial
Source: JAMA Netw Open. 2023 Aug 1;6(8):e2326546. doi: 10.1001/jamanetworkopen.2023.26546 (PMC10394570; doi:10.1001/jamanetworkopen.2023.26546)
Supplement: Supplement 1. — Trial Protocol and Statistical Analysis Plan [file jamanetwopen-e2326546-s001.pdf]

SWOG
Leading cancer research. Together.

GROUP CHAIR'S OFFICE
Charles D. Blanke, MD
CHAIR
3181 SW Sam Jackson Pk Rd
MC: L586
Portland, OR 97239
503-494-5586
503-346-8038 FAX

OPERATIONS OFFICE
4201 Medical Dr
Suite 250
San Antonio, TX 78229
210-614-8808
210-614-0006 FAX

STATISTICAL CENTER
1730 Minor Ave
Suite 1900
Seattle, WA 98101
206-652-2267
206-342-1616 FAX

1100 Fairview Ave North
M3-C102
PO Box 19024
Seattle, WA 98109
206-667-4623
206-667-4408 FAX

swog.org

Distribution Date: TBD
Version Date: April 7, 2017

TO: ALL NATIONAL CLINICAL TRIALS NETWORK (NCTN) MEMBERS – U.S. INSTITUTIONS ONLY

FROM: Nicki Trevino, Protocol Coordinator (E-mail: ntrevino@swog.org

RE: **S1216**, "A Phase III Randomized Trial Comparing Androgen Deprivation Therapy + TAK-700 With Androgen Deprivation Therapy + Bicalutamide in Patients With Newly Diagnosed Metastatic Hormone Sensitive Prostate Cancer." Study Chairs: Drs. N. Agarwal, M. Hussain, P. Lara, T. Crispino.

REVISION #5

Study Chair: Neeraj Agarwal, M.D.
Phone number: 801/587-4765
E-mail: neeraj.agarwal@hci.utah.edu

IRB Review Requirements
( ) Full board review required
(√) Expedited review allowed
( ) No review required

Status Change
( ) IRB Review only
( ) Activation
( ) Closure
( ) Reactivation

Protocol changes
( ) Eligibility changes
(√) Treatment / Dose Modification / Study Calendar changes
(√) Informed Consent changes
- (√) Patient notification not required
- ( ) Patient notification required

( ) Scientific / Statistical Consideration changes
( ) Specimen Submission changes
( ) Data Submission / Forms changes
(√) Editorial / Administrative changes
( ) Other:

Sites using the CIRB as their IRB of record: The protocol and/or informed consent form changes have been approved by the CIRB and must be activated within 30 days of the CIRB posting of this notice. If local approval is not granted within 30 days, accrual must be suspended until approval is obtained.

Sites not using the NCI CIRB: Per CTMB Guidelines, the protocol updates and/or informed consent changes must be approved by local IRBs within 90 days of distribution of this notice. The changes in this revision are effective upon approval by the local IRB; however, any changes to eligibility become effective 6 weeks after distribution of this notice. If local approval is not granted within 6 weeks, accrual must be suspended until approval is obtained.

REVISION #5

The above-referenced protocol has been updated as follows:

1. The version date has been updated.

2. Page 6: The CTSU Address and Contact Information has been updated.

3. Section 5.1.b: The eligibility information for late induction patients has been reworded for clarity.

4. Section 5.3 g: The timeframe for measuring blood pressure has changed from “within 14 days” to “within 28 days” prior to registration.

5. Section 7.0 & Section 8.6: Contact information for Dr. Shilpa Gupta has been inserted.

6. Section 8.4.b2: The last sentence with the instruction about when bicalutamide should be permanently discontinued, has been revised from “≥ 60 days” to “> 60 days” for consistency and accuracy throughout the protocol.

7. Section 9.0, Study Calendar: ‘CT or MRI of Abdomen & Pelvis’ and ‘Bone Scan or PET scan’ in the ‘FU After Prog’ column have been removed. The corresponding ‘≠’ footnote has been updated to indicate that disease assessment by these methods ends at progression.

8. Section 9.0: Calendar footnotes have been updated as follows:
   (π) “PSA” has been deleted. “PSA should be assessed every 6 months for the first 2 years and annually until 10 years after registration or until death” has been added.

   (λ) “cortisone” has been replaced with “cortisol” and “Draw prior to starting TAK-700” has been added.

   (√) “If patient had serum drawn at baseline before September 23, 2014 continue with serum collection at designated time points” has been added.

   (ϖ) “For patients with a history of radical prostatectomy” has been replaced with “Submit 30 slides from radical prostatectomy or 10-15 slides from biopsy (see Section 15.2 a).

9. Section 9.0: Allowable windows have been added underneath the footnotes.

10. Section 10.2.2: This section has been reworded for clarity.

11. Section 18.5: The appendix item “Specimen Supplement Sheet” has been added to the protocol. This was previously a separate document and is now being included in the protocol document.

The Model Consent Form has been revised as follows:

1. The Version Date has been updated.

2. Pages 9-10: As a result of the most recent Data and Safety Monitoring Committee review, the following changes have been made to the TAK-700 risks and side effects table:

SWOG Authorized Document

Increase in risk attribution:

- "Increased blood pressure" has been moved from "Occasional, Some May be Serious" to "Common, Some May be Serious."

Decrease in risk attribution:

- "An increase of enzymes in the blood that come from the pancreas (a possible sign of pancreas damage or inflammation)" has been moved from "Occasional, Some May be Serious" to "Rare, and Serious."

This memorandum serves to notify the NCI and the SWOG Statistical Center.

cc. PROTOCOL & INFORMATION OFFICE
Theresa Bucher, RN- Takeda
Elliott Lee- Biologics, Inc.

SWOG Authorized Document

PRIVILEGED COMMUNICATION
FOR INVESTIGATIONAL USE ONLY

Sent for IRB Review: January 22, 2013
Activated March 1, 2013

SWOG

A PHASE III RANDOMIZED TRIAL COMPARING ANDROGEN DEPRIVATION THERAPY + TAK-700 WITH ANDROGEN DEPRIVATION THERAPY + BICALUTAMIDE IN PATIENTS WITH NEWLY DIAGNOSED METASTATIC HORMONE SENSITIVE PROSTATE CANCER

NCT #01809691

STUDY CHAIRS:

Neeraj Agarwal, M.D. (Medical Oncology)
Huntsman Cancer Institute
University of Utah
2000 Circle of Hope, Suite 2123
Salt Lake City, UT 84112-5550
Phone: 801/587-4765
FAX: 801/585-0160
E-mail: neeraj.agarwal@hci.utah.edu (preferred)
E-mail: OncologyUS@gmail.com

Maha H.A. Hussain, M.D. (Medical Oncology)
University of Michigan CCC
Division of Hematology/Oncology
1500 East Medical Center Drive
7314 CCGC
Ann Arbor, MI 48109-0946
Phone: 734/936-8906
FAX: 734/615-2719
E-mail: mahahuss@med.umich.edu

Shilpa Gupta, M.D. (Medical Oncology)
Masonic Cancer Center
University of Minnesota
Division of Hematology/Oncology & Transplantation
420 Delaware Street SE, MMC 480
Minneapolis, MN 55455
Phone: 860/997-2918
FAX: 612/625-6919
E-mail: guptash@umn.edu

Primo N. Lara, Jr., M.D. (Translational Medicine)
UC Davis Cancer Center
4501 X Street
Sacramento, CA 95817
Phone: 916/734-3771
FAX: 916/734-7946
E-mail: pnlara@ucdavis.edu

AGENTS:

IND-Exempt Agents:
Bicalutamide (Casodex™) (NSC-722665)
Goserelin Acetate (Zoladex) (NSC-606864)
Leuprolide Acetate (Lupron) (NSC-377526)

SWOG-Held IND Agents:
TAK-700 (Orteronel) (NSC-757680) (IND-116953)

Amir Goldkorn, M.D. (Translational Medicine-Circulating Tumor Cells)
University of Southern California
Norris Comprehensive Cancer Center
1441 Eastlake Avenue, Suite 3440
Los Angeles, CA 90033-0804
Phone: 323/865-3360
FAX: 323/865-0061
E-mail: agoldkorn@usc.edu

Tony Crispino (Patient Advocate)
8770 S. Tomsik Street
Las Vegas, NV 89113-5222
Phone: 702/917-7779
FAX: 702/974-0312
E-mail: tcneclv@gmail.com

BIOSTATISTICIANS:

Cathy M. Tangen, Dr.P.H. (Biostatistics)
Melissa Plets, M.S.
SWOG Statistical Center
Fred Hutchinson Cancer Research Center
1100 Fairview Avenue North, M3-C102
P.O. Box 19024
Seattle, WA 98109-1024
Phone: 206/667-4623
FAX: 206/667-4408
E-mail: ctangen@fredhutch.org
E-mail: mplets@fredhutch.org

SWOG Authorized Document

<u>ECOG/ACRIN STUDY CHAIR</u>:

Gary R. MacVicar, M.D.
Illinois Cancercare-Peoria
8940 N. Wood Sage Road
Peoria, IL 61615
Phone: 309/243-3000
FAX: 309/243-3049
E-mail: gmacvicar@illinoiscancercare.com

<u>ALLIANCE STUDY CHAIR</u>:

Daniel Vaena, M.D.
University of Iowa Hospitals and Clinics
200 Hawkins Dr., 5970 JPP
Iowa City, IA 52240
Phone: 319/356-3944
FAX: 319/356-7529
E-mail: daniel-vaena@uiowa.edu

SWOG Authorized Document

PARTICIPANTS

ALL NATIONAL CLINICAL TRIALS NETWORK (NCTN) MEMBERS – U.S. INSTITUTIONS ONLY

SWOG/SWOG

ALLIANCE/Alliance for Clinical Trials in Oncology

ECOG-ACRIN/ECOG-ACRIN Cancer Research Group

NRG/NRG Oncology

SWOG Authorized Document

TABLE OF CONTENTS

TITLE 1
PARTICIPANTS 3
TABLE OF CONTENTS 4
CANCER TRIALS SUPPORT UNIT (CTSU) ADDRESS AND CONTACT INFORMATION 6
SCHEMA 7
1.0 OBJECTIVES 8
1.1 Primary Objective 8
1.2 Secondary Objectives 8
1.3 Translational Medicine Objectives 8
2.0 BACKGROUND 8
3.0 DRUG INFORMATION 13
3.1 Bicalutamide (Casodex®) (NSC-722665) 13
3.2 Goserelin Acetate (Zoladex®) D-Ser (But)[6], Azgly[10]) (LHRH) (ICI 118,630) (NSC-606864) 15
3.3 Leuprolide (Eligard®, Lupron Depot®) (NSC-377526) 16
3.4 TAK-700 (Orteronel) (NSC- 757680) (IND-116953) 19
4.0 STAGING CRITERIA 25
5.0 ELIGIBILITY CRITERIA 25
5.1 Disease Related Criteria 25
5.2 Prior Therapy Criteria 25
5.3 Clinical/Laboratory Criteria 26
5.4 Specimen Submission Criteria 28
5.5 Regulatory Criteria 28
6.0 STRATIFICATION FACTORS 28
7.0 TREATMENT PLAN 29
7.1 Treatment 29
7.2 Concomitant Therapy 30
7.3 Drug Compliance Documentation 30
7.4 Criteria for Removal from Protocol Treatment 30
7.5 Discontinuation of Treatment 30
7.6 Follow-Up Period 31
8.0 DOSAGE MODIFICATIONS 31
8.1 NCI Common Terminology Criteria for Adverse Events 31
8.2 General Dose Modification Considerations 31
8.3 Dose Levels of TAK-700 (Orteronel) 31
8.4 Bicalutamide Dose Modifications 31
8.5 TAK-700 (Orteronel) Dose Modifications 32
8.6 Dose Modification Contacts 36
8.7 Adverse Event Reporting 36
9.0 STUDY CALENDAR 37
10.0 CRITERIA FOR EVALUATION AND ENDPOINT ANALYSIS 40
10.1 Measurability of Lesions 40
10.2 Progression Criteria 41
10.3 Symptomatic deterioration 41
10.4 Performance Status 42
10.5 Progression-Free Survival 42
10.6 Time to Death 42
10.7 PSA Response Categories 42
11.0 STATISTICAL CONSIDERATIONS 42
11.1 Accrual Goal 42
11.2 Analysis of Primary Endpoint 43
11.3 Analysis of Secondary Endpoints 44
11.4 Analysis of Translational Medicine (TM) Endpoints 45
11.5 Data and Safety Monitoring Committee Oversight 49
12.0 DISCIPLINE REVIEW 50
13.0 REGISTRATION GUIDELINES 50
13.1 Registration Timing 50

SWOG Authorized Document

13.2 Investigator/Site Requirements 50
13.3 OPEN Registration Requirements 50
13.4 Registration Procedures 52
13.5 Exceptions to SWOG registration policies will not be permitted. 53
14.0 DATA SUBMISSION SCHEDULE 53
14.1 Data Submission Requirement 53
14.2 Master Forms 53
14.3 Data Submission Procedures 53
14.4 Data Submission Overview and Timepoints 54
15.0 SPECIAL INSTRUCTIONS 56
15.1 Androgen Testing (required for the patient) 56
15.2 Specimens for banking and translational medicine studies (optional for the patient) 57
15.3 General Specimen Submission Instructions 58
16.0 ETHICAL AND REGULATORY CONSIDERATIONS 60
16.1 Adverse Event Reporting Requirements 60
17.0 BIBLIOGRAPHY 65
18.0 APPENDIX 68
18.1 Intake Calendar 69
18.2 Translational Medicine Studies 70
18.3 Instructions for blood sample processing, packaging, and shipping for circulating tumor cell (CTC) analysis in **S1216** 101
18.4 New York Heart Association Classifications 103
18.5 Specimen Supplement Sheet 104

SWOG Authorized Document

CANCER TRIALS SUPPORT UNIT (CTSU) ADDRESS AND CONTACT INFORMATION

<table>
<tr><th colspan="3">– CONTACT INFORMATION</th></tr>
<tr><th>For regulatory requirements:</th><th>For patient enrollments:</th><th>For study data submission:</th></tr>
<tr><td>Regulatory documentation can be submitted to the CTSU via:

ONLINE:
Regulatory Submission Portal (Sign in at www.ctsu.org
and select the Regulatory Submission sub-tab under the Regulatory tab.)</td><td>Please refer to the patient enrollment section of the protocol for instructions on using the Oncology Patient Enrollment Network (OPEN) which can be accessed at https://www.ctsu.org/OPEN_SYSTEM/ or https://OPEN.ctsu.org.

Contact the CTSU Help Desk with any OPEN-related questions at ctsucontact@westat.com.</td><td>Data collection for this study will be done exclusively through Medidata Rave. Please see the data submission section of the protocol for further instructions.

<u>Other Tools and Reports</u>:
Institutions participating through the CTSU continue to have access to other tools and reports available on the SWOG Workbench. Access this by using your active CTEP-IAM userid and password at the following url:

https://crawb.crab.org/TXWB/ctsulogon.aspx</td></tr>
<tr><td colspan="3">The most current version of the study protocol and all supporting documents must be downloaded from the protocol-specific Web page of the CTSU Member Web site located at https://www.ctsu.org. Access to the CTSU members’ website is managed through the Cancer Therapy and Evaluation Program - Identity and Access Management (CTEP-IAM) registration system and requires user log on with CTEP-IAM username and password.</td></tr>
<tr><td colspan="3"><u>For patient eligibility or data submission</u> questions contact the SWOG Data Operations Center by phone or email:
206/652-2267
guquestion@crab.org

<u>For treatment or toxicity related</u> questions contact the Study Chair by phone or email: (Dr. Neeraj Agarwal at 801/414-1779).</td></tr>
<tr><td colspan="3"><u>For non-clinical questions (i.e. unrelated to patient eligibility, treatment, or clinical data submission)</u> contact the CTSU Help Desk by phone or e-mail:
CTSU General Information Line – 1-888-823-5923, or ctsucontact@westat.com. All calls and correspondence will be triaged to the appropriate CTSU representative.</td></tr>
<tr><td colspan="3">The CTSU Website is located at https://www.ctsu.org.</td></tr>
</table>

SWOG Authorized Document

SCHEMA

Men with Newly Diagnosed
Metastatic Hormone Sensitive
Prostate Cancer and a
PSA ≥ 2 ng/ml

↓

Registration/Randomization

Arm 1

ADT (LHRH agonist or orchiectomy)
+
TAK-700

Arm 2

ADT (LHRH agonist or orchiectomy)
+
Bicalutamide

Follow Up
10 Years from Registration

SWOG Authorized Document

1.0 OBJECTIVES

1.1 Primary Objective

a. The primary objective of this study is to compare overall survival in newly diagnosed metastatic prostate cancer patients randomly assigned to androgen deprivation therapy (ADT) (LHRHa or orchiectomy) + TAK-700 versus ADT (LHRHa or orchiectomy) + bicalutamide.

1.2 Secondary Objectives

a. To compare progression free survival (as defined in Section 10.5) between the two arms.

b. To compare distributions of PSA response (< 0.2 vs. 0.2-4.0 vs. > 4.0 ng/ml) between the treatment arms at 7 months post-randomization.

c. To compare the qualitative and quantitative adverse events from each treatment arm.

d. To characterize the long-term survival in both treatment arms after 10 years of follow-up.

1.3 Translational Medicine Objectives

a. To validate the prognostic and predictive value of markers of bone turnover in newly-diagnosed metastatic hormone sensitive prostate cancer patients treated with TAK-700.

b. To bank plasma/whole blood and tissue specimens for future use.

c. To evaluate genomic variants and gene expression of androgen pathway genes and their correlation with response to therapy (ADT + TAK-700 vs. ADT + bicalutamide).

2.0 BACKGROUND

Prostate cancer is the most common non-cutaneous malignancy among American men. *(1)* While the majority of men are diagnosed in early stages, about one third of these patients will eventually develop metastatic disease. The cornerstone of treatment for metastatic prostate cancer is androgen deprivation therapy (ADT). Despite initial responses, almost all patients will eventually develop disease progression. Historically, disease progression despite continuous ADT has been defined as hormone resistant or androgen independent disease. Over the last decade, it has been recognized that despite the failure of ADT, these tumors are in part still dependent on androgen receptor (AR) signaling for proliferation. *(2)* Hence, this stage of prostate cancer is not always hormone resistant or androgen independent. The more suitable term is thus castration resistant prostate cancer (CRPC), since the resistance occurs at castrate androgen levels and not in the complete absence of androgens or androgen signaling. Indeed, many CRPC patients will still respond, in cases for a number of years, to sequential administration of secondary hormonal manipulations. (3) Several mechanisms have been identified to explain persistent androgen signaling in CRPC. *(4,5)* These include increased AR gene expression, either due to AR gene amplification, an increased rate of transcription of AR gene or from increased stability of the AR transcript. (6,7) One known (but rare) mechanism is mutation of the AR gene, resulting in promiscuous ARs, which are activated by antiandrogens (such as bicalutamide) and other endogenous steroids (such as progesterone or deoxycorticosterone). (8,9) MDV 3100 (Medivation Inc, San Francisco, CA) is an orally

SWOG Authorized Document

administered novel AR antagonist that binds to AR with a much higher affinity than bicalutamide, blocks nuclear translocation of AR and DNA binding, but does not have any effect on androgen synthesis. (10) In a recently reported Phase III trial of 1199 men with progressive metastatic CRPC with prior docetaxel therapy, overall survival was significantly improved in those treated with MDV 3100 compared to placebo (18.4 vs. 13.6 months, HR=0.63). These data establish AR signaling as a valid target despite ongoing castration. (11)

Persistent intratumoral androgen synthesis despite castration promotes prostate cancer progression: Although ADT reduces the level of serum androgens to very low levels, it does not eliminate them completely. In castrate patients, adrenal glands are the major source of androgens. In addition, there is evidence that prostate cancer cells can not only initiate aberrant androgen signaling, but can also synthesize sufficient amounts of intratumoral androgens to allow continued androgen signaling and tumor growth in a castrate patient. *(12)* The concentrations of intratumoral testosterone in metastatic prostate tumors in men with CRPC are two to three times higher than in prostate tumors in men who have never received ADT despite the fact that those not receiving ADT have much higher serum testosterone levels. *(13,14)* After receiving nine months of neoadjuvant deprivation therapy, men with localized prostate cancer did not have a reduction in the expression of many androgen-responsive genes, including the AR and prostate-specific antigen (PSA), although intratumoral testosterone and DHT levels were reduced by 75%. *(15)* Many enzymes involved in androgen synthesis are highly up-regulated in CRPC compared to those with androgen sensitive prostate cancer. *(16)* Notably, CRPC cell lines synthesized a five fold-higher concentration of testosterone than androgen dependent cell lines and were capable of directly converting radioactive cholesterol into testosterone in vitro. *(17)* These data indicate that castration based on serum testosterone levels is not synonymous with androgen ablation in the prostate tumor microenvironment. Prostate cancer cells can adapt to castration by intratumoral synthesis or conversion of adrenal androgens to testosterone and DHT and subsequently derive growth advantage. Therefore, enzymes of the androgen synthesis pathway provide multiple and attractive therapeutic targets in patients with CRPC.

Rationale for the use of a selective CYP 17, 20 lyase inhibitor: The first step in adrenal steroid biosynthesis is the formation of cholesterol from acetyl Co A and squalene. Cholesterol is then converted to pregnenolone and then to progesterone. The pivotal enzyme in androgen synthesis is the CYP17 (CYP 17 hydroxylase, CYP17, 20 lyase) located in the Leydig cells of the testes and zona fasiculata and reticularis of the adrenal glands. This enzyme catalyzes conversion of pregnenolone and progesterone to the weak androgen steroids, dehydroepiandrosterone (DHEA) and androstenedione, respectively. Both DHEA and androstenedione are eventually converted to testosterone and then to DHT, reactions catalyzed by other enzymes. Because of its critical role in the synthesis of androgens in the adrenal glands, testes and prostate, CYP17 was deemed to be an attractive therapeutic target in patients with CRPC. A novel CYP17 inhibitor, abiraterone acetate has recently been shown to be safe and efficacious in Phase II trials. In two Phase II trials of patients with castration refractory chemotherapy naïve prostate cancer, PSA response rates (≥ 50%) were seen in 67% and 85% of patients respectively. *(18,19)* A Phase III trial of post-docetaxel therapy with abiraterone has recently reported positive for overall survival with a 3.9 month prolongation of median survival (HR 0.65, P <0.0001). *(20)* These data provide clinical evidence that androgen signaling remains active in prostate cancer despite castration and CYP17 inhibition can be used to optimize androgen blockade even in androgen sensitive prostate cancer. The majority of side effects in these trials were because of inhibition of CYP17 hydroxylase. This enzyme mediates production of cortisol, leading to secondary mineralocorticoid excess due to excess ACTH and subsequent increase in corticosterone and aldosterone.

Rationale for the use of TAK-700: TAK-700, like abiraterone, is a novel inhibitor of CYP17 pathway. However, TAK-700 more specifically inhibits CYP17, 20 lyase versus CYP17 hydroxylase and does not generally lead to the syndrome of secondary mineralocorticoid excess.

SWOG Authorized Document

In men with prostate cancer, TAK-700 effectively lowers testosterone and DHEA-S concentrations below the concentrations observed in men who have experienced medical or surgical castration. Preliminary dose finding suggest that doses of TAK-700 at 300 mg BID or above will be effective for this pharmacodynamic effect. Glucocorticoid supplementation to suppress ACTH-adrenal activation will likely be required at doses of 400 mg BID or above. Single and multiple doses of TAK-700 (up to 600 mg) appeared to be safe and well tolerated in Phase I studies conducted with TAK-700 in healthy male subjects. The most common adverse effect (AE) observed in the completed Phase I study was headache. In Study TAK-700_201, no specific dose-limiting toxicity was identified in subjects with metastatic CRPC. The most frequently reported treatment emergent adverse effects (TEAEs) were fatigue, gastrointestinal disorders, and headache.

Updated data of the Phase II portion of a Phase I/II study of TAK-700 in chemonaive patients with metastatic CRPC were reported in 2012 Genitourinary Cancers Symposium. *(21)* Ninety-seven (97) patients were treated with TAK-700 in four different dose cohorts: 300 mg BID (n=23), 400 mg BID + prednisone 5 mg BID (n=24), 600 mg BID + prednisone (n=26), or 600 mg QD (n=24). At data cut-off (23 May 2011), 62% of patients had withdrawn (including 19% due to adverse effects and 19% for disease progression). The most common adverse effects were fatigue (76%), nausea (47%), and constipation (38%); most common Grade ≥ 3 adverse effects were fatigue (12%) and hypokalaemia (8%). PSA response rates (≥ 50%) at 12 weeks were 63%, 50%, 41%, and 60% in the 300 mg BID, 400 and 600 mg BID + prednisone, and 600 mg QD groups, respectively. Of 51 RECIST-evaluable patients, 10 had partial responses (of which 5 were confirmed), 22 stable disease, and 15 progressive disease. At 12 weeks, median testosterone decreased from baseline in all groups: (ng/dL, 12 weeks/baseline) 0.98/8.50 (300 mg BID), 0.30/9.90 (400 mg BID +prednisone), 0.07/7.33 (600 mg BID + prednisone), 0.49/6.31 (600 mg QD). Similarly, at 12 weeks, median DHEA-S decreased from baseline in all groups: (µg/dL, 12 weeks/baseline) 8.65/53.0 (300 mg BID), 0.10/36.3 (400 mg BID + prednisone), 0.10/51.7 (600 mg BID + prednisone), 5.30/31.5 (600 mg QD). Overall, mean circulating tumor cell numbers decreased from 16.6 (per 7.5 mL blood) at baseline to 3.9 at 12 weeks. TAK-700 ≥ 300 mg BID appeared active and well tolerated in patients with metastatic CRPC, with similar efficacy, with and without prednisone.

In a Phase II study in non-metastatic CRPC with biochemical recurrence alone, 38 patients were treated with TAK-700 300 mg twice daily without prednisone. *(22)* Treatment was feasible with manageable toxicities. After three months of treatment, 16% achieved PSA ≤ 0.2 ng/Ml, 76% achieved ≥ 50% decrease and 32% achieved a PSA reduction of ≥ 90%. Median time to PSA progression was 14.8 months. TAK-700 without prednisone suppressed adrenal androgens by 85–90%. Only one patient had laboratory values consistent with a hypoadrenal state for which he received corticosteroid replacement.

In a Phase III trial of metastatic CRPC with prior docetaxel therapy (ELM-PC 5 trial), 1,099 men were randomized 2:1 to TAK-700 (orteronel) 400 mg twice daily + prednisone 5 mg twice daily versus placebo + prednisone 5 mg twice daily. The primary endpoint was overall survival (OS). Other key endpoints were radiographical progression-free survival (rPFS), 50% or more PSA decrease at 12 weeks, pain response at 12 weeks, and safety. Results were presented in the 2014 Genitourinary Oncology Symposium. *(23)* The study was terminated for failing to meet its primary endpoint: median overall survival (OS) was 17.0 months (95% CI 15.2, 19.9) in patients receiving orteronel versus 15.2 months (95% CI 13.5, 16.9) in those receiving placebo (HR: 0.886 [95% CI: 0.739, 1.062]; *P*=0.1898). Substantial regional differences were seen in OS benefit: median OS (orteronel vs. placebo) was 20.9 vs. 16.9 mo (HR: 0.889) in North America (n=112), 18.3 vs. 17.8 mo (HR: 1.048) in Europe (n=590), and 15.3 vs 10.1 mo (HR: 0.709) in the rest of the world (n=397). In the overall population, rPFS was significantly improved in the orteronel arm, with a median of 8.3 months vs. 5.7 months in the placebo arm (HR: 0.76 [95% CI: 0.653, 0.885]; *P*=0.00038). Drug-related adverse events (any grade) included (orteronel/placebo) nausea (30/16%), vomiting (23/8%), fatigue (17/11%), and diarrhea (16/9%); Grade 3 or higher

SWOG Authorized Document

drug-related adverse events included increased lipase (12/less than 1%), increased amylase (8/less than 1%), and fatigue (3/3%). While orteronel + prednisone did not show a statistically significant OS improvement versus placebo + prednisone, rPFS findings and striking regional OS differences suggest that orteronel has clinically meaningful activity.

In the second Phase III trial in chemonaïve men with metastatic CRPC, 1560 men were randomized 1:1 to treatment with orteronel 400 mg twice daily + prednisone 5 mg twice daily or placebo + prednisone. Co-primary endpoints were OS and rPFS. Results were presented in the 2014 ASCO annual meeting. *(24)* The study met the co-primary endpoint of rPFS. At the final analysis for OS the updated median rPFS benefit with orteronel vs. placebo was significantly higher (median 13.8 vs 8.7 months; HR 0.7; 95% CI: 0.6–0.8; *P* < .00001). The co-primary endpoint of OS was not met. Median OS with orteronel vs placebo was 31.4 vs 29.5 months (HR 0.9; 95% CI: 0.8–1.1; *P*=0.314), with no notable differences across regions. More patients had a ≥ 50% PSA decrease (43% vs 25%, *P* < .00001) and a favorable circulating tumor cell count (15% vs 9%, *P*=0.00016) at 12 weeks with orteronel vs placebo. Common all-grade adverse events with orteronel vs. placebo included nausea (36% *vs* 15%), fatigue (34% *vs* 20%), constipation (33% *vs* 15%), and diarrhea (28% *vs* 14%); 30% *vs* 18% of pts discontinued due to adverse events. Similar proportions of patients in both arms received post trial therapy with docetaxel, abiraterone, and enzalutamide.

The results of the trials of TAK-700 in the mCRPC setting may not be fully applicable to the mHSPC setting, and testing novel AR synthesis blockers remains a valid strategy in the mHSPC setting where their effect may be more profound. For example, docetaxel improved survival by ~ 3 months in the mCRPC setting, but by ~14 months in the mHSPC setting in the CHAARTED study (described later). In addition, the rPFS HR for abiraterone in the post-chemotherapy COU-302 trial (0.63) and TAK-700 in ELM-PC-5 (0.70) overlap with a numerical difference potentially due to differences in patient population and study design.

Rationale for comparing androgen deprivation therapy + TAK-700 with androgen deprivation therapy + bicalutamide in androgen sensitive metastatic prostate cancer: Survival benefit of adding anti-androgen to castration was first shown by the SWOG-coordinated US Intergroup study SWOG-8494 (INT-0036), which randomly assigned 603 men with newly diagnosed hormone sensitive metastatic prostate cancer, to combine androgen blockade (CAB) with leuprolide plus flutamide or leuprolide alone. *(25)* When compared to leuprolide alone, men treated with CAB had significantly longer progression-free and median survival (16.5 vs. 13.9 months, and 35.6 vs. 28.3 months, respectively). However, in a subsequent Phase III trial (SWOG-8894, INT-0105) with 1387 men, addition of flutamide to bilateral orchiectomy did not result in a clinically meaningful improvement in survival. *(26)* Although more patients treated with CAB achieved a serum PSA < 4 ng/mL (74 versus 62 percent with placebo), the differences in median and progression-free survival were not statistically significant (34 versus 30 months, and 20 versus 19 months, respectively). Since then, several meta-analyses have shown improvement in survival outcomes with CAB over castration, although with increased toxicity. *(27,28,29)* The ASCO guidelines published in 2007 state that CAB should be considered an option, and be discussed with the patients with the emphasis that improved overall survival may occur at the cost of higher toxicity. *(30)*

Klotz et al recently reported a post hoc analysis of clinical outcomes according to testosterone levels achieved after initiation of first-line ADT among 696 patients treated with continuous ADT in this study. This PR-7 study was a Phase III trial, where men with biochemically recurrent prostate cancer after primary local therapy were randomized to either continuous or intermittent ADT (with 8-month treatment cycles). Serum testosterone was measured prospectively every 2 months during the first year on study. In this report, the men whose testosterone nadir after ADT initiation fell below 20 ng/dL fared better in terms of time to castration resistance (defined as three increasing PSA values) in the setting of serum testosterone < 85 ng/dL and cancer-specific survival compared with those who did not achieve this degree of castration. In addition, men whose testosterone spiked above 50 ng/dL during the course of ADT treatment developed

SWOG Authorized Document

castration resistance more quickly than those whose testosterone remained persistently below 20 ng/dL. *(31)* These data from PR-7 study, represent some of the strongest evidence to date that failure to achieve deep testosterone suppression after first-line ADT may herald poor outcomes in men with hormone-sensitive non-metastatic prostate cancer. *(32)* These data provide a compelling rationale for developing a more efficacious treatment regimen, for a deeper androgen blockade in the hormone sensitive prostate cancer setting and to improve survival outcome in these patients.

Evidence of superior blockade of androgen synthesis by TAK-700 provides the basis for its inclusion in the novel combination regimen, i.e. ADT and TAK-700, which in turn, is expected to provide a superior androgen blockade over a combination of ADT and bicalutamide. In the SWOG-9346 trial, the depth of PSA response (i.e. < 0.2, 0.2-4 or > 4 ng/ml at 7 months) predicted overall survival. *(33)* Thus, the combination of ADT and TAK-700 is expected to improve survival by providing a superior androgen blockade and improving PSA response in patients with newly diagnosed hormone sensitive metastatic prostate cancer.

<u>Role of chemotherapy with docetaxel in new hormone sensitive metastatic prostate cancer:</u> ECOG 3805/CHAARTED is an intergroup Phase III study comparing ADT, with or without chemotherapy with docetaxel, with prednisone in men with hormone sensitive metastatic prostate cancer (mHSPC). The study enrolled 790 men with mHSPC between July 2006 and November 2012. Approximately two-thirds of patients had a high extent of disease which, according to the study, meant the disease involved the liver, four or more bones, or both. Results of the study were presented in the 2014 ASCO annual meeting. *(34)* There was a significant improvement in the overall survival (OS) favoring the participants who had received docetaxel chemotherapy in addition to the ADT compared to the ADT alone (57.6 vs. 44 months, HR 0.63; 95% CI: 0.48, 0.82; $P = 0.0006$). Further analysis showed that patients with a high extent of metastatic disease accounted for most of the benefit in the OS from docetaxel plus ADT (49.2 vs. 32.2 months, HR 0.62; 95% CI: 0.46, 0.83; $P = 0.0012$). Median follow-up at the time of report was 29 months, and at this time, the median survival in the low extent disease patients both arms had not been reached. These data support the use of front line docetaxel with androgen deprivation therapy for patients with newly diagnosed mHSPC who, on the basis of clinical judgement, have high risk or extensive disease, and are deemed to be suitable candidates for docetaxel per the treating physician's clinical judgment.

<u>Plausibility of seeing a 33% improvement in survival:</u> Hussain and colleagues have reported, based on data from over 1000 patients with new M1 prostate cancer undergoing ADT (with goserelin and bicalutamide) per SWOG-9346, that failure to achieve a PSA of ≤ 4 ng/ml (or to be experiencing a rise in PSA) after 7 months of combined ADT is a very powerful negative predictor for survival. *(35)* The median overall survival for this group of patients was 20 months from the start of androgen deprivation therapy. This is to be contrasted with the 45% of the patients who achieved undetectable PSA levels (≤ 0.2 ng/ml) at month 7 whose median overall survival was of 82 months from the start of androgen deprivation. Because of a superior blockade of androgen synthesis by TAK-700 over bicalutamide, the combination of ADT + TAK-700 is expected to result in a 15% improvement (45% to 60%) in PSA response of ≤ 0.2 ng/ml at 7 months of therapy over ADT + bicalutamide arm. This, in turn, is expected to result in 33% improvement in overall survival with the experimental regimen (i.e. ADT + TAK-700).

<u>Potential impact of the trial:</u> Improved overall survival will benefit hundreds of thousands of patients over the years. In addition, improved progression free survival will improve quality of life by delaying the use of chemotherapy. Hence, if this trial is positive, TAK-700 with ADT will become the standard of care patients with newly diagnosed metastatic prostate cancer.

<u>Inclusion of Minorities:</u>

This study was designed to include minorities, but was not designed to measure differences of intervention effects. SWOG is unaware of any literature supporting an interactive treatment effect by race; therefore, there is no current plan to alter the accrual for separate racial subsets. The Group is committed to the continued accrual of non-white patients to all of its trials at current

SWOG Authorized Document

levels or better, and will explore the effect of treatment by race in these trials. Anticipated accrual to this study by race and ethnicity, based on previous Group trials in this disease type, follows:

**Ethnic Category**	**Females**	**Males**	**Total**
Hispanic or Latino	0	29	29
Not Hispanic or Latino	0	1275	1275
**Total Ethnic**	0	1304	1304
**Racial Category**			
American Indian or Alaskan Native	0	6	6
Asian	0	17	17
Black or African American	0	220	220
Native Hawaiian or other Pacific Islander	0	3	3
White	0	1058	1058
**Racial Category: Total of all Subjects**	0	1304	1304

3.0 DRUG INFORMATION

Investigator's Brochures

For information regarding Investigator's Brochures, please refer to SWOG Policy 15.

For this study, bicalutamide and the LHRH agonist (goserelin acetate or leuprolide acetate) are commercially available; therefore, Investigator Brochures are not applicable to these drugs. Information about commercial drugs is publicly available in the prescribing information and other resources.

Each investigator who plans to register patients to this study must obtain and complete a Confidentiality Disclosure Agreement (CDA) with SWOG and submit it via the CTSU Regulatory Office (https://www.ctsu.org) as a protocol specific requirement prior to submitting this study for IRB review.

For this study, TAK-700 is investigational and is being provided under an IND held by SWOG. For INDs filed by SWOG, the protocol serves as the Investigator Brochure for the performance of the protocol. In such instances submission of the protocol to the IRB should suffice for providing the IRB with information about the drug. However, in cases where the IRB insists on having the official Investigator Brochure, it will be distributed to sites upon request by the SWOG Operations Office (210/614-8808) after verification of CDA receipt.

3.1 Bicalutamide (Casodex®) (NSC-722665)

a. DESCRIPTION

Chemistry: Bicalutamide is a racemic mixture containing two enantiomers, (2RS)-4'-Cyano-3(4-fluorophenylsulphonyl)-2-hydroxy-2-methyl-3'-trifluoromethyl) propionanilide.

Bicalutamide is an active non-steroidal antiandrogen and its antiandrogen activity resides exclusively in the (-) or (R) enantiomer. Unlike flutamide, it is peripherally selective and does not cause a rise in serum LH or Testosterone in male rats and dogs. This peripheral selectivity may be because it penetrates poorly the CNS and Hypothalamus (the site of negative feedback of androgens). In humans,

SWOG Authorized Document

rises in LH, Testosterone and Estradiol concentrations were seen. These rises were not dose related. In 90%, testosterone levels remained within normal limits. There was no significant rise in mean serum FSH.

b. TOXICOLOGY

In rats, besides antiandrogenic changes, there was evidence of hepatocyte hypertrophy and basophilia. In dogs treated for 6 months, there was increased heart rate with decreased PR interval, transient decrease in circulating PMNs and increased plasma cholesterol. No cardiac pathology was found. In a mouse oncogenicity study, an increased incidence of hepatocellular carcinoma was observed in the top dose male group (75 mg/kg/day). The no effect dose level for hepatocellular carcinoma in this study was 15 mg/kg/day with steady state blood levels in excess of 10 µg/ml. The mechanism for this tumor formation is a non-genotoxic, phenobarbitone-type MFO induction and is not considered to represent a risk for humans. A two-year study in rats and female mice at similar doses did not show an increased incidence of hepatic tumors.

Bicalutamide has been given to over 3,500 men in 35 different clinical studies worldwide, in doses up to 600 mg daily. When bicalutamide is given in combination with an LHRH analog, the pharmacologic adverse event profile is dominated by the LHRH analog and includes hot flashes (53%), gynecomastia (9%) and breast pain (6%). Other adverse events reported regardless of causality included diarrhea (12%), constipation (22%), nausea (15%) and abdominal pain (11%). Other adverse were reported, such as fatigue (22%), pain (35%), back pain (25%), pelvic pain (21%), infection (18%), peripheral edema (13%), dyspnea (13%), nocturia (12%), hematuria (12%), anemia (11%), dizziness (10%). Bicalutamide has been associated with changes in liver function, although these are infrequent (7%) and rarely occur with jaundice. Many of these changes improved or resolved despite continuation of bicalutamide therapy. There have been no reports of fatal hepatotoxicity associated with bicalutamide therapy.

c. PHARMACOLOGY

Pharmacokinetics:

Animal studies: After oral single dose administration, absorption of the compound was slow with peak concentration occurring 3 - 12 hours and plateau between 2 and 48 hours. There was non-proportional increase in plasma levels with increasing doses. Elimination half life ranges from 17 - 28 hours in male rats, 21 - 29 hours in female rats and 5 - 7.5 days in dogs. 91 - 96% of bicalutamide is bound to plasma protein.

Human studies: After single doses, mean time for peak plasma concentration was 6 hours at 10 and 30 mg, but at 50 mg, it was 16 hours. Mean plasma elimination half lives after 12 weeks of 10, 30, 50, 100 mg/day was 7 - 10 days. This finding was consistent with single dose data. In patients given daily doses of 50 mg, mean plasma concentration was 10 ug/ml at 12 weeks. After single doses, there was linear increase with doses between 10 and 50 mg, but became non-linear at doses of 50 - 100 mg. At 100 mg, the oral bioavailability is reduced by 30% but plasma elimination half life is unchanged. Bicalutamide is extensively metabolized and metabolites are excreted by both the biliary and urinary system.

Formulation: Bicalutamide is prepared as round, film-coated green or white tablets containing standard excipients and 50 mg of the drug.

SWOG Authorized Document

Storage and stability: All packages of bicalutamide should be stored securely in a dry place at room temperature.

Route of Administration: Bicalutamide is to be administered in tablet form as a once-daily oral dose. Patients should be instructed to take one tablet once daily.

Supplier: Bicalutamide is commercially available and should therefore be purchased by a third party. This drug will not be supplied by the NCI.

Please refer to the package insert for complete information.

3.2 Goserelin Acetate (Zoladex®) D-Ser (But)^6, Azgly^{10}) (LHRH) (ICI 118,630) (NSC-606864)

a. DESCRIPTION

Chemistry: The physical form of goserelin acetate is an off-white powder.

Chemical Structure:

pyro-Glu-His-Trp-Ser-Tyr-D-Ser (Bu^t)-Leu-Arg-Pro-Azgly-NH$_2$

Molecular Weight: 1269 (as base)

Solubility: Soluble in water, dimethylformamide and dimethylsulfoxide.

b. TOXICOLOGY

Animal Studies: In laboratory animals, the acute toxicity of ICI 118,630 was found to be very low in comparison to its pharmacologic potency. Intravenous doses of up to 6 mg/kg were without any adverse effects in rats undergoing LD50 studies. No significant pharmacological activity was apparent in the cardiovascular, respiratory, central nervous, renal and metabolic, coagulation or gastric acid secretory systems. In rats, but not the cat, mouse, dog or rabbit, long-term dosing of goserelin acetate has resulted in benign pituitary adenomas.

Human Toxicity: Most side effects attributed to LHRH agonists have been due to testosterone withdrawal (e.g., hot flashes, sweats, impotence, breast enlargement, nausea and dizziness). Skin reaction and irritation at the injection site (rarely, < 2%) have also been observed. Tumor flares have been reported in a small number of patients receiving LHRH agonists. Flare reactions usually consist of minor complications such as increased bone pain and therapy may be continued; however, potentially dangerous complications (e.g. incipient spinal cord compression or ureteral obstruction) must be averted by discontinuing goserelin acetate treatment and beginning corrective therapy immediately.

c. PHARMACOLOGY

Kinetics: Subcutaneous depot given on Days 1 and 29 to patients with prostatic cancer with normal renal and hepatic function revealed a peak concentration 12 to 15 days after administration, with peak values of 2.64 ng/ml and 2.44 ng/ml. There was no drug accumulation. In an aqueous formulation given daily to 8 patients with advanced prostatic cancer, peak serum concentration was 12.8 ng/ml and the half life was 6.1 hours. The mean area under the curve in the dosing interval was 65 ng h/ml and mean total body clearance was 120.1 ml/min.

Formulation: Goserelin acetate is supplied as a 10.8 mg solid depot formulation preloaded in a disposable syringe device mounted on a #14 gauge hypodermic needle.

SWOG Authorized Document

Storage and Stability: The sterile unit will be enclosed in a sealed light and moisture proof package. The package should be stored securely in a dry place at room temperature (not to exceed 25°C or 77°F). Before being opened, each package must be inspected for damage in which case the depot should not be used. Being sterile, the syringe should be removed from its package only immediately before required.

Administration: Goserelin acetate will be injected every 3 months. No anesthetic is required; however, local anesthetics may be applied prior to injection if desired. After cleaning with an alcohol swab, a small area of skin on the anterior abdominal wall will be anesthetized by injecting 0.2 ml of 1.0% lidocaine hydrochloride intradermally or by applying topical ethyl chloride. Goserelin acetate will then be injected into the subcutaneous fat using aseptic technique. After injecting goserelin acetate depot, for the first time only, the overlying skin will be indelibly marked with a single spot. In the unlikely event of the depot needing to be surgically removed (e.g., severe anaphylaxis), this mark will facilitate such a procedure. After checking to ensure that the depot has been discharged, the used syringe will be disposed of per local institutional guidelines.

Supplier: Goserelin acetate is commercially available and should therefore be purchased by a third party. This drug will not be supplied by the NCI.

Please refer to the package insert for complete information.

3.3 Leuprolide (Eligard®, Lupron Depot®) (NSC-377526)

a. DESCRIPTION

Leuprolide is synthetic nonapeptide luteinizing hormone-releasing hormone (LHRH) agonist.

1. Molecular Formula: C59H84N16O12

2. Molecular Weight: 1209.4

b. PHARMACOLOGY

Mechanism of Action: Leuprolide inhibits gonadotropin secretion by acting as an luteinizing hormone-releasing hormone (LHRH) agonist. Continuous administration results in suppression of ovarian and testicular steroidogenesis due to decreased levels of LH and FSH with subsequent decrease in testosterone (male) and estrogen (female) levels. In males, testosterone levels are reduced to below castrate levels. Leuprolide may also act directly on the testes as well as act by a different mechanism not directly related to reduction in serum testosterone.

c. PHARMACOKINETICS

1. Absorption: After the initial increase of leuprolide following each injection, mean serum concentrations remain relatively constant.

2. Distribution: The mean steady-state volume of distribution of leuprolide following intravenous bolus administration to healthy male volunteers was 27 L. In vitro binding to human plasma proteins ranged from 43% to 49%.

SWOG Authorized Document

3. Metabolism: Upon administration with different leuprolide acetate formulations, the major metabolite of leuprolide acetate is a pentapeptide (M-I) metabolite.

4. Elimination: Less than 5% of the leuprolide dose was recovered as parent and M-I metabolite in the urine following the 3.5 mg depot injection.

d. ADVERSE EFFECTS

1. Refer to the package insert or manufacturer website for the most complete and up to date information on contraindications, warnings and precautions, and adverse reactions.

**Adverse Events with Possible Relationship to Leuprolide**		
**Likely (>20%)**	**Less Likely (≤20%)**	**Rare but Serious (<3%)**
BLOOD AND LYMPHATIC SYSTEM DISORDERS		
	Edema	
CARDIAC DISORDERS		
	Hyper- / hypotension	Arrhythmia
	Tachycardia	Atrial fibrillation
	Bradycardia	Congestive heart failure
	Angina	Syncope
	Palpitation	
GASTROINTESTINAL DISORDERS		
Nausea	Altered bowel function	Gastrointestinal hemorrhage
Vomiting	Ulcer	
	Intestinal obstruction	
	Constipation	
	Diarrhea	
	Gastroenteritis/ colitis	
GENERAL DISORDERS AND ADMINISTRATION SITE CONDITIONS		
Local injection site burning/stinging	Skin reaction	
IMMUNE SYSTEM DISORDERS		
	Flu-like syndrome	Allergic reaction
INFECTIONS AND INFESTATIONS		
	Urinary tract infection	
	Infection	
INVESTIGATIONS		
	BUN increased	
	Creatinine increased	
	Bicarbonate decreased	
	Hyperuricemia	
	Hypoproteinemia	
	Hypoalbuminemia	
METABOLISM AND NUTRITION DISORDERS		
	Dehydration	
	Hyperlipidemia	
	Weight gain/loss	
MUSCULOSKELETAL AND CONNECTIVE TISSUE DISORDERS		
	Weakness	
	Bone pain	

SWOG Authorized Document

	Joint disorder	
	Myalgia	
	Paresthesia	
NERVOUS SYSTEM DISORDERS		
Headache	Nervousness	Seizure
Pain	Anxiety	
Insomnia	Confusion	
	Fatigue	
	Dizziness/vertigo	
PSYCHIATRIC DISORDERS		
Depression		
RENAL AND URINARY DISORDERS		
	Urinary disorders	
	Bladder spasm	
	Urinary retention	
REPRODUCTIVE SYSTEM AND BREAST DISORDERS		
Hot flashes / sweats	Vaginitis	
Testicular atrophy	Gynecomastia	
	Breast tenderness	
	Testicular Pain	
	Menstrual disorder	
	Lactation	
	Impotence	
	Libido decreased	
	Nocturia	
RESPIRATORY, THORACIC AND MEDIASTINAL DISORDERS		
	Emphysema	
	Epistaxis	
	Pleural effusion	
	Pulmonary edema	
	Dyspnea	
	Cough	
SKIN AND SUBCUTANEOUS TISSUE DISORDERS		
	Acne	
	Alopecia	
	Bruising	
	Cellulitis	
	Pruritus	
	Rash	
	Hirsutism	
VASCULAR DISORDERS		
	Varicose vein	
	Deep thrombophlebitis	

Adverse events occurring in < 1%, postmarketing, and/or case reports: Abdominal pain, anaphylactic/anaphylactoid reactions, asthmatic reactions, bone density decreased, coronary artery disease, diabetes; fibromyalgia-like symptoms; flushing, hemoptysis, hepatic dysfunction, hypokalemia, hypoproteinemia, injection site induration/abscess, liver injury, myocardial infarction, pelvic fibrosis, penile swelling, peripheral neuropathy, photosensitivity; pituitary apoplexy; prostate pain, pulmonary embolism, pulmonary infiltrate, seizure, spinal fracture/paralysis, stroke suicidal ideation/attempt (rare), tenosynovitis-like symptoms, thrombocytopenia, transient ischemic attack, uric acid increased, urticaria, WBC decreased/increased.

SWOG Authorized Document

Androgen deprivation therapy may prolong the QT/QTc interval. Patients with congenital long QT syndrome, congestive heart failure, frequent electrolyte abnormalities, and patients taking drugs known to prolong the QT interval may be at increased risk.

2. Pregnancy and Lactation: Leuprolide is pregnancy category X and excretion into breast milk is unknown/contraindicated.

3. Drug Interactions: Luteinizing hormone-releasing hormone analogs may diminish the therapeutic effect of antidiabetic agents. No pharmacokinetic-based drug-drug interaction studies have been performed. Because leuprolide is a peptide that is primarily degraded by peptidase and not by Cytochrome P-450 enzymes and the drug is only about 46% protein bound, drug interactions would not be expected to occur.

e. DOSING & ADMINISTRATION

1. Dosing – See Treatment Plan

2. Leuprolide is administered intramuscular (Lupron Depot®) or subcutaneous (Eligard®) injection based on commercial depot formulation. Injection sites should be varied periodically.

f. STORAGE & STABILITY

Store at room temperature 25°C (77°F); excursions permitted to 15-30°C (59-86°F) [see USP Controlled Room Temperature].

g. HOW SUPPLIED

1. Leuprolide acetate is available in 3.75 mg, 7.5 mg, 11.25 mg, 22.5 mg, 30 mg, or 45 mg depot formulation kit with accompanying diluent. The prefilled dual-chamber syringe contains lyophilized microspheres of leuprolide acetate incorporated in a biodegradable lactic acid polymer.

2. Leuprolide is commercially available and will not be supplied. Please refer to the current FDA-approved package insert for additional information.

3.4 TAK-700 (Orteronel) (NSC- 757680) (IND-116953)

a. PHARMACOLOGY

TAK-700 (orteronel) is an oral, selective, nonsteroidal 17, 20-lyase inhibitor that blocks the synthesis of gonadal and adrenal androgens (dehydroepi-androsterone, testosterone, dehydroepiandrosterone sulfate, and androsten-edione). This leads to a reduction in sex hormone levels in the circulation and hormone-dependent malignant tissue.

b. PHARMACOKINETICS

1. <u>Absorption</u>: Following a single oral and IV dose of [^{14}C]orteronel, the AUC ratios (PO [measure from portal vein concentration]:IV) for [^{14}C]orteronel were 41.4% and 69.9% in rats and monkeys, respectively. The bioavailability of [^{14}C]orteronel was 41.0% and 70.5% in rats and monkey, respectively, indicating that first-pass metabolism is negligible in both species.

SWOG Authorized Document

In humans, the presence of food, regardless of fat content, increased the AUC of TAK-700 by approximately 40% and nearly doubled the C_{max} levels. No dose administration recommendations with regard to food were deemed necessary in ongoing Phase III studies.

2. Distribution: Following a single oral dose of [^{14}C]orteronel to male and female rats, ^{14}C achieved maximum concentrations at 0.5 or 2 hours post-dose and decreased to minimal concentrations within 168 hours in most tissues. The lowest concentrations of ^{14}C were in the brain and spinal cord.

In humans, the mean volume of distribution ranged from 387.3 to 655.7 L.

3. Metabolism: TAK-700 is metabolized by hydrolysis of the amide bond to its active metabolite, M-I. There is minimal involvement of cytochrome P450 isozymes.

4. Elimination: TAK-700 and its metabolite M-I are primarily eliminated in the feces in rats and urine in monkeys, within 120 and 72 hours, respectively.

In humans, the mean terminal half-life of TAK-700 ranged from 12.7 to 15 hours. The mean half-life of the primary metabolite, M-I, was similar and ranged from 12.5 to 15.9 hours. The mean clearance ranged from 20.7 to 30.5 L/hr. Urinary excretion is the primary mean of elimination, with approximately half of a single dose excreted unchanged in the urine. The effect of renal insufficiency on TAK-700 pharmacokinetics has not yet been studied.

c. Adverse effects

1. Human Data:

Adverse Events with Possible Relationship to Orteronel (TAK-700)		
**Likely (>20%)**	**Less Likely (≤20%)**	**Rare but Serious (<3%)**
CARDIAC DISORDERS		
		Sinus tachycardia
ENDOCRINE DISORDERS		
		Adrenal insuffiency
		Androgen deficiency
GASTROINTESTINAL DISORDERS		
Nausea		Pancreatitis
Vomiting		
Constipation		
Diarrhea		
GENERAL DISORDERS AND ADMINISTRATION SITE CONDITIONS		
Fatigue		
HEPATOBILIARY DISORDERS		
		Increased ALT
		Increased AST
INVESTIGATIONS		
	Increased lipase	Weight decreased
	Increased amylase	
METABOLISM AND NUTRITION DISORDERS		
Anorexia	Hypokalemia	
MUSCULOSKELETAL AND CONNECTIVE TISSUE DISORDERS		
	Muscle spasms	
NERVOUS SYSTEM DISORDERS		

SWOG Authorized Document

Headache		
SKIN AND SUBCUTANEOUS TISSUE DISORDERS		
	Rash	
VASCULAR DISORDERS		
	Hypertension	Deep vein thrombosis
		Pulmonary embolism

Adverse events occurring in < 1% and/or case reports: Pain in extremity, rhinorrhea, nasopharyngitis, cough, dizziness, conjunctival hyperemia, sinus congestion, sweating increased, back pain, anxiety, mild somnolence, palpitations, increased heart rate, increased blood luteinizing hormone, increased cortisol-free urine, staphylococcal skin infection, lymphopenia, adrenal insufficiency, dyspnea, thrombocytopenia, hyperthyroidism, reduction in ejection fraction, angina.

Animal Data: Administration of TAK-700 to dogs and monkeys was associated with decreased plasma cortisol concentration, increased adrenal weight, and microscopic findings, including depletion of lipids in the zona fasciculata and/or vacuolation of the adrenal cortex. There were no effects on blood pressure, heart rate, or any ECG changes.

2. Pregnancy and Lactation: Administration of TAK-700 led to maternal and fetal toxicity in rats and rabbits, including placental abnormalities, increased placental weight, increased fetal mortality, decreased skeletal ossification, and decreased fetal weight. TAK-700 should not be given to pregnant women. Females of childbearing potential should only receive TAK-700 if they are using adequate methods of contraception. Males should use effective methods of contraception through defined periods during and after treatment with TAK-700. It is not known if tak-700 is excreted in human breast milk; therefore, TAK-700 should not be administered to women who are nursing.

3. Drug Interactions: TAK-700 is a moderate inducer of CYP1A2 and has the potential to induce the metabolism of drugs that are substrates of CYP1A2. TAK-700 inhibits CYP1A2, CYP2C8, CYP2C9, and CYP2C19. However, based on the results of CYP isozyme and transporter inhibition studies, orteronel is unlikely to cause CYP isozyme- or transporter-mediated drug-drug interactions (DDI). Overall, the risk of DDI with TAK-700 is low.

4. Other

A degradant in the TAK-700 tablet tested positive for genotoxicity in *in vitro* tests and at high dose *in vivo* and could potentially pose a risk to human subjects by leading to additional malignancy. The potential clinical implications of these in vitro assay results are that any resulting genetic or chromosomal change could potentially lead to secondary malignancy and may pose a risk to an unborn child. However, an adequate safety margin was demonstrated *in vivo* at approximately seven times the exposure in humans administered a dose of orteronel 400 mg twice daily.

d. DOSING & ADMINISTRATION

Dosing – See Section 7.0 Treatment Plan

TAK-700 will be given orally twice daily (BID) throughout each treatment cycle of the study, except for dose modifications. TAK-700 tablets should be swallowed

SWOG Authorized Document

whole as there is insufficient information regarding absorption if crushed, chewed, or dissolved in liquid.

Study drug should be taken BID at the same time each day, approximately 12 hours apart, and may be taken with or without food. Missed doses of study drug may be taken later, provided that the dosing interval is at least six hours before the next dose, otherwise missed doses should be omitted. Note: Patients who experience Grade 1 or 2 nausea or vomiting may be advised to take study drug with or following meals.

e. HOW SUPPLIED

1. TAK-700 (orteronel) is investigational for this study and will be supplied free of charge to patients by Millennium Pharmaceuticals, Inc. for distribution by Biologics.

2. TAK-700 (orteronel) is a white, nonhygroscopic, crystalline powder. Tablets are provided as round (100 mg) pale red, IR film-coated tablets for oral administration.

   The following excipients may be present in the 100 mg and 200 mg film-coated tablets: mannitol, microcrystalline cellulose, sodium starch glycolate, hydroxypropyl cellulose, magnesium stearate, hypromellose 2910, polyethylene glycol (8000), titanium dioxide, ferric oxide (red), and purified water.

f. STORAGE, PREPARATION & STABILITY

TAK-700 (orteronel) l film-coated tablets should be stored in their original packaging at 20°C to 25°C (68°F to 77°F), with excursions permitted from 15°C to 30°C (59°F to 86°F). Orteronel tablets must not be used past the stated retest or expiry date provided by Millennium.

Sites and subjects are advised to keep orteronel in its original container when feasible. If container must be opened, repackage in HDPE bottles and dispense a quantity sufficient to meet clinical supply needs in accordance with amount prescribed. Store the remaining product in its original container. Labeling should be done in accordance to the law and contain the statement "CAUTION: NEW DRUG – LIMITED BY FEDERAL LAW TO INVESTIGATIONAL USE."

g. DRUG ORDERING AND ACCOUNTABILITY

1. Drug Ordering: TAK-700 (orteronel) may be requested by the Principal Investigator (or their authorized designee) at each participating institution by completing and faxing the Biologics Drug Request for **<u>S1216</u>** (see **<u>S1216</u>** abstract page on the SWOG website) once patient has been registered. **Allow 7 business days for shipment of drug from receipt of the Biologics Drug Request Form. Form should be faxed to the number on the form**. The TAK-700 (Orteronel) to be supplied for this protocol is intended for clinical trial use only.

   <u>Initial Shipments</u>: Once a patient is registered, the site will fax a completed Drug Request Form to Biologics at 919/256-0794. Upon receipt of completed and faxed Drug Request Form, Biologics Inc. will:

   - Check to confirm site has an active site registration

   - Place a call or email to the site confirming the Drug Request Form was received, while providing the estimated day and time of arrival for the study drug.

SWOG Authorized Document

- Prepare an initial shipment of study drug per protocol below:

<u>Biologics Dispensing Schedule</u>

**'Drug Request Form' Received**	**Month 3**	**Month 6**	**Month 9**
Call Site Ship initial 3 month supply	Call Site for follow-up shipment Ship month 4-6 supply	Call Site for follow-up shipment Ship month 7-9 supply	Call Site for follow-up shipment Ship month 10-12 supply
**Month 12**	**Month 15**	**Every 3 Months**	
Call Site for follow-up shipment Ship month 13-15 supply	Call Site for follow-up shipment Ship month 16-18 supply		

- Each shipment of study drug contains:
  -9 bottles of TAK-700 (Orteronel): qty 64 tablets/bottle; 100 mg tablet dose
  -3 month supply at 300 mg (3 tablets/100 mg each) PO BID

- All study drugs will be shipped in original manufacturers packaging with a patient specific label adhered to the outer packaging. Biologics will place manufacturers packaging in a Ziploc bag.

- Each shipment includes a patient label on the Ziploc bags with the following information:
  - The Study Number (i.e. SWOG **<u>S1216</u>**);
  - IND caution statement and/or local regulatory statements;
  - Drug Identification (TAK-700)
  - Lot # and Expiration date
  - Storage conditions
  - Dosing Instructions (i.e." Administer as directed per protocol SWOG **<u>S1216</u>**")
  - Subject ID number (e.g. S1216-YYY, where the study number and patient ID number represents the unique patient identifier assigned by SWOG at registration);
  - The patient's initials (i.e. First, Middle, Last);
  - Storage Instructions (i.e. "Store at controlled room temperature, 15-30°C (59-86°F)");
  - Emergency contact instructions

<u>Subsequent Shipments</u>: Biologics will contact the site with a follow-up call approximately one month prior to the next scheduled shipment to confirm the patient is remaining on therapy and to arrange the date/time of delivery.

For all shipments from Biologics:

- A complete accountability record including date of dispense, site name, quantity dispensed, and balance forward will be recorded.

SWOG Authorized Document

Study accountability records are documented in electronic 21 CFR format and are kept in a secured area for duration of the study.

- A pharmacy review is provided; a licensed pharmacist checks off package for accuracy of contents, authorizing order via 21 CFR compliant trial accountability log.

- Biologics will enclose a Delivery Ticket that includes the quantity of drug provided with a section to be completed once received by the site coordinator. This section includes confirmation of drug receipt, verification of package contents, and instruction to fax the completed packing slip to Biologics.

- Biologics will process and ship authorized and completed orders "same day" of receipt if received before 2:00 p.m. EST Monday through Friday. Authorized and completed orders received after 2:00 pm EST Monday through Friday will be processed and shipped the next business morning. Biologics will be closed the following holidays: New Year's Day, Memorial Day, Independence Day, Labor Day, Thanksgiving, Thanksgiving Friday, Christmas Eve, and Christmas Day.

- Packages are tracked until confirmed delivered and delivery exceptions are managed with the highest level of urgency to ensure therapy start date adherence. Packing slips with the shipment tracking number included will be faxed to the designated site coordinator for all shipments.

  <u>Order confirmation</u>: Once study drug is received at the clinical trial site, the designated site coordinator validates contents of package matches information provided on packing slip, signs off on the packing slip, and faxes completed form to Biologics to validate shipment has been received and is accurate.

2. Drug Handling and Accountability

   The investigator, or a responsible party designated by the investigator, must maintain a careful record of the receipt, disposition, and return or disposal of all drugs received from the supplier using the NCI Drug Accountability Record Form (DARF) for Oral Agents available at http://ctep.cancer.gov.

3. Drug Returns

   Unused drug supplies should NOT be returned. Unused drug should be disposed of per local institutional guidelines and documentation of destruction noted on the NCI Drug Accountability Record Form for Oral Agents as noted above.

4. Contact Information

   Questions about drug orders, transfers, returns, or accountability should be addressed to Clinical Research Services at Biologics, Inc. (800/693-4906 or clinicalresearchservices@biologicsinc.com).

SWOG Authorized Document

4.0 STAGING CRITERIA

This section is not applicable to this study.

5.0 ELIGIBILITY CRITERIA

Each of the criteria in the following section must be met in order for a patient to be considered eligible for registration. Use the spaces provided to confirm a patient's eligibility. For each criterion requiring test results and dates, please record this information on the **S1216** Onstudy Form and submit via Medidata Rave® (see Section 14.0). Any potential eligibility issues should be addressed to the Data Operations Center in Seattle at 206/652-2267 prior to registration.

In calculating days of tests and measurements, the day a test or measurement is done is considered Day 0. Therefore, if a test is done on a Monday, the Monday 2 weeks later would be considered Day 14. This allows for efficient patient scheduling without exceeding the guidelines. **If Day 14, 28, 30, 42, 56 or 90 falls on a weekend or holiday, the limit may be extended to the next working day.**

There are two patient populations eligible for the study: those who have not started any therapy with LHRH agonist or antagonist (or orchiectomy) (Early Induction Group) and those who have already started therapy with LHRH agonist or antagonist (or orchiectomy) within the 30 days prior to registration (Late Induction Group). Patients must be registered within 30 days of first injection of the LHRH agonist or antagonist (or orchiectomy).

5.1 Disease Related Criteria

a. All patients must have a histologically or cytologically proven diagnosis of adenocarcinoma of the prostate. All patients must have metastatic disease as evidenced by soft tissue and/or bony metastases prior to initiation of androgen deprivation therapy.

b. Patients who have not yet started androgen deprivation therapy (LHRH agonist/antagonist or orchiectomy) and will not have an LHRH agonist injection until after randomization (early induction group) must have radiographic assessments of all disease including bone scan (or PET scan) within 42 days prior to registration. In a late induction patient, if any of the required scans have not been obtained prior to starting LHRH agonist/antagonist or orchiectomy, those scans will need to be obtained prior to registration. All disease must be assessed and documented on the Baseline Tumor Assessment Form. NOTE: Androgen deprivation therapy does not include treatment with anti-androgens such as bicalutamide or flutamide or five alpha reductase inhibitors such as finasteride or dutasteride.

c. Patients with known brain metastases are not eligible. Brain imaging studies are not required for eligibility if the patient has no neurologic signs or symptoms suggestive of brain metastasis. But, if brain imaging studies are performed, they must be negative for disease.

d. Patients who are deemed to have high-risk or extensive metastatic, hormone sensitive prostate cancer (mHSPC) per “clinical judgment” of the treating physician are eligible for enrollment if they are unsuitable candidates for docetaxel or if they have declined docetaxel therapy.

5.2 Prior Therapy Criteria

a. Patients may have received prior androgen deprivation therapy (ADT) - neoadjuvant and/or adjuvant setting only - but it must not have lasted for more

SWOG Authorized Document

than 36 months (note that this is NOT the same as "late induction" as described in Section 5.1b above). Single or combination therapy allowed. At least 6 months must have elapsed since completion of androgen deprivation therapy in the neoadjuvant and/or adjuvant setting, and serum testosterone must be > 50 ng/dL (non-castrate levels) within 28 days prior to registration for early induction patients. Note: Serum testosterone assessment is required for eligibility for only those early induction patients with prior treatment with neoadjuvant or adjuvant ADT. Late induction patients with prior neoadjuvant or adjuvant ADT do not need serum testosterone assessment.

b. Patients must not have received prior and/or must not have any plans for receiving concomitant therapy with ketoconazole, aminoglutethimide, or abiraterone acetate, or enzalutamide (MDV3100). Concurrent megestrol for hot flashes is allowed.

c. Patients must not have received any prior cytotoxic chemotherapy for metastatic prostate cancer. Prior cytotoxic chemotherapy with curative intent in the neoadjuvant or adjuvant setting is allowed. At least 2 years must have elapsed since completion of cytotoxic chemotherapy in the neoadjuvant and/or adjuvant setting.

d. Patients may have received prior surgery. For all major surgeries, at least 14 days must have elapsed since completion and patient must have recovered from all major side effects of surgery per investigator's assessment.

e. Patients may have received or plan to receive concurrent bone targeting agents that do not have an effect on PSA (e.g. denosumab or bisphosphonate).

f. Patients must have no plans to receive any other experimental therapy while on the protocol treatment. Previous experimental therapy must have been completed at least 28 days prior to registration.

g. In the late induction group, patients must have had no more than 30 days of prior castration (medical or surgical) for metastatic prostate cancer prior to registration. The start date of medical castration is considered the day the patient first received an injection of a LHRH agonist/antagonist (or orchiectomy), not an oral antiandrogen.

   If the method of castration was luteinizing hormone releasing hormone (LHRH) agonists (i.e., leuprolide or goserelin), the patient must be willing to continue the use of LHRH agonist and add bicalutamide or TAK-700 (according to randomization) during protocol treatment.

   If the patient was on an antiandrogen (e.g. bicalutamide, flutamide), the patient must be willing to switch over to bicalutamide or TAK-700 (according to randomization). There is no limit on how many days a patient may have been on an antiandrogen (e.g. bicalutamide, flutamide) or a five alpha reductase inhibitor (e.g. finasteride or dutasteride) prior to going on study and no washout is required.

   If the method of castration was LHRH antagonists (i.e. Degarelix), the patient must be willing to switch to an LHRH agonist during protocol treatment.

5.3 Clinical/Laboratory Criteria

a. Patients must have a complete physical examination and medical history within 28 days prior to registration.

SWOG Authorized Document

b. Patients must have a PSA ≥ 2 ng/mL obtained within 90 days prior to registration.

c. A DEXA scan must be obtained within 2 years prior to registration.

d. Patients must not have New York Heart Association Class III or IV heart failure (see Section 18.4) at the time of screening. Patients must not have any thromboembolic event, unstable angina pectoris, myocardial infarction, or serious uncontrolled cardiac arrhythmia within 6 months prior to registration. (Note: Androgen deprivation therapy may prolong the QT/QTc interval. Patients with congenital long AT syndrome, congestive heart failure, frequent electrolyte abnormalities, and patients taking drugs known to prolong the QT interval may be at increased risk.)

e. Patients must have a QTc interval < 461 msec on the 12 lead ECG within 42 days prior to registration. Patients with asymptomatic or incidental bundle branch blocks may have QTc measured by a cardiologist or standard formulas such as Bazett's or Fridericia's to adjust for pre-existing blocks.

f. Patients must have a left ventricular ejection fraction (LVEF) ≥ 50% by echocardiogram or multiple gated acquisition (MUGA) scan within 42 days prior to registration.

g. Patients must have blood pressure measured within 28 days prior to registration. Patients must not have uncontrolled hypertension (defined as blood pressure > 160 mmHg systolic and > 90 mmHg diastolic at 2 separate measurements no more than 60 minutes apart) despite appropriate medical therapy. Note: Patients may be rescreened after adjustments of antihypertensive medications. See ACCF/AHA.AMA-PCPI joint statement.

h. Patients must have adequate hepatic function as evidenced by bilirubin ≤ 2 x institutional upper limit of normal (ULN), SGOT (AST) and SGPT (ALT) ≤ 3 x institutional ULN, or ≤ 5 x institutional ULN if liver metastases are present. These results must be obtained within 28 days prior to registration.

i. Patients must have adequate renal function as evidenced by calculated creatinine clearance ≥ 40 mL/min using a serum creatinine or by 24-hour urine creatinine obtained within 28 days prior to registration.

$$\text{Calculated creatinine clearance} = \frac{(140 - \text{age}) \times \text{wt (kg)}}{72 \times \text{creatinine (mg/dl)}}$$

j. Patients must have adequate hematologic function as evidenced by leukocytes ≥ 3,000/mcL, absolute neutrophil count (ANC) ≥ 1,500/mcL, hemoglobin ≥ 9 g/dL, and platelets ≥ 100,000/mcL. These results must be obtained within 28 days prior to registration.

k. Patients must not be known to have human immunodeficiency virus (HIV) infection, active chronic hepatitis B or C, life-threatening illness unrelated to cancer, or any serious medical or psychiatric illness that could, in the investigator's opinion, potentially interfere with participation in this study. Patients will be tested for hepatitis B or C or HIV infection during screening if they are considered by the investigator to be at higher risk for these infections and have not been previously tested.

l. Patients with a known history of primary and secondary adrenal insufficiency are not eligible.

SWOG Authorized Document

m. Patients must not be known to have hypersensitivity to TAK-700, to TAK-700 metabolites, to bicalutamide, or to LHRH agonist.

n. Patients must not have known gastrointestinal (GI) disease or GI procedure that could interfere with the GI absorption or tolerance of TAK-700, including difficulty swallowing oral medications per investigator's clinical judgement.

o. Patients must have a Zubrod performance status of 0 - 2 (see Section 10.4). Zubrod performance status 3 will be allowed if from bone pain only.

p. No other prior malignancy is allowed except for the following: adequately treated basal cell or squamous cell skin cancer, adequately treated Stage I or II cancer from which the patient is currently in complete remission, or any other cancer from which the patient has been disease-free for 5 years.

q. Patients must be ≥ 18 years of age.

r. Men of reproductive potential and those who are surgically sterilized (i.e., post-vasectomy) must agree to practice effective barrier contraception or agree to abstain from intercourse while receiving treatment on this study and for at least 4 months after protocol treatment ends.

5.4 Specimen Submission Criteria

a. Patients must be offered the opportunity to participate in specimen banking for future use to include translational medicine studies oulined in Section 15.0.

5.5 Regulatory Criteria

a. Patients or their legally authorized representative must be informed of the investigational nature of this study and must sign and give written informed consent in accordance with institutional and federal guidelines.

Voluntary written informed consent must be obtained before performance of any study-related procedure not part of normal medical care, with the understanding that consent may be withdrawn by the patient at any time without prejudice to future medical care.

b. As a part of the OPEN registration process (see Section 13.4 for OPEN access instructions), the treating institution's identity is provided in order to ensure that the current (within 365 days) date of institutional review board approval for this study has been entered in the system.

6.0 STRATIFICATION FACTORS

Patients will be randomized using a dynamic balancing algorithm *(36)* with stratification based on:

- Severity of Disease: extensive vs. minimal (as assessed at the diagnosis of metastatic cancer of the prostate).
  - Minimal: Patients with involvement of vertebrae and/or pelvic bones and/or lymph nodes.
  - Extensive: All patients with greater than minimal involvement.
- Zubrod Performance Status (0-1 vs 2-3), NOTE: If Performance Status is 3; it is due to bone pain only.
- Pre-registration treatment status: early induction vs. late induction

SWOG Authorized Document

- Early induction: patients who have not started any therapy with LHRH agonist or antagonist (or orchiectomy).
- Late induction: patients who have already started therapy with LHRH agonist or antagonist (or orchiectomy) within 30 days prior to registration.

7.0 TREATMENT PLAN

For treatment or dose modification related questions, please contact Dr. Neeraj Agarwal (E-mail: neeraj.agarwal@hci.utah.edu or Phone 801/587-4765), Dr. Maha H.A. Hussain (E-mail: mahahuss@med.umich.edu or Phone 734/936-8906) or Dr. Shilpa Gupta (E-mail: guptash@umn.edu or Phone 860/997-2918). For dosing principles or questions, please consult the SWOG Policy #38 "Dosing Principles for Patients on Clinical Trials" at http://swog.org (then click on "Policies and Manuals" under the "Visitors" menu and choose Policy #38).

7.1 Treatment

Patients who are not surgically castrated will be randomized to either Arm 1: LHRH agonist + TAK-700 or Arm 2: LHRH agonist + bicalutamide. Patients who are surgically castrated will receive only TAK-700 (Arm 1) or bicalutamide (Arm 2). Any LHRH agonist (e.g., leuprolide acetate or goserelin acetate) is acceptable. Patients will receive treatment until progression or other reason for removal from protocol treatment (see Section 7.4). However, patients off bicalutamide or TAK-700 but continuing on LHRH agonist will remain on protocol treatment (see Section 7.4b). If new bone lesions appear in the absence of PSA rising within the first year of protocol treatment, the Study Chair must be consulted to discuss the possibility of tumor flare before taking patient off protocol treatment.

It is suggested that, for early induction patients, at the discretion of the treating investigator, patients can start bicalutamide or TAK-700 within 7 days prior to starting LHRH agonist to prevent a clinical flare reaction. Patients on other anti-androgens prior to registration must switch to bicalutamide or TAK-700 (depending upon randomization). No washout is required for switching from prior anti-androgens to bicalutamide or TAK-700 after randomization.

a. Treatment schedule Arm 1:

AGENT	DOSE	ROUTE	DAYS
For non-surgical castration patients only:			
LHRH Agonist	*	IM or SubQ*	
TAK-700	300 mg	PO	Twice Daily

* NOTE: LHRH Agonist will be given as approved for androgen deprivation at a dose necessary to maintain castrate levels and equivalent to 22.5 mg of Leuprolide IM every 3 months.

b. Treatment schedule Arm 2:

AGENT	DOSE	ROUTE	DAYS
For non-surgical castration patients only:			
LHRH Agonist	*	IM or SubQ*	
Bicalutamide	50 mg	PO	QD

* NOTE: LHRH Agonist will be given as approved for androgen deprivation at a dose necessary to maintain castrate levels and equivalent to 22.5 mg of Leuprolide IM every 3 months, e.g., leuprolide 45 mg IM every 6 months is equivalent to leuprolide 22.5 mg IM every 3 months.

SWOG Authorized Document

Please note that for late induction patients, the subsequent dose of leuprolide may be delayed by a few weeks, per treating investigator's discretion to accommodate patient scheduling.

PSA values will be assessed prior to treatment, every month for the first 4 months, and then every 3 months while on protocol treatment. More frequent PSA monitoring is acceptable, but all measures should be reported on the **S1216** Prostate Specific Antigen Reporting Form.

7.2 Concomitant Therapy

a. The use of supportive care medications is allowed according to institutional standards.

b. **Several medications are specifically disallowed. Five-alpha reductase inhibitor (e.g. finasteride and dutasteride) are not permitted. Other excluded therapies include ketoconazole, aminoglutethimide, abiraterone acetate, enzalutamide (MDV3100), diethylstilbestrol/DES, and other estrogen preparations.**

c. Radiotherapy: Radiation therapy will be allowed ONLY for baseline symptoms (e.g. primary prostate related urinary symptoms and bone pain), per investigator's clinical judgment during the first four months of protocol treatment.

d. Orchiectomy: Surgical castration may occur prior to randomization or while on study.

e. Androgen deprivation therapy may prolong the QT/QTc interval. Patients taking drugs known to prolong the QT interval may be at increased risk.

7.3 Drug Compliance Documentation

Bicalutamide or TAK-700 drug compliance will be recorded on a treatment Intake Calendar (see Section 18.1). Institutional CRAs will review and ascertain patient adherence with protocol therapy at the end of treatment for each cycle. Calendar should be kept in the patient's clinic chart. Note that the Intake Calendar is provided only as a tool for tracking compliance. Sites may use institutional pill diaries or other source documentation in place of Intake Calendar at the discretion of the treating physician.

7.4 Criteria for Removal from Protocol Treatment

a. Progression of disease or symptomatic deterioration (as defined in Section 10.3).

b. Unacceptable toxicity due to LHRH agonist. NOTE: Patients off bicalutamide or TAK-700 due to toxicities, but continuing on LHRH agonist remain on protocol treatment.

c. Delay in LHRH agonist greater than 60 days.

d. Beginning a new systemic treatment for prostate cancer.

e. The patient may withdraw from the study at any time for any reason.

7.5 Discontinuation of Treatment

All reasons for discontinuation of treatment must be documented in the Off Treatment Notice.

SWOG Authorized Document

7.6 Follow-Up Period

All patients will be followed until death or 10 years after registration, whichever occurs first.

8.0 DOSAGE MODIFICATIONS

8.1 NCI Common Terminology Criteria for Adverse Events

This study will utilize the CTCAE (NCI Common Terminology Criteria for Adverse Events) Version 4.0 for toxicity and Serious Adverse Event reporting. A copy of the CTCAE Version 4.0 can be downloaded from the CTEP home page (http://ctep.cancer.gov). All appropriate treatment areas should have access to a copy of the CTCAE Version 4.0.

8.2 General Dose Modification Considerations

a. Missed doses of bicalutamide are to be omitted rather than made up. Missed doses of TAK-700 may be taken later, provided that the dosing interval is at least six hours before the next dose, otherwise missed doses should be omitted rather than made up.

b. If multiple toxicities are experienced, dose modifications will be based on the toxicity requiring the largest dose reduction.

c. Reductions are based on the dose given in the preceding cycle and are based on toxicities observed since the prior toxicity evaluation.

d. Dose modifications are required for Grade 3 or 4 adverse events (AEs) or intolerable Grade 2 AEs that are considered at least possibly related to TAK-700.

e. Toxicities will be assessed and reported monthly for the first 4 months and then every 3 months while on protocol treatment.

f. Electrolyte abnormalities should be corrected. Consider periodic monitoring of electrocardiograms and electrolytes.

8.3 Dose Levels of TAK-700 (Orteronel)

Dose Levels	Dose
Full	300 mg bid
-1 Level	200 mg bid
-2 Level	100 mg bid
-3 Level	Hold

8.4 Bicalutamide Dose Modifications

a. There are no dosage adjustments for hematologic toxicity. The need for the use of G-CSF is not anticipated.

SWOG Authorized Document

b. Dosage adjustments for Non-hematologic Toxicity:

1. Diarrhea:

The major toxic effect of bicalutamide is moderate diarrhea which is rarely severe (exclusion of other causes of diarrhea should be considered in severe cases). The dose modifications for this will be as follows.

- Grade 2 diarrhea, treat symptomatically with anti-diarrhea drugs.
- Grade 2 diarrhea unresponsive to symptomatic treatment or Grade 3 or 4 diarrhea - discontinue bicalutamide until diarrhea resolves ≤ Grade 1.

Further tests are up to discretion of treating physician. Restart treatment as per protocol when diarrhea resolves to ≤ Grade 1.

2. Abnormal Liver Function Tests (SGOT/AST, SGPT/ALT, Bilirubin)

Grade 2 or greater toxicity - Stop bicalutamide; wait until LFTs are normal (Grade 0). Hepatitis screening (A, B, C) should be done in all cases of abnormal LFTs which could be consistent with infectious hepatitis. To assess return to normal, abnormal liver function tests should be done weekly or as per the discretion of the treating clinician. Restart treatment when LFTs return to normal. If toxicity (Grade 2 or worse) occurs again, discontinue bicalutamide. If bicalutamide is held for > 60 days, it should be permanently discontinued.

3. Patients may complain of flatulence, bloating, and mild "gas pains" which should not result in changes in treatment. Symptomatic treatment should be employed with antacids, simethicone, etc.

4. For any other Grade 3-4 adverse events at least possibly related to bicalutamide, stop bicalutamide; wait until adverse event has resolved to ≤ Grade 1, then restart bicalutamide.

*Asymptomatic Grade 3 or 4 laboratory findings, which are considered either clinically significant or not, may not require dose hold. The decision to hold the dose should be based on the investigator’s clinical judgment.

5. Criteria for Discontinuation of Bicalutamide

If for any reason, bicalutamide has been held for > 60 days, bicalutamide must be permanently discontinued, but patient remains on protocol treatment just receiving LHRH agonist until progression or other reason for removal from protocol treatment (see Section 7.4).

8.5 TAK-700 (Orteronel) Dose Modifications

The following instructions are for adverse events that are at least possibly related to TAK-700:

Grade 1 adverse events do not require a TAK-700 dose reduction.

For Grade 2 adverse events, a dose reduction of one level should be considered ONLY when the event is judged by the treating investigator to be clinically intolerable.

For Grade 3 and 4 adverse events, the dose modification of TAK-700 should follow the "Dose Reduction and Re-escalation guidelines" (see Sections 8.5a and 8.5b, respectively). For event of adrenal insufficiency, please refer to the specific guidelines on the treatment of suspected or possible adrenal insufficiency. For the event of hypertension, please refer to the specific guidelines on the treatment of hypertension.

a. **Criteria For TAK-700 Initial Dose Reduction Dose Modifications For Grade 2** (intolerable only), Grade 3 Or Grade 4 Events

* Asymptomatic Grade 3 or 4 adverse events/laboratory findings NOT considered to be related to TAK-700 (e.g., Grade 3 anemia causing fatigue), irrespective of clinical significance may not require dose modification (i.e., dose hold or reduction). The decision should be based on the investigator's clinical judgment.

* Symptomatic Grade 3 or 4 adverse events/laboratory findings that are considered at least possibly related to TAK-700 require a dosing hold for a minimum of 2 weeks (Level -3, dose hold).

* For a clinically intolerable Grade 2 adverse event that is considered at least possibly related to TAK-700, the dose should be decreased by 1 dose level for 2 weeks, or even be held for 2 weeks per clinical judgement of the treating clinician. If the dose was decreased and the AE remains at Grade 2 and is still intolerable after 2 weeks, decrease the dose by another level. **The dose of TAK-700 may be decreased sooner or later than 2 weeks or even be held, per the clinical judgement of the treating clinician.**

  The investigator should identify other potential causes of any adverse event or laboratory abnormality. Once the dose is reduced, the frequency of reassessment should be based on the clinical judgement of treating clinician.

NOTE: During episodes of acute illnesses (such as influenza), planned surgeries (such as a knee replacement surgery), unplanned surgeries (such as cholecystectomy) or hospitalizations (such as a fracture following a fall), NOT considered related to TAK-700, TAK-700 may be temporarily stopped for the duration of the illness or longer per investigator's clinical judgment. After resolution or improvement of this illness/episode, TAK-700 should be restarted at the dose on which the patient was on prior to this illness/surgeries/hospitalization.

b. **Criteria for TAK-700 Dose Re-Escalation**

1. Re-escalation for Grade 2 Intolerable Adverse Events

Re-escalation of TAK-700 after resolution or improvement of a Grade 2 intolerable AE will follow the criteria below:

a. If the AE grade improves to Grade 0, 1, or 2 tolerable AE, re-escalate the dose by 1 level.

b. If the AE remains at Grade 2 and is still intolerable after 2 weeks, decrease the dose by another level. If the dose was held, please continue to hold.

c. If the event worsens to Grade ≥ 3, hold TAK-700 for 2 weeks followed by a reassessment. Follow the re-escalation guidelines for Grade 3 or 4 adverse events as described in Section 8.5b.

Note: The above refers to adverse events considered at least possibly related to TAK-700. Reassess the AE after 2 weeks or sooner if the AE

SWOG Authorized Document

worsens. Continue to reassess periodically (per investigator's clinical judgement) until the event is resolved or stabilized. Continue to adjust the TAK-700 dose until the dose is optimally titrated. **During dose escalation, if toxicities that are at least possibly related to TAK-700 recur, TAK-700 can be continued at Level -1 or Level -2 without full dose escalation per investigator's clinical judgment.**

2. Re-starting of TAK-700 Following a Grade 3 or 4 Adverse Event

Re-starting following a Grade 3 or 4 AE considered at least possibly related to TAK-700 will follow the criteria below:

a. If the AE grade improves to Grade 0, 1, or 2 tolerable AE, re-start TAK-700 at one dose level below the one at which the patient was on at the time of experiencing the Grade 3 or 4 AE.

b. If the AE is Grade 2 intolerable, Grade 3 or 4 after 2 weeks, hold dosing for another 2 weeks and reassess again after 2 weeks and continue to reassess after that per investigator's clinical judgment.

Note: The above refers to adverse events considered at least possibly related to TAK-700. Reassess the AE after 2 weeks or sooner if the AE worsens. Continue to reassess periodically (per investigator's clinical judgement) until the event is resolved or stabilized. Continue to adjust the TAK-700 dose until the dose is optimally titrated. **During dose escalation, if toxicities that are at least possibly related to TAK-700 recur, TAK-700 can be continued at Level -1 or Level -2 without full dose escalation per investigator's clinical judgment.**

c. **Criteria for Discontinuation of TAK-700**

If for any reason, TAK-700 has been held for > 60 days, it will be permanently discontinued, but patient can remain on protocol treatment just receiving LHRH agonist.

d. **Hypertension**

Decisions to hold, decrease and re-escalate TAK-700 are based on three factors. The first is whether the treating physician considers hypertension to be related to TAK-700. The second is to assess the grade of the hypertension. The third is persistence of blood pressure (BP) readings (and not an isolated BP reading).

Modification of TAK-700 dose should be performed only if hypertension is considered related to TAK-700. Grading of hypertension should be based on persistent BP readings (and **NOT** based on how many antihypertensive drugs patient is on, as mentioned in the CTCAE v4 grading guidelines). For example, if a patient is on ≥ 1 antihypertensive medication this would suggest a CTCAE v4 Grade 3 hypertension, however, if the BP reading is 120/87, it should be graded as Grade 1 for treatment decision and dose modification under this protocol. Finally, BP readings used to grade hypertension should be based on more than one BP reading taken at least one hour apart and not based on an isolated BP reading.

e. **Suspected or Possible Adrenal Insufficiency**

SWOG Authorized Document

If patients experience adrenal insufficiency, the adrenal insufficiency will have the more nonspecific manifestations of glucocorticoid insufficiency, rather than the more specific electrolyte abnormalities of mineralocorticoid insufficiency. Patients experiencing severe physiological stress (e.g., surgery, severe infection) should be carefully monitored for adrenal insufficiency. Concomitant medications may complicate the picture of adrenal insufficiency, in particular in patients who are on beta-blockers or diuretics. Concomitant illness such as infection might similarly trigger or worsen symptoms of otherwise mild adrenal insufficiency. Grading of adrenal insufficiency, the clinical manifestations associated with the specific grade, and suggested management options are provided in the table below. In all cases, actions should include a thorough review for other possible causes or contributors to the presenting symptoms (e.g., infection, anemia, or newly introduced concomitant medications).

Table: Criteria and Appropriate Actions for Adrenal Insufficiency

**Severity**	**Symptoms/Signs**	**Action Required/Study Drug Modifications**
Possible mild insufficiency	• Chronic Grade 1 or Grade 2 fatigue, anorexia<br>• < 5% weight loss<br>• BP normal, possible mild orthostatic hypotension<br>• Weight loss	• Review medications, check electrolytes, cortisol and ACTH concentrations<br>• Continue study drug as per protocol
Possible or probable moderate adrenal insufficiency, acute or chronic	• Grade 2 or 3 fatigue, anorexia, intermittent nausea and vomiting, orthostatic lightheadedness or weakness<br>• Weight loss<br>• Definite orthostatic hypotension or below baseline supine BP<br>• Possible hyponatremia	• Review medications, check electrolytes, cortisol, and ACTH concentrations<br>• Review medications, including compliance with concomitant steroids and new diuretic use<br>• Dose reduction follows Grade 3 dose reduction and dose re-escalation guidelines
Possible or probable severe chronic or acute adrenal insufficiency	• Grade 3 or 4 fatigue, definite anorexia, nausea and vomiting<br>• Severe orthostatic symptoms<br>• Prostration<br>• Nausea and/or vomiting<br>• Hypotension at rest and unable to stand due to orthostatic hypotension, possible hyponatremia	• Manage in acute care facility<br>• Administer IV hydrocortisone and electrolyte/volume replacement<br>• Review medications, check electrolytes, cortisol and ACTH concentrations<br>• Dose reduction follows Grade 3 dose reduction and dose re-escalation guidelines if a reversible precipitating cause cannot be identified and reversed

SWOG Authorized Document

8.6 Dose Modification Contacts

For treatment or dose modification related questions, please contact Dr. Neeraj Agarwal (E-mail: neeraj.agarwal@hci.utah.edu or Phone 801/587-4765) or Dr. Maha H.A. Hussain (E-mail: mahahuss@med.umich.edu or Phone 734/936-8906) or Dr. Shilpa Gupta (E-mail: guptash@umn.edu or Phone 860/997-2918).

8.7 Adverse Event Reporting

Toxicities (including suspected reactions) that meet the expedited reporting criteria as outlined in Section 16.0 of the protocol must be reported to the Operations Office, Study Chair, the NCI via CTEP-AERS, and to the IRB per local IRB requirements.

SWOG Authorized Document

9.0 STUDY CALENDAR

REQUIRED STUDIES	PRE STUDY	M 1	M 2	M 3	M 4	M 5	M 6	M 7	M 8	M 9	M 10	M 11	M 12	β M 13	π FU Prior to Prog	FU After Prog
**PHYSICAL**																
History and Physical Exam §	X		X	X	X			X			X			q 3 mo	X	X
Weight and Performance Status §	X		X	X	X			X			X			q 3 mo		
Blood Pressure	X♥		X	X	X			X			X			q 3 mo		
Disease Assessment ≠	X													q 12 mo	X	
Toxicity Notation §		X	X	X	X			X			X			q 3 mo		
**LABORATORY**																
PSA	X	X*	X	X	X			X			X			q 3 mo	X	X
Testosterone Θ	X															
Bilirubin	X	X*	X	X	X			X			X			q 3 mo		
SGOT/SGPT	X	X*	X	X	X			X			X			q 3 mo		
Serum Creatinine for Calculated Creatinine Clearance	X	X*	X	X	X			X			X			q 3 mo		
CBC, Differential, Platelets, Hemoglobin	X	X*	X	X	X			X			X			q 3 mo		
Total Alkaline Phosphatase	X	X*	X	X	X			X						q 6 mo		
Albumin	X	X*						X						q 6 mo		
Lipid Profile (HDL, LDL, Triglycerides, Cholesterol) Σ	X	X*												q 12 mo		
ACTH, Cortisol and Corticosterone (for TAK-700 Arm Only) λ		X														
Amylase & Lipase ϕ	X															
Serum Electrolytes					X¥			X¥			X¥			X¥		

Study Calendar continued on next page)
Click here for Footnotes.

SWOG Authorized Document

REQUIRED STUDIES	PRE STUDY	M 1	M 2	M 3	M 4	M 5	M 6	M 7	M 8	M 9	M 10	M 11	M 12	β M 13	π FU Prior to Prog	FU After Prog
**SAMPLES (BANKING)**																
Tissue block or 30 unstained slides	Xϖ															
Buffy coat	X															
Plasma	X							X								X
Peripheral whole blood	X															X
Androgens (Testosterone and dehydroepiandrosterone sulfate [DHEA-S])	X√							X√						X√		X√
**X-RAYS AND SCANS**																
CT or MRI of Abdomen & Pelvis ≠	X													q 12 mo	X	
Bone scan or PET scan ≠	X													q 12 mo	X	
Brain CT †	X															
DEXA ə	X														X ə	
ECG	X															
Echocardiogram or MUGA Δ	X															
**TREATMENT** (see Section 7.1)																
**ARM 1** £:																
LHRHa		X			X			X			X			X		
TAK-700 Ω		X	X	X	X	X	X	X	X	X	X	X	X	X		
**ARM 2** £:																
LHRHa		X			X			X			X			X		
Bicalutamide Ω		X	X	X	X	X	X	X	X	X	X	X	X	X		

NOTE: Forms submission guidelines may be found in Section 14.0.

Click here for Footnotes.

SWOG Authorized Document

FOOTNOTES

§ History/physical exam and weight/performance status will be done monthly until Month 4 and then every three months while on study. Toxicity notation will be done after each month of protocol treatment for Months 1-4. After Month 4, toxicity notation should be done after every third month of protocol treatment.
≠ A CT/MRI of the abdomen & pelvis AND a bone scan (or PET scan) are acceptable for disease assessment. Disease assessments will be done annually until progression (and more frequently if clinically indicated) by the same methods used at baseline.
Ω TAK-700 is to be taken twice daily. Bicalutamide is to be taken daily.
π Once off protocol treatment prior to disease progression, disease should be assessed for all patients in follow-up by physical exam, bone scan (or PET scan) & CT or MRI of abd/pelvis once a year per investigator's clinical judgment for up to 10 years after registration or until death. PSA should be assessed every 6 months for the first 2 years and annually until 10 years after registration or until death.
Σ Obtain at baseline and then every year if normal. If abnormal profile, to be obtained and treated per institutional guidelines. (Note: The lipid panel should be collected under fasting conditions.)
† See Section 5.1c.
♥ See Section 5.3g.
Ə DEXA scan must be obtained within 2 years prior to registration for all patients. Follow-up DEXA scans must be obtained every two years from baseline, only if the patient is not on bisphosphonate or RANK-L inhibitor.
ϕ Only to be repeated, per institutional guidelines, during protocol treatment (if there is clinical or imaging evidence of pancreatitis).
Θ Only required for early induction patients who have had neoadjuvant or adjuvant androgen deprivation therapy. See Section 5.2a.
β Protocol treatment and assessment parameters will continue at these intervals (including radiographic assessment annually and DEXA scans [see Ə] every two years) until off protocol treatment (see Section 7.4).
λ For cortisol/corticosterone, other locally available tests such as 11 Deoxycortisol or 18 OH Corticosterone may be substituted at the PIs discretion to rule out hypoadrenalism. Draw prior to starting TAK-700.
Δ An echocardiogram or MUGA must be performed at baseline (within 42 days prior to registration) for all patients.
£ Day 1 of treatment for patients on Arm 1 is the first day of treatment on TAK-700. Day 1 of treatment for patients on Arm 2 is the day of randomization. Patients who have had a prior orchiectomy will not receive LHRHa treatment.
* Repeat if prestudy labs were not done within 30 days of C1D1.
√ See Section 15.1. Collect serum for androgen testing at prestudy (prior to C1D1), Month 7, and Month 13 (or progression, whichever occurs first).**(Note: Effective September 23, 2014, androgen testing has been permanently closed to new patients.) If patient had serum drawn at baseline before September 23, 2014 continue with serum collection at designated timepoints.**
¥ Electrolytes (sodium, potassium, chloride, and CO_2 [carbon dioxide or bicarbonate]). Starting with Month 4, it is recommended that serum electrolytes be monitored every 3 months per the discretion of the treating physician.
ϖ Submit 30 slides from radical prostatectomy or 10-15 slides from biopsy (see Section 15.2a).

Allowable windows:

Allowable windows for scheduled procedures and assessments performed every 7-14 days is +/- 1 day, every 21 days is +/- 3 days, every 3-6 months is +/- 7days, and every year is +/- 14 days. The window is to be calculated from the scheduled date of the procedure/assessment.

SWOG Authorized Document

10.0 CRITERIA FOR EVALUATION AND ENDPOINT ANALYSIS

10.1 Measurability of Lesions

a. **Measurable disease**: Measurable disease is defined differently for lymph nodes compared with other disease and will be addressed in a separate section below.

1. Lesions that can be accurately measured in at least one dimension (longest diameter to be recorded) by ≥ 1.0 cm with CT or MRI scans. All tumor measurements must be recorded in decimal fractions of centimeters.

   The defined measurability of lesions on CT scan is based on the assumption that CT slice thickness is 0.5 cm or less. If CT scans have slice thickness greater than 0.5 cm, the minimum size for a measurable lesion should be twice the slice thickness.

2. Malignant lymph nodes are to be considered pathologically enlarged and measurable if it measures ≥ 1.5 cm in **SHORT AXIS** (greatest diameter perpendicular to the long axis of the lymph node) when assessed by scan (CT scan slice recommended being no greater than 0.5 cm).

b. **Non-measurable disease**: All other lesions (or sites of disease), including small lesions (longest diameter < 1.0 cm or pathologic lymph nodes with ≥ 1.0 cm to < 1.5 cm short axis), are considered non-measurable disease. Bone lesions, leptomeningeal disease, ascites, pleural/pericardial effusions, lymphangitis cutis/pulmonitis, inflammatory breast disease, and abdominal masses (not followed by CT or MRI), are considered non-measurable as are previously radiated lesions that have not progressed.

NOTE: **Patients who do not have measurable disease, but are clinically considered to have metastatic prostate cancer based on bone lesions or non-measurable, but pathologically enlarged lymph nodes, are eligible from this perspective.**

c. **Notes on measurability**

1. For CT and MRIs, the same type of scanner should be used and the image acquisition protocol should be followed as closely as possible to prior scans. Body scans should by performed with breath-hold scanning techniques, if possible.

2. PET-CT: At present, the low dose or attenuation correction CT portion of a PET-CT is not always of optimal diagnostic CT quality for use with RECIST measurements. However, if the site can document that the CT performed as part of a PET-CT is of identical diagnostic quality to a diagnostic CT, then the CT portion of the PET-CT can be used for RECIST measurements and can be used interchangeably with conventional CT.

3. Ultrasound: Ultrasound is not useful in assessment of lesion size and should not be used as a method of measurement.

SWOG Authorized Document

4. Cystic lesions that meet the criteria for radiographically defined simple cysts should not be considered as malignant lesions (neither measurable nor non-measurable) since they are, by definition simple cysts.

5. If a target lesion becomes very small some radiologists indicate that it is too small to measure. If the lesion is actually still present, a default measurement of 0.5 cm should be applied. If the radiologist believes the lesion has gone, a default measurement of 0.0cm should be recorded.

10.2 Progression Criteria

One or more of the following must occur:

1. ≥ 25% increase **AND** an absolute increase of at least 2 ng/mL from the nadir PSA (or from baseline PSA if there was no drop in PSA after starting treatment). If there is reasonable clinical suspicion of an aberrant PSA value, the rise in PSA may be confirmed at the discretion of the treating clinician, by a second PSA value obtained any time within the following 4 weeks to confirm PSA progression. The second PSA level should be consistent with PSA progression when compared to the nadir PSA. The date of progression must be recorded as the date of the first rise in PSA.

2. Progression on bone scan is defined as 2 or more new lesions on radionuclide bone scans. Should two or more new bone lesions be evident at the first assessment on treatment (Month 13), two or more additional new lesions must be evident on a confirmatory assessment at least 8 weeks or later (at investigator's discretion). This confirmation is not required when 2 or more new lesions first appear in subsequent assessment timepoints after the Month 13 assessment. The date of progression should be recorded as the date of the first bone scan that showed progression.

3. Soft tissue progression per RECIST 1.1 criteria. Twenty percent increase in the sum of appropriate diameters of target measurable lesions over smallest sum observed (over baseline if no decrease during therapy) using the same techniques as baseline, as well as an absolute increase of at least 0.5 cm. Unequivocal progression of non-measurable disease in the opinion of the treating physician (an explanation must be provided). Appearance of any new lesion/site.

4. Death due to disease without prior documentation of progression and with symptomatic deterioration (see Section 10.3).

Notes regarding new lesions: FDG-PET imaging can complement regular scans in identifying new lesions according to the following algorithm.

1. Negative FDG-PET at baseline, with a positive FDG-PET at follow-up is a sign of progression based on a new lesion.

2. No FDG-PET at baseline and a positive FDG-PET at follow-up corresponding to a potential new site of disease must have a confirmation by anatomical assessment (e.g. CT, MRI, x-ray, bone scan) as new site of disease to be considered progressive disease. In such a case, the date of progressive disease will be the date of the initial abnormal FDG-PET.

10.3 Symptomatic deterioration

Global deterioration of health status requiring discontinuation of treatment without objective evidence of progression. Efforts should be made to obtain objective evidence of progression after discontinuation.

SWOG Authorized Document

10.4 Performance Status

Patients will be graded according to the Zubrod Performance Status Scale.

**POINT**	**DESCRIPTION**
0	Fully active, able to carry on all pre-disease performance without restriction.
1	Restricted in physically strenuous activity but ambulatory and able to carry out work of a light or sedentary nature, e.g., light housework, office work.
2	Ambulatory and capable of self-care but unable to carry out any work activities; up and about more than 50% of waking hours.
3	Capable of limited self-care, confined to bed or chair more than 50% of waking hours.
4	Completely disabled; cannot carry on any self-care; totally confined to bed or chair.

10.5 Progression-Free Survival

From date of randomization to first occurrence of progression, symptomatic deterioration, or death due to any cause. Patients without progression are censored at last date of contact.

10.6 Time to Death

From date of registration to date of death due to any cause. Patients last known to be alive are censored at date of last contact.

10.7 PSA Response Categories

Undetectable PSA is defined as a PSA level of ≤ 0.2 ng/mL after six months, without evidence of progression or symptomatic deterioration, as defined in Section 10.3. A PSA partial response (PSA PR) after six to seven months is defined as a PSA that is between 0.2 and 4 ng/mL.

11.0 STATISTICAL CONSIDERATIONS

11.1 Accrual Goal

In 2014, results from the CHAARTED study were reported, showing significantly improved survival benefit with addition of frontline chemotherapy with docetaxel to androgen deprivation therapy (ADT) in men with high volume new mHSPC or those with new mHSPC with visceral metastasis. Since then, clinical practice has evolved accordingly. This change in practice has also resulted in preferential enrollment of men without visceral metastasis or low volume mHSPC in to the **S1216** trial. These patients are significantly more likely to have tumors driven predominantly by androgen signaling. Given this shift in the patient population of **S1216**, we anticipate that treatment effect of ADT + TAK-700 is going to be larger. Furthermore, the accrual on **S1216** has been steady and has been on target since it was fully activated in the year 2013. We plan to improve accrual further by several means (including webinars, personal communication with the top accruers, and increased awareness of the trial through patient advocacy). This is going to result in a trial with a smaller sample size and a shorter reporting period.

SWOG Authorized Document

We have increased the size of the expected treatment effect that would be of interest in this trial from a 25% increase in the median survival to a 33% increase. These design decisions were made prior to any formal interim analyses.

The accrual goal for this study is 1,186 eligible patients. **SWOG-9346** study, with similar eligibility requirement, was able to register 40 patients per month with intergroup participation. Given our experience with **SWOG-9346**, we are projecting accrual of 36 patients per month for this trial without EORTC participation. This study should take approximately 2.75 years to complete accrual, and then 3 additional years of follow-up. Assuming an ineligibility rate of approximately 10%, we will accrue 1,304 patients to achieve our goal of 1,186 eligible, randomized patients.

Summary of design amendment changes:

	Prior Design	Current Design
N (eligible/total)	1486/1636	1186/1304
Treatment Effect Size	HR=1.25	HR=1.33
Approximate Trial Duration	8.5 years	5.75 years
# of interim analysis	5 interim + final	3 interim + final

11.2 Analysis of Primary Endpoint

We assume that the median survival of those randomized to CAD is conservatively 54 months (48 months based on the median from **SWOG-9346** + an additional 6 months from newer downstream drugs on market which may extend survival).

We would be interested in the experimental regimen with TAK-700 if median survival were improved by 33% (i.e., median=72 mo). With 2.75 years of accrual and 3 more years of follow-up, and assuming a one-sided alpha=0.025 and 90% statistical power, we will need 1,186 eligible patients randomized to the two arms (translates to 36 patients/month accrual rate).

Three interim analyses are specified for this trial. Because deaths occur fairly slowly, and PFS (based on the PCWG2 definition) is moderately correlated with survival (R^2=0.34) in this patient population, we would like to base our early futility interim analyses on the intermediate endpoint of progression-free survival where progression is defined by PSA progression alone (per protocol) based on the modified Prostate Cancer Working Group 2 (PCWG2) definition rather than survival. *(37)* We use a hazard ratio = 1.25 for the null hypothesis of the PFS futility analysis because we expect there is not a 100% correlation between the magnitude of the PFS treatment effect and that for OS.

Goldman and colleagues found in their simulations under the alternative hypothesis, this futility testing of a moderately correlated intermediate endpoint resulted in negligible loss of power. *(38)* For the first interim analysis (timing as specified by the table below), one hypothesis test will be conducted. Evidence suggesting early termination of the trial and a conclusion that the TAK-700 arm is not better than the standard arm would be if the alternative hypothesis of a 25% improvement in PFS with the experimental arm is rejected at the one-sided 0.01 level. For the 2nd and 3rd interim analyses, the null and alternative hypotheses with respect to survival (and not PFS) will be tested at the one-sided 0.005 and 0.01 level respectively. The number of PFS endpoints is based on **SWOG-9346** data, where the standard CAD arm had a median PFS=36 months using PCWG2 criteria. *(39,40)*

SWOG Authorized Document

		# of expected deaths				Interim testing	
Interim analysis	Approx. time since start of trial	Standard arm	Exp arm	% of expected death information	PFS endpoints in the combined arms	Superiority endpt, Z-score	Futility endpt, Z-score
1	2.75 yrs end of accrual	107	88	38%	278	Not performed	PFS, 2.32
2	4 yrs (1.25 years after end of accrual)	190	158	67%		OS, 2.58	OS, 2.32
3	5 yrs (2.25 years after end of accrual)	246	207	86%		OS, 2.58	OS, 2.32
4	5.75 years (3 years after end of accrual)	283	240	100%		OS, 2.01	OS, 2.32

If the decision is to continue the study before reporting, the final analysis will be conducted when the required number of deaths have occurred in both arms or at a maximum of five years after accrual has completed, whichever comes first. A one-sided stratified logrank test will be used to test the primary survival hypothesis at the alpha = 0.022 level.

All analysis timing will be based on the specified number of events in both arms and the analyses will be stratified for factors specified in Section 6.0 of the protocol. For interim analysis #1, timing will be based on the number of PFS endpoints. For interim analyses #2 and #3 and the final analysis, timing will be based on the specified number of deaths.

11.3 Analysis of Secondary Endpoints

For the secondary endpoint of progression-free survival, we will assume that 50% of the patients on the standard arm will progression by 36 months. With the same study design specified in 11.1, the study will have 96% (84%) power to detect a 33% (25%) increase in median PFS (36 vs. 45 month median = 25%, 36 vs 48 month median = 33% relative improvement) using a one-sided logrank test with alpha=0.025 for the experimental vs. standard comparison.

For the comparison of PSA response by treatment arm, a patient's PSA response after 6 months of protocol hormonal therapy will be categorized as no response (> 4.0 ng/ml) vs. PSA response (0.3-4.0 ng/ml) vs. undetectable (≤ 0.2 ng/ml). These categories are based on prior clinical results published from **S9346** which indicated that these PSA response categories are strongly prognostic for subsequent survival. Polychotomous logistic regression will be used to model these three outcomes with adjustment for the stratification factor, and then the main effect of treatment arm will also be assessed to see if the PSA response pattern varied by treatment arm. In more exploratory analyses, classification and regression trees may be used to see if there are more optimal cutpoints for PSA than what is prespecified for identifying survival prognostic groups. Half of the data will be used as the test group and half will be used for validation. We will also evaluate whether there is a treatment interaction with PSA response, i.e., does the PSA response – survival association vary by treatment arm.

SWOG Authorized Document

Secondary analyses will be done for comparison between the two experimental arms regarding adverse events. With 593 eligible patients per arm, toxicity rates can be estimated to within at worst ± 4% (95% confidence interval).

11.4 Analysis of Translational Medicine (TM) Endpoints

a. Based on prior accrual experience with **SWOG-9346** (same patient population), it is anticipated that 28 patients per month will be randomized to this study on an intergroup-wide basis. An accrual period of 4.5 years will be required to complete a 2-arm study containing 743 patients in each arm, that is, a total of 1486 patients. We also anticipate that approximately anywhere from 10-40% of patients will have started hormonal therapy within the month prior to being randomized to this study.

It will be assumed that the control arm (CAD) will produce a median survival of 54 months. Assuming exponential survival, 4.5 years of patient accrual, 4 years of additional follow-up and a sample size of 1486 patients (743 patients per arm), the study has 90% power to detect a 25% increase in median survival (hazard ratio 1.25), using a one sided log rank test with alpha = 0.025 for the experimental/control comparison.

b. We anticipate that approximately 90% of patients will agree to participate in the longitudinal serum biologic studies (n = 670 subjects per arm) and will provide a study entry sample. This includes the subset of patients who have already started androgen deprivation therapy within the month prior to randomization. We will evaluate the distribution of pre-randomization marker levels among those who have and have not started CAD prior to randomization (stratification factor). If it appears that the prior initiation of CAD impacts the study entry bone marker levels to a fair extent then analyses stratified by CAD initiation status will be conducted. The following power calculations assume that the two strata can be pooled together for the analyses of interest.

A question of interest is whether baseline levels of bone markers are correlated with survival. If we assume no treatment by marker interaction and a two-sided alpha = 0.01 to account for the multiple marker testing, and in order to simplify the power calculations, if we split a bone marker such as N-telopeptide at the median value, then we will have 87% power to detect a survival hazard ratio of 1.32 (translates into a median survival of 3.9 years vs. 5.1 years) for those above relative to those below the median value of N-telopeptide. However, if we use quartiles or the continuous measures of each marker (transformed when appropriate), the power to detect associations will be greater. Landmark analyses and time-dependent proportional hazards regression models will be used to look at change in bone markers after seven months and subsequent survival. The appropriateness of the assumption of no treatment by bone marker interaction will be evaluated in the Cox model. Similar strategies will be used to evaluate the other bone markers. When interaction cannot be ruled out, prognostic factors will be evaluated separately within each treatment arm.

We will also evaluate the association of each marker with PSA status at 7 months (a secondary endpoint of the trial). We assume the overall undetectable PSA (≤ 0.2 ng/ml) response rate is 45% based on the results of **SWOG-9346**. With a sample size of 1340 and a two-sided alpha=0.01, there will be 80% power to detect an undetectable PSA response rate of 50% versus 40% for those below relative to those above the median for a particular serum bone marker.

SWOG Authorized Document

It may be that certain profiles of serum bone markers will respond more favorably to TAK-700 than others. In order to evaluate this initially, marker by treatment interactions will be placed in the proportional hazards regression model for survival and logistic regression for PSA response in addition to the main effects of treatment arm and bone marker measure. Classification and regression trees may also be used to investigate combinations of factors that identify prognostic groups.

The following is an example of the power that we will have for detecting treatment/marker interactions with survival as the endpoint. If we assume that for those with a marker below the median, the median survival for those on CAD and TAK-700 is 54 month, and we also assume 54 month median survival for those on the standard CAD arm with marker values above the median, then with two-sided alpha=0.01 we will have 85% power to detect a hazard ratio of 1.70 for standard CAD vs. TAK 700 among those with a marker above the median versus a hazard ratio of 1.0 for those below the median.

The table below illustrates the minimal effect sizes that can be detected for treatment-bone marker interactions with respect to undetectable PSA response assuming 80% power and a two-sided alpha=0.01.

**Detectable effect sizes[1] for treatment-bone marker interaction analyses (alpha=0.01 and 80% power) (assuming 603 responders, 737 non-responders based on SWOG-9346 PSA response data)**			
	**Odds ratio for bone marker below vs. above the median in the standard arm[2]**		
**Odds ratio for experimental treatment arm among those with bone marker above the median[3]**	***0.75***	***1.00***	***1.25***
**0.5**	2.15	2.16	2.19
**1.00**	2.15	2.17	2.20
**1.50**	2.21	2.24	2.27

[1] Detectable ratio of the bone marker-PSA response odds ratios between the experimental and standard treatment arm

[2] Effect (odds ratio) of a serum bone marker (below the median)

[3] Odds ratio for experimental vs. standard treatment arm for PSA response among those with a serum bone marker value above the median

c. Genomic Variants and Gene Expression of Androgen Pathway Genes

Aim 1: Association of germline (WBC) androgen pathway genes and clinical outcomes. Whole blood is already being stored on this recently activated trial. With a sample size target of 1500, we anticipate 80% (n=1200) will have adequate DNA based on prior SWOG trials. Variants will be tabulated and classified and allele frequencies calculated, stratified by race. The 95% confidence interval (CI) for the prevalence of any allele can be estimated within at least ± 3%. We will evaluate which variants are highly correlated and redundant with each other and are also associated (prevalence ≥3%) with 7-month PSA response and time to castration resistance (TTCR) as defined above.

We will randomly select 60% of the patients (n=720) for a training set and evaluate the univariate variant associations with PSA response and TTCR, and select those with a p-value < 0.05 for either endpoint. Because we will be validating our results and only using 60% of the data for training, we selected α=0.05 to give ourselves the chance of identifying relevant variants. We will then

explore multivariate model-building with these identified variants along with other covariates of age, race, bone pain status, extensive vs. minimal disease, and performance status. In order to identify combinations of variants that may work in an interactive way, we will employ an adaptive regression methodology for constructing predictors as Boolean combinations of binary covariates. *(41)* Because of the importance of model selection and wanting to avoid over-fitting the model, a LASSO approach will be used to estimate marginal effects by shrinking the model coefficients and thereby yield more stable results. Both logistic (PSA response) and proportional hazards (TTCR) models will be used. *(42)*

Validation of the univariate variant associations and multivariate selected model will be performed on the remaining 40% of the data (n=480). Associations at first will be assessed for clinical outcomes across both treatment arms, as explicit parameter estimates separated by treatment arm will not be reported until the primary trial results have been reported per the DSMC requirements. Variants with a prevalence of 3% or higher will be correlated with PSA response at 7 months defined as a value of 4 ng/ml or lower (Yes vs. No). We expect a PSA response rate of 69% at the end of 7 months of induction hormonal therapy based on the results of **S9346**, the previous SWOG hormonal therapy trial. *(43)* This can be further broken down as 26% normalized (0.2 ng/ml < PSA ≤ 4 ng/ml) and 43% undetectable (PSA ≤ 0.2 ng/ml). These finer PSA response categories will be explored in secondary analyses. There will be adequate power to detect odds ratios in the range of 1.6-2.4 when variant prevalence is 10% or higher in both the training and validation sets.

	Allele Frequency				
	3%	5%	10%	20%	30%
**(Training Set, n=720: 497 responders, 223 non-responders)**					
Odds ratio	3.25	2.56	2.02	1.72	1.61
PFS HR	2.30	1.92	1.61	1.43	1.37
**(Validation Set, n=480: 331 responders, 149 non-responders)**					
Odds ratio	4.20	3.10	2.40	1.94	1.81
PFS HR	2.81	2.23	1.79	1.56	1.48

Table 2. Minimally detectable odds ratios and HR for PFS for genetic variants and PSA response for the training and validation sets, assuming a two-sided α=0.05 and power=0.80 and median time to castration resistance of 36 months in those with the variant

In **S9346**, the median TTCR was 36 months. We assume one year of accrual will be completed when this grant starts, so at the end of the grant period we will have 4.5 years of accrual completed and 1.5 additional years of follow-up. Moderate sized, clinically meaningful differences (HR 1.4-1.8) in median progression free survival (PFS, defined as TTCR) can be detected with good power for variants with a prevalence of 10% or greater in both sets. In **Table 2**, we frame the associations in terms of the variant having better TTCR and response than those without the variant. The opposite is also expected for some variants and the inverse HR could be detected with comparable power.

Although not specified as a primary aim, a hypothesis of interest would be the exploration of interactions of variants with racial groups. In the previous three SWOG coordinated Phase III trials of this patient population, 18-24% of the patients were African American; we would expect a similar proportion in this trial.

Using the whole cohort (n=1200), and assuming 20% African American participation (n=240) and a genetic variant with 30% prevalence, then there will be 80% power to detect heterogeneity by race if the variant HR for TTCR is 1.0 for white patients versus 1.8 for African American patients. We will also have good power to test whether the prevalence of an identified SNP varies by racial

SWOG Authorized Document

group. (80% power to detect an absolute difference of 11% in SNP prevalence between African Americans and whites).

Aim 2: Association of somatic androgen pathway genes and in CTCs and clinical outcomes. We expect to start CTC collection in early to mid 2014 and anticipate having accrued approximately 10% of patients at that point. Of the remaining 90% to accrue, we expect to get a baseline usable CTC sample on 70% resulting in a total of 945 samples (1500 x 0.63) at study entry and approximately 800 at the time of progression (based on prior sample compliance at progression). First, we will compare sequences obtained from CTCs to those obtained from germline DNA (in Aim 1) for the same individual, to identify acquired somatic variants in the CTCs. Measures of pairwise concordance will be reported. Pairwise correlations of variants with one another and with RNA will also be described. We will estimate the frequency of somatic sequence variants, as in Aim 1, and will evaluate their association with PSA response and TTCR. Patients with evaluable CTC measures at baseline will be randomly split into training and validation groups (60%, n=567; 40%, n=378, respectively). Univariate associations with PSA response and TTCR will be evaluated in the training set using a significance level of 0.05 since we will have the validation cohort to help protect against false positive results, and multivariate models will be fit using the previously specified covariates in Aim 1 and other identified variants using LASSO methods. Refined models will be evaluated in the validation data set. Minimally detectable effect sizes will be of the same magnitude, but slightly larger, than those in **Table 2**.

Underlying distributions of RNA expression levels at both time points will be explored using boxplots, density and scatter plots and appropriate transformations will be made to the data. The association of these measures with outcome will be initially evaluated with t-tests (PSA response) and Cox regression (TTCR). Covariate adjustment and multivariate RNA and variant models will be built in the training set and then further evaluated in the validation data set. Change in RNA expression and variant status from study entry to time of progression will be described. Once the primary trial has been reported, the association of change in RNA with OS will be evaluated in a Cox model with RNA change parameterized as a time dependent covariate entering the model at the time of progression. We will also test whether the RNA at progression contributes to the model beyond having the baseline RNA value. Assuming 5 year survival and for illustrative purposes splitting a given RNA value at the median, there will be 85% power to detect a survival HR of 1.30 for those with a RNA value above versus below the median. This also assumes there is no treatment effect. Potential interactions with treatment will be evaluated. As an exploratory sub-aim, comparisons of gene expression from whole transcriptomes between 10 PSA responders and 10 non-responders will be evaluated to discover novel candidates. RNA sequence reads will be analyzed using the Tuxedo suite of analysis tools. *(44)* Cufflinks will be used to estimate relative transcript abundances in terms of RPKM (reads per kilobase of exonic sequence per million mapped reads) and to identify differentially expressed transcripts. *(45)*

Aim 3: Association of androgen pathway genes in primary tumor cells and clinical outcomes. Based on prior SWOG advanced PC trials, roughly 30% of the 1500 enrolled patients will have undergone a prostatectomy. We expect to obtain adequate tissue samples from 80% of those individuals (n=360). Although a sample size of 360 is modest and will not support a training/validation approach in this aim as done in Aims 1 and 2, we will assess variant-response associations in a preliminary fashion in the pooled data set. For example, there will be 80% power to detect a PFS HR of 2.76 (1.76) when a variant has 5% (20%)

SWOG Authorized Document

prevalence. In addition, somatic variants identified from the prostate tissue will be compared with somatic variants in CTCs and germline DNA from the same individual. Measures of variant status agreement within an individual will be estimated. Within subject differences in RNA between the prostatectomy and CTC specimen will also be estimated and tested using paired t-test. We will obtain the false discovery rate for the data, and apply a 5% cut-off rule. Change in expression from prostatectomy to baseline CTC will be correlated with TTCR using a proportional hazards regression model.

Identifying patient groups based on response to ADT. We will identify germline variants (Aim 1) and additional variants and RNA from the CTCs and tumors (Aims 2 and 3) that can be used along with other known prognostic factors (such as PSA, bone pain, extent of disease) to evaluate outcomes among patients being treated with hormonal therapy. In addition to evaluating univariate and multivariate associations with PSA response and TTCR, we will also use logic regression and recursive partitioning to identify groups of patients with similar outcomes. Prognostic groups will be identified in the training set and further evaluated in the validation set. The emphasis of this grant is to better understand the mechanism of action of hormonal therapy and identify patient groups with similar responses while on hormonal therapy. Because all patients will be treated with hormonal therapy, we will initially identify prognostic groups who respond to hormonal therapy across both treatment arms, and subsequently, we will analyze treatment interactions to identify predictive groups of patients who respond to one type of hormonal therapy over another. Although outcome by treatment arm may be revealed beyond the initial funding period for this proposal, SWOG has committed the statistical resources to conduct these additional analyses. ROC curves can be used to evaluate the strength of prediction of these risk models. A risk score using standard risk factors in a logistic model can be used to estimate the area under the ROC curve (AUC) for PSA response and TTCR at a landmark such as 3 years (Yes vs. No). A subsequent validated risk model incorporating variant and RNA data will also be used to estimate the AUC. A comparison of the AUC from the simple model and the enhanced model will evaluate the contribution of the factors identified in this grant.

11.5 Data and Safety Monitoring Committee Oversight

A Data and Safety Monitoring Committee will oversee the conduct of this trial. A majority of the voting members of the DSMC are from outside of SWOG, and at least one outside member is a patient advocate, and one is a statistician. The Group Statistician (or designee), two representatives from the Cancer Therapy Evaluation Program (CTEP) (one physician and one statistician) and one representative of the Division of Cancer Prevention (DCP) are non-voting members. The members of this Committee will receive confidential reports every 6 months from the SWOG Statistical Center, and will meet at the Group's bi-annual meetings or hold conference calls as necessary. They will receive additional reports, if needed, for assessment of Adverse Events, or other study related matters. The Committee will be responsible for decisions regarding possible termination and/or early reporting of the study, and any major study amendments.

In addition to the above DSMC review, toxicity and accrual monitoring are done routinely by the Study Chair, Study Statistician and the Disease Committee Chair. Endpoint monitoring is done by the study Statistician and Study Chair. Accrual reports are generated weekly, and formal toxicity reports are generated every 6 months. In addition, the Statistical Center, Adverse Event Coordinator at the Operations Office, SAE Physician Reviewer, and Study Chair monitor toxicities on an ongoing basis.

SWOG Authorized Document

12.0 DISCIPLINE REVIEW

There will be no formal discipline review done in conjunction with this study.

13.0 REGISTRATION GUIDELINES

13.1 Registration Timing

Patients in the early induction group must be registered prior to initiation of LHRH (no more than ten working days prior to planned start of LHRH). Patients in the late induction group must be registered within 30 days after first injection of the LHRH agonist or antagonist.

13.2 Investigator/Site Requirements

This study is supported by the NCI Cancer Trials Support Unit (CTSU).

Prior to the recruitment of a patient for this study, investigators must be registered members of a Cooperative Group. Each investigator must have an NCI investigator number and must maintain an "active" investigator registration status through the annual submission of a complete investigator registration packet (FDA Form 1572 with original signature, current CV, Supplemental Investigator Data Form with signature and Financial Disclosure Form with original signature) to the Pharmaceutical Management Branch, CTEP, DCTD, NCI. These forms are available on the CTSU Web site (http://ctep.cancer.gov/investigatorResources/investigator_registration.htm). Questions should be directed to the CTEP Investigator Registration Help Desk by e-mail at pmbregpend@ctep.nci.nih.gov.

Each investigator or group of investigators at a clinic site must obtain IRB approval for this protocol and submit IRB approval and supporting documentation to the CTSU Regulatory Office before they can enroll patients. Study centers can check the status of their registration packets by querying the Regulatory Support System (RSS) site registration status page of the CTSU member web site by entering credentials at https://www.ctsu.org.

Requirements for site registration:

- CTSU IRB Certification
- CTSU IRB/Regulatory Approval Transmittal Sheet

Note: Sites participating on the NCI CIRB initiative and accepting CIRB approval for the study are not required to submit separate IRB approval documentation to the CTSU Regulatory Office for initial, continuing or amendment review. This information will be provided to the CTSU Regulatory Office from the CIRB at the time the site's Signatory Institution accepts the CIRB approval. The Signatory site may be contacted by the CTSU Regulatory Office or asked to complete information verifying the participating institutions on the study. Other site registration requirements (i.e., laboratory certifications, protocol-specific training certifications, or modality credentialing) must be submitted to the CTSU Regulatory Office or compliance communicated per protocol instructions.

13.3 OPEN Registration Requirements

The individual registering the patient must have completed the appropriate SWOG Registration Worksheet. The completed form must be referred to during the registration but should not be submitted as part of the patient data.

SWOG Authorized Document

Oncology Patient Enrollment Network (OPEN) will also ask additional questions that are not present on the SWOG Registration Worksheet. The individual registering the patient must be prepared to provide answers to the following questions:

a. Protocol Number

b. Institution CTEP ID

c. Registration Step

d. Treating Investigator

e. Cooperative Group Credit

f. Credit Investigator

g. Patient Initials

h. Patient's Date of Birth

i. Patient SSN (SSN is desired, but optional. Do not enter invalid numbers.)

j. Country of Residence

k. ZIP Code

l. Gender (select one):

- Female Gender
- Male Gender

m. Ethnicity (select one):

- Hispanic or Latino
- Not Hispanic or Latino
- Unknown

n. Method of Payment (select one):

- Private Insurance
- Medicare
- Medicare and Private Insurance
- Medicaid
- Medicaid and Medicare
- Military or Veterans Sponsored NOS
- Military Sponsored (Including Champus & Tricare)
- Veterans Sponsored
- Self Pay (No Insurance)
- No Means of Payment (No Insurance)
- Other
- Unknown

o. Race (select all that apply):

- American Indian or Alaska Native
- Asian
- Black or African American
- Native Hawaiian or other Pacific Islander

SWOG Authorized Document

- White
- Unknown

13.4 Registration Procedures

a. All site staff (SWOG and CTSU Sites) will use OPEN to enroll patients to this study. OPEN is a web-based application that is integrated with the CTSU Enterprise System for regulatory and roster data and, at the time of patient registration, initializes the patient in the Rave database. OPEN can be accessed at https://open.ctsu.org or from the OPEN tab on the CTSU members' side of the website at https://www.ctsu.org, or from the OPEN Patient Registration link on the SWOG CRA Workbench.

b. Prior to accessing OPEN site staff should verify the following:

- All eligibility criteria have been met within the protocol stated timeframes. Site staff should refer to Section 5.0 to verify eligibility.

- All patients have signed an appropriate consent form and HIPAA authorization form (if applicable).

- The study site is listed as "approved" in the CTSU RSS.

c. Access requirements for OPEN:

- Site staff will need to be registered with CTEP and have a valid and active CTEP-IAM account. This is the same account (user ID and password) used for the CTSU members' web site. Additional information about obtaining a CTEP-IAM account can be found at http://ctep.cancer.gov/branches/pmb/associate_registration.htm. Questions should be directed to the CTEP Associate Registration Help Desk by e-mail at ctepreghelp@ctep.nci.nih.gov.

- To perform registrations, the site user must have been assigned the 'Registrar' role on the SWOG or CTSU roster:

  1. *If you are a SWOG member, to perform registrations on SWOG protocols you must have an equivalent 'Registrar' role on the SWOG roster. Role assignments are handled through SWOG.*

  2. If you are not a SWOG member, to perform registrations on SWOG protocols you must have the role of Registrar on the CTSU roster. Site and/or Data Administrators can manage CTSU roster roles via the new Site Roles maintenance feature under RSS on the CTSU members' web site. This will allow them to assign staff the "Registrar" role.

Note: The OPEN system will provide the site with a printable confirmation of registration and treatment information. Please print this confirmation for your records.

d. Further instructional information is provided on the OPEN tab of the CTSU members' side of the CTSU website at https://www.ctsu.org or at https://open.ctsu.org. For any additional questions contact the CTSU Help Desk at 1-888-823-5923 or ctsucontact@westat.com.

SWOG Authorized Document

13.5 Exceptions to SWOG registration policies will not be permitted.

a. Patients must meet all eligibility requirements.

b. Institutions must be identified as approved for registration.

c. Registrations may not be cancelled.

d. Late registrations (after initiation of treatment) will not be accepted.

14.0 DATA SUBMISSION SCHEDULE

14.1 Data Submission Requirement

Data must be submitted according to the protocol requirements for **ALL** patients registered, whether or not assigned treatment is administered, including patients deemed to be ineligible. Patients for whom documentation is inadequate to determine eligibility will generally be deemed ineligible.

14.2 Master Forms

Master forms can be found on the protocol abstract page on the SWOG website (www.swog.org) and (with the exception of the sample consent form and the Registration Worksheet) must be submitted on-line via the Web; see Section 14.3a for details.

14.3 Data Submission Procedures

a. SWOG institutions must submit data electronically via the Web using Medidata Rave at the following url:

https://login.imedidata.com/selectlogin

1. If prompted, select the 'CTEP-IAM IdP' link.

2. Enter your valid and active CTEP-IAM userid and password. This is the same account used for the CTSU members' web site and OPEN.

b. You may also access Rave® via the SWOG CRA Workbench. Go to the SWOG web site (http://swog.org) and logon to the Members Area using your SWOG Roster ID Number and password. After you have logged on, click on *Workbenches*, then *CRA Workbench* to access the home page for the CRA Workbench and follow the link to Rave® provided in the left-hand navigation panel.

To access the CRA Workbench the following must be done (in order):

1. You are entered into the SWOG Roster and issued a SWOG Roster ID Number,
2. You are associated as an investigator or CRA/RN at the institution where the patient is being treated or followed,
3. Your Web User Administrator has added you as a web user and has given you the appropriate system permissions to view data for that institution.

SWOG Authorized Document

For assistance with points 1 and 2 call the Operations Office at 210/614-8808. For point 3, contact your local Web User Administrator (refer to the "Who is my Web User Administrator?" function on the swog.org Members logon page).

For difficulties with the CRA Workbench, please email technicalquestion@crab.org.

c. Institutions participating through the Cancer Trials Support Unit (CTSU) please refer to the CTSU Participation Table on Page 3.

14.4 Data Submission Overview and Timepoints

a. WITHIN 7 DAYS OF REGISTRATION

Submit copies of the following:

S1216 Onstudy Form

Baseline Tumor Assessment Form

S1216 Prostate Specific Antigen Reporting Form

Pathology Report

Radiology Report from all scans performed to assess disease at baseline

b. WITHIN 14 DAYS OF REGISTRATION; WITHIN 14 DAYS AFTER COMPLETION OF PROTOCOL TREATMENT FOR MONTHS 6 AND 12 (OR PROGRESSION, WHICHEVER OCCURS FIRST): ***(Note: Effective September 23, 2014, androgen testing has been permanently closed to new patients.)***

Submit serum for androgen testing as outlined in Section 15.1.

c. IF PATIENT CONSENTED, WITHIN 14 DAYS OF REGISTRATION:

Submit tissue specimens as outlined in Section 15.2.

d. IF PATIENT CONSENTED, WITHIN 14 DAYS OF REGISTRATION; AND WITHIN 14 DAYS OF COMPLETING 6 MONTHS OF PROTOCOL TREATMENT; AND WITHIN 14 DAYS OF DISEASE PROGRESSION OR REMOVAL FROM PROTOCOL TREATMENT:

Submit plasma/buffy coat specimens as outlined in Section 15.2.

e. IF PATIENT CONSENTED, WITHIN 14 DAYS OF REGISTRATION; AND WITHIN 14 DAYS OF DISEASE PROGRESSION OR REMOVAL FROM PROTOCOL TREATMENT:

Submit peripheral whole blood specimens as outlined in Section 15.2.

f. WITHIN 14 DAYS OF COMPLETION OF EVERY MONTH OF PROTOCOL TREATMENT FOR MONTHS 1-4; AFTER FOUR MONTHS OF PROTOCOL TREATMENT, SUBMIT WITHIN 14 DAYS OF COMPLETION OF EVERY THIRD MONTH OF PROTOCOL TREATMENT:

Submit the following:

S1216 Adverse Event Form

SWOG Authorized Document

S1216 Prostate Specific Antigen Reporting Form

g. WITHIN 14 DAYS OF COMPLETION OF EACH CYCLE (EVERY 90 DAYS) OF PROTOCOL TREATMENT:

Submit the following:

S1216 Treatment Form

h. WITHIN 14 DAYS OF EVERY DISEASE ASSESSMENT WHILE PATIENT IS ON PROTOCOL TREATMENT:

Submit the following:

Follow Up Tumor Assessment Form

S1216 Prostate Specific Antigen Reporting Form

Radiology Report from all scans performed to assess disease

i. WITHIN 14 DAYS OF DISCONTINUATION OF ALL PROTOCOL TREATMENT:

Submit the following:

Off Treatment Notice

Final **S1216** Treatment Form

Final **S1216** Adverse Event Form

S1216 Prostate Specific Antigen Reporting Form

j. AFTER OFF ALL PROTOCOL TREATMENT, EVERY SIX MONTHS FOR TWO YEARS AND ANNUALLY UNTIL TEN YEARS AFTER REGISTRATION OR UNTIL DEATH:

Submit the following:

S1216 Prostate Specific Antigen Reporting Form

Follow Up Form

k. WITHIN 14 DAYS OF PROGRESSION/RELAPSE:

Submit the following:

If patient was still on protocol treatment:

S1216 Treatment Form

S1216 Adverse Event Form

S1216 Prostate Specific Antigen Reporting Form

Off Treatment Notice

Follow-Up Tumor Assessment Form

SWOG Authorized Document

If patient was off protocol treatment:

Follow Up Form

l. WITHIN 4 WEEKS OF KNOWLEDGE OF DEATH:

If patient was still on protocol treatment:

Off Treatment Notice

Notice of Death

Final S1216 Treatment Form

Final S1216 Adverse Event Form

S1216 Prostate Specific Antigen Reporting Form

If patient was off protocol treatment:

Notice of Death

Follow Up Form

15.0 SPECIAL INSTRUCTIONS

Specimens are required for androgen testing and must be submitted to ARUP Laboratories, Lab #208. Specimens for banking and translational medicine studies are optional for the patient, and are to be submitted to the SWOG Specimen Repository – Solid Tissue, Myeloma and Lymphoma Division, Lab #201.

15.1 Androgen Testing (required for the patient)

Submission of serum was required **only for the first 300 patients** for androgen testing (i.e. testosterone and dehydroepiandrosterone sulfate [DHEA-S]). (**Note: Effective September 23, 2014, androgen testing has been permanently closed to new patients.**)

a. Specimen Submission Timepoints (**only for patients registered between January 1, 2014 and September 22, 2014**)

- Prestudy (prior to C1D1);
- Month 7;
- Month 13 or progression (whichever occurs first).

b. Serum processing instructions

Collect approximately six milliliters (6 mL) of whole blood in a serum separator tube (SST). Please process blood into serum according to the specimen submission instructions (see Section 15.2) and equally aliquot serum into two plastic storage/transport or cryotubes. **Specimens should be stored frozen at -20°C and shipped frozen to ARUP Laboratories, Lab #208**.

c. Specimen collection kits are not being provided for this submission; sites will use institutional supplies.

SWOG Authorized Document

15.2 Specimens for banking and translational medicine studies (optional for the patient)

NOTE: For Arm 1, C1D1 is considered the first day of TAK-700 treatment. For Arm 2, C1D1 is considered the day of randomization. In the event of a late blood draw (due to patient scheduling issues), please collect and submit the baseline blood specimen and note the reason for the delay in the SWOG Specimen Tracking System (STS).

With patient's consent, the following specimens must be submitted (see Section 9.0):

a. Tissue block or 30 unstained slides (tissue from radical prostatectomy or biopsy) at prestudy (prior to C1D1).

A pathologist at each institution should review hematoxylin and eosin stained slides corresponding to each FFPE tissue block and will choose the tissue block that contains the highest percentage of tumor tissue that is representative of the entire tumor Gleason score. Using this block, 30 sections of 5-micron thickness should be cut and mounted onto glass slides without coverslips.

If the prostatectomy was performed at an institution other than the one where the patient currently is being treated on **S1216**, an effort should be made to contact the institution where the prostatectomy was performed and procure the FFPE blocks or slide sections from those blocks. Tissue (or slides) should be submitted to Lab #201.

NOTE: Radical prostatectomy specimens are preferred. However, if patient biopsy tissue is not from a radical prostatectomy, please submit at least 10-15 5-micron unstained slides or FFPE tissue from biopsy.

1. Tissue submission instructions can be accessed on the SWOG Specimen Submission webpage: SOLID TUMOR (http://swog.org/Members/ClinicalTrials/Specimens/STSpecimens.asp), or via the link on the **S1216** protocol abstract page on the SWOG website (www.swog.org).

2. Specimen collection kits are not being provided for this submission; sites will use institutional supplies.

b. Buffy Coat & Plasma

Fifteen milliliters (15 mL) of whole blood (for buffy coat and plasma specimen banking) must be submitted at the following times: prestudy (prior to C1D1), Month 7 (plasma only), and at progression or removal from protocol treatment (plasma only). Please process specimen into plasma and then equally aliquoted into 2-mL plastic storage/transport or cryotubes. Aliquot the buffy coat equally into 2-mL cryotubes (only required at prestudy). Plasma & buffy coat specimens should be submitted to Lab #201.

1. Buffy coat and plasma submission instructions can be accessed on the SWOG Specimen Submission webpage: SOLID TUMOR (http://swog.org/Members/ClinicalTrials/Specimens/STSpecimens.asp), or via the link on the **S1216** protocol abstract page on the SWOG website (www.swog.org).

2. Specimen collection kits are not being provided for this submission; sites will use institutional supplies.

SWOG Authorized Document

c. Peripheral Whole Blood

Whole blood for germline (white blood cell - WBC) and somatic (circulating tumor cell - CTC) androgen pathway analysis must be submitted at the following times: prestudy (prior to C1D1) and at progression or removal from protocol treatment. Peripheral whole blood must be drawn and shipped Monday-Thursday only. Whole blood cannot be stored over the weekend and the Goldkorn lab cannot routinely accept Saturday delivery. If a sample cannot absolutely be drawn and shipped Monday-Thursday, please contact the Goldkorn lab directly to discuss whether special arrangements can be made.

At each of these two time points, 8-10 ml of peripheral blood will be drawn by standard venipuncture into one CellSave tube and three EDTA tubes (~35 ml total). Complete blood collection and mailing kits will be mailed in advance to participating centers. **Please email or call the contacts below to receive your kits. Every kit will include a complete collection and mailing instruction sheet (also provided in Section 18.3).** Briefly, the blood from one of the EDTA tubes will be transferred into a LiquidBiopsy tube ("Tube A") containing a fixative. Then, the LiquidBiopsy tube, along with the CellSave and EDTA tubes, will be re-packaged back into the mailing kits and shipped on the day of collection by overnight FedEx (packing slip provided) to the Goldkorn Laboratory at USC. At the Goldkorn Laboratory, the tubes will be processed for collection of WBC pellet and CTC enrichment, for subsequent genomic and gene expression profiling of androgen pathway genes. Peripheral whole blood specimens should be submitted to Lab #181.

1. Peripheral whole blood specimens for germline (WBC) and somatic (CTC) androgen pathway analysis will be re-packaged back into the mailing kits and shipped on the day of collection by overnight delivery. A FedEx packing slip will be provided. Specimens should be shipped to:

   Lab#181:
   Amir Goldkorn Lab
   USC Norris Comprehensive Cancer Center
   Harlyne Norris Research Tower, Room 6516
   1450 Biggy Street
   Los Angeles, CA 90033-1006
   Tel: 323/442-7722

   Contacts for this correlative study (in order of preference):
   Yucheng Xu, Ph.D. (Goldkorn Lab): yuchengx@usc.edu
   Tong Xu, Ph.D. (Goldkorn Lab): tongxu@usc.edu
   Amir Goldkorn, M.D.: agoldkor@usc.edu
   Shigang Xiong, M.D. (Pinski Lab): shigangx@usc.edu
   Jacek Pinski, M.D., Ph.D.: pinski@med.usc.edu
   Susan Ingles, Ph.D.: sueanningles@gmail.com

2. Specimen collection kits may be ordered by contacting Dr. Amir Goldkorn's laboratory at the University of Southern California (323/442-7722).

15.3 General Specimen Submission Instructions

a. SWOG Specimen Tracking System (STS)

All specimen submissions for this study must be entered and tracked using the SWOG online Specimen Tracking system. SWOG members may log on the online system via the CRA Workbench. To access the CRA Workbench, go to

the SWOG Web site (http://swog.org) and logon to the Members Area. After you have logged on using your SWOG roster ID number and password, click on the *CRA Workbench* link to access the home page for CRA Workbench website. Non- SWOG users may log into SpecTrack using their CTSU UserID and password on the SpecTrack login page located at https://crawb.crab.org/SpecTrack/Logon.aspx (select the option "SWOG – SWOG – CTSU"). SpecTrack start-up instructions (both written and demo) are available after signing in to SpecTrack.

A copy of the Shipment Packing List produced by the online Specimen Tracking system should be printed and placed in the pocket of the specimen bag if it has one, or in a separate resealable bag. The Specimen Submission Form is NOT required when the online system is used.

ALL SPECIMENS MUST BE LOGGED VIA THIS SYSTEM; THERE ARE NO EXCEPTIONS.

To report technical problems with Specimen Tracking, such as database errors or connectivity issues, please send an email to technicalquestion@crab.org. For procedural help with logging and shipping specimens, there is an introduction to the system on the Specimen Tracking main page (http://dnet.crab.org/SpecTrack/Documents/Instructions.pdf); or contact the Data Operations Center at 206/667-2267 to be routed to the Data Coordinator for further assistance.

In the online specimen tracking system, the appropriate SWOG laboratories are identified as follows:

<u>Serum (for androgen testing)</u>
Lab#208: ARUP Laboratories
Contact: Jonathan Lowe
Phone: 801/583-2787
E-mail: jonathan.lowe@aruplab.com

<u>Tissue, buffy coat, and plasma (for banking)</u>
Lab#201: SWOG Specimen Repository
Contact: Erin Grundy
Phone: 614/355-3099
Email: bpcbank@nationwidechildrens.org

<u>Whole blood (for translational medicine)</u>
Lab#181: Amir Goldkorn Laboratory
Contact: Yucheng Xu
Phone: 323/442-7722
Email: yuchengx@usc.edu

b. Federal guidelines for the shipment of blood products:

1. The tube must be wrapped in an absorbent material.

2. The tube must then be placed in an AIRTIGHT container (like a resealable bag).

3. Pack the resealable bag and tube in a Styrofoam shipping container.

4. Pack the Styrofoam shipping container in a cardboard box.

5. Mark the box "Biohazard".

SWOG Authorized Document

16.0 ETHICAL AND REGULATORY CONSIDERATIONS

The following must be observed to comply with Food and Drug Administration regulations for the conduct and monitoring of clinical investigations; they also represent sound research practice:

Informed Consent

The principles of informed consent are described by Federal Regulatory Guidelines (Federal Register Vol. 46, No. 17, January 27, 1981, part 50) and the Office for Protection from Research Risks Reports: Protection of Human Subjects (Code of Federal Regulations 45 CFR 46). They must be followed to comply with FDA regulations for the conduct and monitoring of clinical investigations.

Institutional Review

This study must be approved by an appropriate institutional review committee as defined by Federal Regulatory Guidelines (Ref. Federal Register Vol. 46, No. 17, January 27, 1981, part 56) and the Office for Protection from Research Risks Reports: Protection of Human Subjects (Code of Federal Regulations 45 CFR 46).

Drug Accountability

An investigator is required to maintain adequate records of the disposition of investigational drugs according to procedures and requirements governing the use of investigational new drugs as described in the Code of Federal Regulations 21 CFR 312.

Monitoring

This study will be monitored by the Clinical Data Update System (CDUS) Version 3.0. Cumulative CDUS data will be submitted quarterly to CTEP by electronic means. Reports are due January 31, April 30, July 31 and October 31.

Confidentiality

Please note that the information contained in this protocol is considered confidential and should not be used or shared beyond the purposes of completing protocol requirements until or unless additional permission is obtained.

16.1 Adverse Event Reporting Requirements

a. Purpose

Adverse event data collection and reporting, which are required as part of every clinical trial, are done to ensure the safety of patients enrolled in the studies as well as those who will enroll in future studies using similar agents. Adverse events are reported in a routine manner at scheduled times during a trial. (Directions for routine reporting are provided in Section 14.0.) Additionally certain adverse events must be reported in an expedited manner to allow for more timely monitoring of patient safety and care. The following guidelines prescribe expedited adverse event reporting for this protocol.

b. Reporting method

This study requires that expedited adverse events be reported using the Cancer Therapy Evaluation Program Adverse Event Reporting System (CTEP-AERS). CTEP's guidelines for CTEP-AERS can be found at http://ctep.cancer.gov. A CTEP-AERS report must be submitted to the SWOG Operations Office electronically via the CTEP-AERS Web-based application located at:
http://ctep.cancer.gov/protocolDevelopment/electronic_applications/adverse_events.htm.

SWOG Authorized Document

c. When to report an event in an expedited manner

Some adverse events require 24-hour notification (refer to Table 16.1) via CTEP-AERS. When Internet connectivity is disrupted, a 24-hour notification is to be made to SWOG by telephone at 210-614-8808 or by email at adr@swog.org. Once Internet connectivity is restored, a 24-hour notification that was made by phone or using adr@swog.org must be entered electronically into CTEP-AERS by the original submitter at the site.

When the adverse event requires expedited reporting, submit the report within the number of calendar days of learning of the event, as specified in Table 16.1 or 16.2, as applicable.

d. Other recipients of adverse event reports

The SWOG Operations Office will forward reports and documentation to the appropriate regulatory agencies and drug companies as required.

Adverse events determined to be reportable to the Institutional Review Board responsible for oversight of the patient must be reported according to local policy and procedures.

e. **Expedited reporting for investigational agents**

Expedited reporting is required if the patient has received at least one dose of the investigational agent(s) as part of the trial. Reporting requirements are provided in Table 16.1. The investigational agent used in Arm 1 of this study is TAK-700 (Orteronel). If there is any question about the reportability of an adverse event or if on-line CTEP-AERS cannot be used, please telephone or email the SAE Specialist at the SWOG Operations Office, 210/614-8808 or adr@swog.org, before preparing the report.

SWOG Authorized Document

Table 16.1:

Late Phase 2 and Phase 3 Studies: Expedited Reporting Requirements for Adverse Events that Occur on Studies under a Non-CTEP IND within 30 Days of the Last Administration of the Investigational Agent/Intervention[1] TAK-700 (Arm 1)

FDA REPORTING REQUIREMENTS FOR SERIOUS ADVERSE EVENTS (21 CFR Part 312)
NOTE: Investigators **MUST** immediately report to the sponsor (NCI) **ANY** Serious Adverse Events, whether or not they are considered related to the investigational agent(s)/intervention (21 CFR 312.64)

An adverse event is considered serious if it results in **ANY** of the following outcomes:

1) Death
2) A life-threatening adverse event
3) An adverse event that results in inpatient hospitalization or prolongation of existing hospitalization for ≥ 24 hours
4) A persistent or significant incapacity or substantial disruption of the ability to conduct normal life functions
5) A congenital anomaly/birth defect.
6) Important Medical Events (IME) that may not result in death, be life threatening, or require hospitalization may be considered serious when, based upon medical judgment, they may jeopardize the patient or subject and may require medical or surgical intervention to prevent one of the outcomes listed in this definition. (FDA, 21 CFR 312.32; ICH E2A and ICH E6).

ALL SERIOUS adverse events that meet the above criteria **MUST** be immediately reported to the NCI via CTEP-AERS within the timeframes detailed in the table below.

<table>
<tr><th>Hospitalization</th><th>Grade 1 Timeframes</th><th>Grade 2 Timeframes</th><th>Grade 3 Timeframes</th><th>Grade 4 & 5 Timeframes</th></tr>
<tr><td>Resulting in Hospitalization ≥ 24 hrs</td><td colspan="3">10 Calendar Days</td><td rowspan="2">24-Hour 5 Calendar Days</td></tr>
<tr><td>Not resulting in Hospitalization ≥ 24 hrs</td><td colspan="2">Not required</td><td>10 Calendar Days</td></tr>
</table>

NOTE: Protocol specific exceptions to expedited reporting of serious adverse events are found in the Specific Protocol Exceptions to Expedited Reporting (SPEER) portion of the CAEPR or [Section 16.1f.]

Expedited AE reporting timelines are defined as:

- o "24-Hour; 5 Calendar Days" - The AE must initially be reported via CTEP-AERS within 24 hours of learning of the AE, followed by a complete expedited report within 5 calendar days of the initial 24-hour report.
- o "10 Calendar Days" - A complete expedited report on the AE must be submitted within 10 calendar days of learning of the AE.

[1]Serious adverse events that occur more than 30 days after the last administration of investigational agent/intervention and have an attribution of possible, probable, or definite require reporting as follows:

Expedited 24-hour notification followed by complete report within 5 calendar days for:

- All Grade 4, and Grade 5 AEs

Expedited 10 calendar day reports for:

- Grade 2 adverse events resulting in hospitalization or prolongation of hospitalization
- Grade 3 adverse events

May 5, 2011

SWOG Authorized Document

f. **Additional Instructions or Exceptions to CTEP-AERS Expedited Reporting Requirements for Late Phase 2 and Phase 3 Studies Utilizing an Agent under a non-CTEP-IND:**

1. **Group-specific instructions.**

Supporting Documentation Submission - Within 5 **calendar days** submit the following to the SWOG Operations Office by fax to 210-614-0006 or mail to the address below:

- Printed copy of the first page of the CTEP-AERS report
- Copies of clinical source documentation of the event
- If applicable, and they have not yet been submitted to the SWOG Data Operations Center, copies of Off Treatment Notice and/or Notice of Death.

g. **Expedited reporting for <u>commercial</u> agents**

Commercial reporting requirements are provided in Table 16.2. The commercial agents used in Arms 1 and 2 of this study are leuprolide or goserelin and bicalutamide (Arm 2 only). If there is any question about the reportability of an adverse event, or if on-line CTEP-AERS cannot be used please telephone or email the SAE Program at the SWOG Operations Office, 210/614-8808 or adr@swog.org, before preparing the report.

Table 16.2. Expedited reporting requirements for adverse events experienced by patients on study Arms 1 and 2 who have received the commercial drug(s) listed in Section 16.1g above within 30 days of the last administration of the commercial agent(s).

Attribution	Grade 4		Grade 5[a]	
	Unexpected	Expected	Unexpected	Expected
Unrelated or Unlikely			**CTEP-AERS**	**CTEP-AERS**
Possible, Probable, Definite	**CTEP-AERS**		**CTEP-AERS**	**CTEP-AERS**

CTEP-AERS: Indicates an expedited report is to be submitted via NCI CTEP-AERS within 10 calendar days of learning of the event[b].

[a] This includes all deaths within 30 days of the last dose of treatment with a commercial agent(s), regardless of attribution. Any death that occurs more than 30 days after the last dose of treatment with a commercial agent(s) and is attributed (possibly, probably, or definitely) to the agent(s) and is not due to cancer recurrence must be reported according to the instructions above.

[b] Submission of the on-line CTEP-AERS report plus any necessary amendments generally completes the reporting requirements. You may, however, be asked to submit supporting clinical data to the SWOG Operations Office in order to complete the evaluation of the event. If requested, the specified data should be sent within 5 calendar days by fax to 210-614-0006.

h. **Reporting Secondary Malignancy, including AML/ALL/MDS**

1. A secondary malignancy is a cancer caused by treatment for a previous malignancy (e.g., treatment with investigational agent/intervention,

SWOG Authorized Document

radiation or chemotherapy). A secondary malignancy is not considered a metastasis of the initial neoplasm.

SWOG requires all secondary malignancies that occur following treatment with an agent under a non-NCI IND to be reported via CTEP-AERS. Three options are available to describe the event.

- Leukemia secondary to oncology chemotherapy (e.g., Acute Myelocytic Leukemia [AML])
- Myelodysplastic syndrome (MDS)
- Treatment-related secondary malignancy

Any malignancy possibly related to cancer treatment (including AML/MDS) should also be reported via the routine reporting mechanisms outlined in each protocol.

Second Malignancy: A second malignancy is one unrelated to the treatment of a prior malignancy (and is NOT a metastasis from the initial malignancy). Second malignancies require ONLY routine reporting via CDUS unless otherwise specified.

For more information see:
http://ctep.cancer.gov/protocolDevelopment/electronic_applications/docs/aeguidelines.pdf

2. Supporting documentation should be submitted to CTEP in accordance with instructions provided by the CTEP-AERS system. A copy of the report and the following supporting documentation must also be submitted to the SWOG Operations Office within 30 days by fax to 210-614-0006 or mail to the address below:

- a copy of the pathology report confirming the AML/ALL /MDS diagnosis
- (if available) a copy of the cytogenetics report

SWOG
ATTN: SAE Program
4201 Medical Drive, Suite 250
San Antonio, Texas 78229

NOTE: If a patient has been enrolled in more than one NCI-sponsored study, the report must be submitted for the most recent trial.

SWOG Authorized Document

17.0 BIBLIOGRAPHY

1 Jemal A, Siegel R, Ward E, Murray T, Xu J, Thun MJ. Cancer statistics, 2007. CA Cancer J Clin 57(1):43-66, 2007.

2 Chen Y, Clegg NJ, Scher HI. Anti-androgens and androgen-depleting therapies in prostate cancer: new agents for an established target. The Lancet Oncology 10(10):981-991, 2009.

3 Small EJ, Ryan CJ. The case for secondary hormonal therapies in the chemotherapy age. J Urol 176(Suppl 1):S66-S71, 2006.

4 Chen Y, Clegg NJ, Scher HI. Anti-androgens and androgen-depleting therapies in prostate cancer: new agents for an established target. The Lancet Oncology 10(10):981-991, 2009.

5 Reid AH, Attard G, Barrie E, de Bono JS. CYP17 inhibition as a hormonal strategy for prostate cancer. Nat Clin Pract Urol 5(11):610-620, 2008.

6 Reid AH, Attard G, Barrie E, de Bono JS. CYP17 inhibition as a hormonal strategy for prostate cancer. Nat Clin Pract Urol 5(11):610-620, 2008.

7 Edwards J, Krishna NS, Grigor KM, Bartlett JM. Androgen receptor gene amplification and protein expression in hormone refractory prostate cancer. Br J Cancer, 89(3), 552-556, 2003.

8 Taplin ME. Androgen receptor mutations in androgen-independent prostate cancer: Cancer and Leukemia Group B Study 9663. J Clin Oncol 21:2673-2678, 2003.

9 Steinkamp MP, O'Mahony OA, Brogley M, et al. Treatment-dependent androgen receptor mutations in prostate cancer exploit multiple mechanisms to evade therapy. Cancer Res 69(10):4434-4442. 2009.

10 Edwards J, Krishna NS, Grigor KM, Bartlett JM. Androgen receptor gene amplification and protein expression in hormone refractory prostate cancer. Br J Cancer, 89(3), 552-556, 2003.

11 Taplin ME, Rajeshkumar B, Halabi S, et al. Androgen receptor mutations in androgen-independent prostate cancer: Cancer and Leukemia Group B Study 9663. J Clin Oncol 21(14):2673-2678, 2003.

12 Titus MA, Schell MJ, Lih FB, Tomer KB, Mohler JL. Testosterone and dihydrotestosterone tissue levels in recurrent prostate cancer. Clin Cancer Res 11(13):4653-4657, 2005.

13 Chen Y, Clegg NJ, Scher HI. Anti-androgens and androgen-depleting therapies in prostate cancer: new agents for an established target. The Lancet Oncology 10(10):981-991, 2009.

14 Montgomery RB. Maintenance of intratumoral androgens in metastatic prostate cancer: a mechanism for castration-resistant tumor growth. Cancer Res 68:4447-4454, 2008.

15 Mostaghel EA. Intraprostatic androgens and androgen-regulated gene expression persist after testosterone suppression: therapeutic implications for castration-resistant prostate cancer. Cancer Res 67:5033-5041, 2007.

16 Montgomery RB. Maintenance of intratumoral androgens in metastatic prostate cancer: a mechanism for castration-resistant tumor growth. Cancer Res 68:4447-4454, 2008.

17 Dillard PR, Lin MF, Khan SA. Androgen-independent prostate cancer cells acquire the complete steroidogenic potential of synthesizing testosterone from cholesterol. Mol Cell Endocrinol 295(1-2):115-120, 2008.

SWOG Authorized Document

18 Attard G, Reid AH, A'Hern R, et al. Selective inhibition of CYP17 with abiraterone acetate is highly active in the treatment of castration-resistant prostate cancer. J Clin Oncol 27(23):3742-3748, 2009.

19 Ryan C, Efstathiou E, Smith M, Taplin M, Bubley G, Logothetis C, Kheoh T, Haqq C, Molina A, Small EJ. Phase II multicenter study of chemotherapy (chemo)-naive castration-resistant prostate cancer (CRPC) not exposed to ketoconazole (keto), treated with abiraterone acetate (AA) plus prednisone. J Clin Oncol 27, 15s:abstr 5046, 2009.

20 de Bono JS, Logothetis CJ, Molina A, et al. Abiraterone and increased survival in metastatic prostate cancer. N Engl J Med 364(21):1995-2005, 2011.

21 Agus DB, Stadler WM, Shevrin DH, et al. Safety, efficacy, and pharmacodynamics of the investigational agent orteronel (TAK-700) in metastatic castration-resistant prostate cancer (mCRPC): Updated data from a phase I/II study. J Clin Oncol 30, (suppl 5; abstr 98), 2012.

22 Hussain M, Corn P, Michaelson D, Hammers H, Alumkal J, Ryan C, Bruce J, Moran S, Mortimer P, Lee S-Y, George D. Activity and Safety of the Investigational Agent Orteronel in Men With Nonmetastatic Castration-resistant Prostate Cancer and Rising Prostate-specific Antigen: Results of a Phase 2 Study. 27th Annual European Association of Urology, Paris, France. 2012: February 24–28:Poster 124.

23 Dreicer R, Jones R, Oudard S, et al. Results from a phase 3, randomized, double-blind, multicenter, placebo-controlled trial of orteronel (TAK-700) plus prednisone in patients with metastatic castration-resistant prostate cancer (mCRPC) that has progressed during or following docetaxel-based therapy (ELM-PC 5 trial). J Clin Oncol 32, (suppl 4; abstr 7), 2014.

24 Sweeney C, Chen Y-H, Carducci MA, Liu G, et al. Impact on overall survival (OS) with chemohormonal therapy versus hormonal therapy for hormone-sensitive newly metastatic prostate cancer (mPrCa): An ECOG-led phase III randomized trial. J Clin Oncol 32:5s, (suppl; abstr LBA2), 2014.

25 Crawford ED, Eisenberger MA, McLeod DG, et al. A controlled trial of leuprolide with and without flutamide in prostatic carcinoma. N Engl J Med 321:419-24, 1989.

26 Eisenberger MA, Blumenstein BA, Crawford ED, et al. Bilateral orchiectomy with or without flutamide for metastatic prostate cancer. N Engl J Med 339:1036-42, 1998.

27 Prostate Cancer Trialists' Collaborative Group Maximum androgen blockade in advanced prostate cancer: an overview of the randomized trials. Lancet 355(9214):1491-8, 2000.

28 Schmitt B, Bennett C, Seidenfeld J, Samson D, Wilt TJ. Maximal androgen blockade for advanced prostate cancer. Cochrane Database Syst Rev 2:CD001526, 1999.

29 Samson DJ, Seidenfeld J, Schmitt B, et al. Systematic review and meta-analysis of monotherapy compared with combined androgen blockade for patients with advanced prostate carcinoma. Cancer 95(2):361-76, 2002.

30 Loblaw DA. Initial hormonal management of androgen-sensitive metastatic, recurrent, or progressive prostate cancer: 2007 update of an American Society of Clinical Oncology practice guideline. J Clin Oncol 25:1596-1605, 2007.

31 Klotz L, O'Callaghan C, Ding K, Toren P, et al. Nadir testosterone within first year of androgen-deprivation therapy (ADT) predicts for time to castration-resistant progression: a secondary

SWOG Authorized Document

analysis of the PR-7 trial of intermittent versus continuous ADT. J Clin Oncol 33(10):1151-6, 2015 Apr 1. doi: 10.1200/JCO.2014.58.2973. Epub 2015 Mar 2.

32 Suzman DL, Antonarakis ES. Does degree of androgen suppression matter in hormone-sensitive prostate cancer? J Clin Oncol 33(10):1098-100, 2015 Apr 1. doi: 10.1200/JCO.2014.60.1419. Epub 2015 Mar 2.

33 Hussain M, Tangen CM, Higano C, et al. Absolute prostate-specific antigen value after androgen deprivation is a strong independent predictor of survival in new metastatic prostate cancer: data from Southwest Oncology Group Trial 9346 (INT-0162). J Clin Oncol 24(24):3984-3990, 2006.

34 Sweeney CJ, Chen YH, Carducci M, et al. Chemohormonal therapy in metastatic hormone-sensitive prostate cancer. NEJM 373(8):737-46, 2015 Aug 20. doi: 10.1056/NEJMoa1503747. Epub 2015 Aug 5.

35 Hussain M, Goldman B, Tangen C, et al. Prostate-specific antigen progression predicts overall survival in patients with metastatic prostate cancer: data from Southwest Oncology Group Trials 9346 (Intergroup Study 0162) and 9916. J Clin Oncol 27(15):2450-2456, 2009.

36 Pocock SJ, Simon R. Sequential treatment assignment with balancing for prognostic factors in controlled clinical trials. Biometrics 31:103-115, 1975.

37 Scher HI, Halabi S, Tannock I, et al. Design and end points of clinical trials for patients with progressive prostate cancer and castrate levels of testosterone: recommendations of the Prostate Cancer Clinical Trials Working Group. J Clin Oncol 26(7):1148-1159, 2008.

38 Goldman B, LeBlanc M, Crowley J. Interim futility analysis with intermediate endpoints. Clin Trials 5(1):14-22, 2008.

39 Scher HI, Halabi S, Tannock I, et al. Design and end points of clinical trials for patients with progressive prostate cancer and castrate levels of testosterone: recommendations of the Prostate Cancer Clinical Trials Working Group. J Clin Oncol 26(7):1148-1159, 2008.

40 Goldman B, LeBlanc M, Crowley J. Interim futility analysis with intermediate endpoints. Clin Trials 5(1):14-22, 2008.

41 Yost SE, Smith EN, Schwab RB, et al. Identification of high-confidence somatic mutations in whole genome sequence of formalin-fixed breast cancer specimens. Nucleic Acids Res 40: e107, 2012.

42 Ruczinski I, Kooperberg C, LeBlanc M. Logic regression. J of Computational and Graphical Statistics 12:475-511, 2003.

43 Hussain M, Tangen CM, Higano C, et al. Absolute prostate-specific antigen value after androgen deprivation is a strong independent predictor of survival in new metastatic prostate cancer: data from Southwest Oncology Group Trial 9346 (INT-0162). J Clin Oncol 24: 3984-90, 2006.

44 Tibshirani R. Regression shrinkage and selection via the Lasso. J R Statist Soc B 58: 267-88, 1996.

45 Trapnell C, Pachter L, Salzberg SL. TopHat: discovering splice junctions with RNA-Seq. Bioinformatics 25: 1105-1111, 2009.

SWOG Authorized Document

18.0 APPENDIX

18.1 Intake Calendar

18.2 Translational Medicine Studies

18.3 Instructions for blood sample processing, packaging, and shipping for circulating tumor cell (CTC) analysis in **S1216**

18.4 New York Heart Association Classifications

18.5 Specimen Supplement Sheet

SWOG Authorized Document

18.1 Intake Calendar

SWOG Patient ID________ Patient Initials (L, F, M) ________ **SWOG Study #**__________

Institution/Affiliate ________________________ Physician ______________________________

Instructions for the participant:

This is a monthly calendar on which you are to record the number of tablets/pills/capsules you take each day. Be sure you have enough calendars to last until your next appointment. If you develop any side effects from the tablets/pills/capsules, mark this on the calendar on the day you note the effect. Bring your calendars with you each time you have an appointment.

If you have questions contact: ____________________ Telephone: _____________________

Your next appointment is: _______________________

Special instructions:

Month: **Year:**

Sunday	Monday	Tuesday	Wednesday	Thursday	Friday	Saturday

Patient Signature:_____

18.2 Translational Medicine Studies

a. Markers of bone turnover in newly-diagnosed metastatic prostate cancer patients treated with TAK700: validation of prognostic and predictive value.

Correlative Study Design:

With the patient's consent, serial plasma specimens will be submitted for the proposed correlative studies. Blood specimens will be requested pre-study (after registration) and at Month 7. Blood (plasma) samples will be collected using standard venipuncture techniques. 15 mL of whole blood will be drawn pre- study (after registration, but prior to receiving the first dose of protocol therapy) and subsequently on day one of Month 7 of protocol treatment. This will include patients who have already started androgen deprivation therapy and are eligible for the study. Whole blood will be collected in lavender Vacutainer® tubes and allowed to clot for approximately 30 minutes. Plasma will be separated from cells within 45-60 minutes of venipuncture. Plasma is to be separated from clotted blood by centrifugation at 3,000 x for 10 minutes. Using a pipette, plasma will then equally aliquoted into plastic storage/transport or cryotubes, and shipped according to the instructions provided in the protocol document.

Plasma biomarkers for bone resorption [N-telopeptide (NTX) & Pyridinoline, (PYD)] & formation (C-terminal collagen propeptide, CICP & bone alkaline phosphatase, BAP) will be measured using commercially available assays from Quidel (PYD, CICP, BAP) & Wampole (NTX). Quality control measures will be observed for all plasma specimens collected as part of this proposed trial.

Specific Hypothesis:

Bone metabolism is distinguished by two opposing activities - the formation of new bone by osteoblasts, and the resorption of old bone by osteoclasts. These processes are tightly coupled in space and time. Ultimately, bone mass is dependent upon the balance between formation and resorption. In prostate cancer, this homeostatic balance tips, such that osteoblastic activity predominates, resulting in sclerotic bone metastases. Bone metastasis is a very common event in patients with prostate cancer and is a frequent source of morbidity, including bone pain or fracture. Many prostate cancer patients with bone metastases have elevated circulating biochemical markers of bone metabolism, including markers for osteoblast and osteoclast activity. These plasma-based biomarkers have been explored as indicators of bone turnover for their potential as prognostic and/or predictive variables. *(1)* Prior studies, including our own, have suggested that elevated markers of bone turnover are strongly prognostic for poor survival in castration resistant prostate cancer patients. *(2)* However, their prognostic role in newly diagnosed (hormone-naïve) prostate cancer and their predictive value to help select those most likely to benefit from systemic therapy remain vexing clinical questions. *(3,4,5)*

SWOG **S0421** was a large placebo-controlled Phase III study of docetaxel with or without the endothelin antagonist atrasentan in castration resistant prostate cancer patients with skeletal metastases. Disappointingly, no overall survival benefit was observed for the docetaxel/atrasentan arm. However, as a translational science component of that randomized trial (funded through an R01

award), our group prospectively assessed pre-treatment and serial plasma markers of bone turnover to validate their prognostic and predictive value in these castration-resistant patients. We measured markers of bone resorption (N-telopeptide, NTX & Pyridinoline, PYD) and bone formation (C-terminalcollagen propeptide, CICP & bone alkaline phosphatase, BAP). Cox regression models were developed for overall survival based on baseline BMB (log-2 scale), adjusted for potentially confounding clinical variables (including PSA, bisphosphonate use, age, race, performance status, Gleason score, and pain score, among others). We also explored the effect of treatment on bone marker levels in week 9 (as compared to baseline) and sought to find an association between a change in marker levels and overall survival outcomes. A Cox model was fit with main effects and Bone Marker x Treatment interaction; adjusted for clinical variables, to assess the predictive value of atrasentan on overall survival. Of 1,038 patients enrolled in SWOG **S0421**, 855 (91%) had baseline plasma submitted, of which 778 samples (75%) were usable and subsequently analyzed. The bone turnover values (median; range) were as follows: NTX (14 nM; 9.3-23.9), BAP (64.7 u/L; 35-164), CICP (9.5 ng/mL; 6.5-17.4), & PYD (2.8 nmol/L; 2.2-3.9).

The **significant prognostic role** of elevated markers of bone turnover (> median value) on overall survival is shown in Table 1 below. Hazard ratios are reported for a 2-fold increase in markers. Follow-up analysis revealed that patients with high marker levels (ie, upper 25%-ile across all markers) not only have a **very poor prognosis** (HR = 4.3, p <0.001) but also appear to have a **significant survival benefit from atrasentan** (interaction-HR=0.33; p=0.002), even with highly conservative statistical analysis including Bonferroni adjustment. There were 6% (47) of the 778 patients with a complete set of measurements on baseline markers. The HR in the high marker group was estimated at 0.34 (95% CI: 0.13, 0.89); this compares with a HR of 1.04 (95% CI: 0.86, 1.25) in the low marker group.

**Table 1: Prognostic Value of Markers of Bone Turnover**			
**Biomarker**	**Hazard Ratio** (95% CI)	**Median Survival, months** (<= median BMB value vs. > median)	**p-value***
BAP (u/l)	1.26 (1.19, 1.35)	22.8 vs. 15.4	<0.001
CICP (ng/ml)	1.38 (1.27, 1.5)	24.5 vs. 14.6	<0.001
NTx (nM)	1.43 (1.31, 1.57)	22.4 vs. 15.5	<0.001
PYD (nmol/l)	1.49 (1.32, 1.7)	20.3 vs. 15.3	<0.001
*Significance was set at ≤ 0.006 (Bonferroni adjustment to control overall 2-sided error rate across 4 tests at 0.05).			

There was also strong evidence for a significant association between the change in bone marker concentrations from baseline to Week 9 and overall survival for all bone markers (Table 2).

**Table 2: Association between overall survival and the change from baseline to week 9 in the (log-2) bone marker concentration, adjusted for baseline bone marker concentration and bisphosphonate use**			
**Marker**	**Hazard Ratio**	**95% CI**	**P-value**
BAP (u/l)	1.33	(1.16, 1.51)	<0.001

SWOG Authorized Document

CICP (ng/ml)	1.46	(1.23, 1.72)	<0.001
NTx (nM)	1.45	(1.2, 1.75)	<0.001
PYD (nmol/l)	1.59	(1.25, 2.02)	<0.001

These results demonstrate the ability of our group to successfully complete biomarker driven correlative studies in the context of a large Phase III trial. We have also definitively established that baseline plasma levels of bone turnover markers have significant independent overall survival prognostic value in patients with castration resistant disease. In addition, we found that a small subset of high-risk patients with elevated plasma bone turnover levels appear to preferentially benefit from a novel systemic agent such as atrasentan.

Presently, it is unclear whether these same observations will be observed in a somewhat different patient context: i.e., in the newly diagnosed, metastatic prostate cancer setting (non-castrate resistant). This proposed Phase III trial (S1216) of a LHRH agonist + bicalutamide versus a LHRH agonist + TAK-700 provides a unique opportunity to directly address this important clinical question.

As noted above, TAK-700 is a novel CYP-17 inhibitor. It is notable that certain polymorphisms in the CYP-17 gene have been reported to modulate bone metabolism and turnover, and have been implicated in osteoporosis development. *(6)* TAK-700 selectively inhibits 17,20 lyase, a critical enzyme involved in the steroidogenesis and androgenesis pathways. An inherited defect in any of the five enzymatic steps – including 17,20 lyase - needed for cortisol synthesis results in a clinical condition called congenital adrenal hyperplasia (CAH). *(7)* This condition is characterized by ambiguous external genitalia, advanced bone age, and early closure of epiphyses, among others. Interestingly, it has been reported that *bone turnover is actually decreased in adult patients with CAH*, and that plasma or urine markers of bone turnover (including NTX, BAP, and osteocalcin) are significantly lower in these patients compared to normal subjects. *(8)* These data support the notion that inhibition of CYP17 or 17, 20 lyase will result in modulation of bone metabolism and turnover.

The encouraging results from our own SWOG **S0421** bone marker studies in CRPC and the association between bone metabolism and CYP-17 led us to pose several clinically relevant questions that can be prospectively addressed in **S1216**:

- Are markers of bone metabolism of prognostic and predictive value only in the CRPC setting, or do they have the same value in newly diagnosed metastatic prostate cancer?
- Do markers of bone turnover prognosticate only when radiographic bone metastases are present?
- Will TAK-700 therapy result in a bone biomarker phenotype reminiscent of CAH?
- How will this TAK-700-induced biochemical CAH phenotype behave in terms of response and survival when compared to a control group that is not receiving TAK 700?
- Can we use bone turnover biomarkers to predict who will benefit from CYP17 inhibitor therapy?

SWOG Authorized Document

We therefore **hypothesize** that baseline levels of bone metabolism markers in sera collected from patients with newly diagnosed metastatic prostate cancer will be of strong prognostic value and will also be predictive of benefit from the CYP17-inhibitor TAK-700 in combination with androgen deprivation therapy. A secondary hypothesis is that serial assessment of these same markers (at baseline and again at Month 7) will demonstrate that lower marker levels in Month 7 will be associated with improved survival.

These will be tested through the following **Specific Aims**:

1. **To measure levels of selected markers of bone formation (C-terminal collagen propeptide, CICP and bone alkaline phosphatase, BAP) and resorption (N-telopeptide, pyridinoline) in sera collected at baseline and at month 7 in patients enrolled in this proposed randomized Phase III trial of LHRH agonist + TAK-700 versus LHRH agonist + bicalutamide in patients with newly diagnosed patients with metastatic prostate cancer.**

2. **To correlate the results of these marker studies with tumor response, progression-free survival, and overall survival to detect prognostic (baseline or pre-treatment sera) and/or predictive value (baseline biomarker x treatment interaction; serial sera).**

3. **To identify prognostic groups based on baseline bone markers and other clinical and disease-related factors.**

We anticipate that the results of this bone marker study will improve the ability of oncologists to appropriately select patients with newly diagnosed metastatic prostate cancer who are most likely to benefit from TAK-700 therapy, exclusive of other clinical variables. In addition, these results will validate the potential prognostic value of these markers in this patient population, and therefore assist clinicians in counseling patients about their anticipated long term outcome, regardless of treatment received. The randomized nature of this proposed Phase III trial allows us a unique opportunity to address these critical issues. We plan on submitting an R01 (or equivalent) grant application to support these translational studies.

b. Genomic variants and gene expression of androgen pathway genes

Background

For decades, androgen deprivation therapy with a luteinizing hormone releasing hormone (LHRH) agonist and an antiandrogen has been the cornerstone of metastatic prostate cancer (mPC) treatment. (9) Although initially effective, resistance to therapy typically heralds a critical downturn for patients. Potent new hormonal therapies such as abiraterone and enzalutamide are being developed to target the androgen pathway in mPC, yet early experience with these agents has demonstrated heterogeneity of clinical response. (10,11) As these and other new agents enter clinical study, it is increasingly important to establish their optimal role and identify patients most likely to benefit from their use over standard treatment. Yet, to date no such effort has been undertaken to identify and validate robust biomarkers of response to hormonal therapy in a large prospective clinical cohort.

SWOG Authorized Document

Recent studies by our group and others suggest that response to androgen deprivation is associated with genetic variants as well as transcript levels of some androgen pathway genes. (12,13,14,15,16,17) Based on these insights, our multi-investigator team now proposes to identify predictive biomarkers within the androgen pathway in a comprehensive manner encompassing the genetic, biological, and clinical aspects of mPC as it evolves over time. This study will be conducted in the context of **S1216**, a large (1,500 men), randomized Phase III clinical trial led by the Southwest Oncology (SWOG) NCI Cooperative Group, in which men with hormone-naïve mPC will receive leuprolide (LHRH agonist) combined with either bicalutamide (androgen receptor antagonist) or orteronel (TAK-700, a novel selective CYP17,20 lyase inhibitor). **We hypothesize that initial response and subsequent resistance to androgen deprivation therapy in S1216 will be associated with genetic sequence variants and differential expression of genes within the androgen pathway**. We will identify and validate these predictive markers by analyzing host and tumor tissue and clinical outcomes in CLIA-certified settings as follows:

Aim 1: We will analyze androgen pathway genes in **host cells** to identify germline sequence variants associated with response to therapy.

a. DNA will be extracted from white blood cells of mPC patients in **S1216**, and androgen pathway genes will be analyzed in these samples using targeted sequencing.
b. Sequence variants will be correlated with response to therapy (PSA at 7 months and time to castration resistance) to determine which variants are associated with clinical outcome, first across both treatment groups, and subsequently by treatment arm.

Aim 2: We will analyze androgen pathway genes in **circulating tumor cells (CTCs)** for somatic sequence variants and mRNA expression levels associated with response to therapy and with castration resistance.

c. At the start of hormonal therapy and at progression to castration resistance, DNA from CTCs of mPC patients in **S1216** will be analyzed by targeted sequencing to detect somatic sequence variants associated with disease response and with castration resistance.
d. At both time points, mRNA from CTCs will be analyzed using Open Array RT-PCR to identify androgen pathway genes whose transcript levels are associated with response and with castration resistance.
e. As an exploratory sub-aim, whole transcriptomes from CTCs of 10 responders and 10 non-responders will be amplified and sequenced to discover novel differentially expressed gene candidates associated with response and resistance to hormonal therapy.

Aim 3: We will analyze androgen pathway genes in **primary tumor cells** for somatic sequence variants and mRNA expression levels associated with response to therapy and will also compare primary tumor androgen pathway gene profiles to those from advanced metastatic disease (CTCs) in the same patients.

a. Prostatectomy specimens from mPC patients in **S1216** (available in a subset of participants) will be microdissected, and DNA will be extracted for targeted sequencing to detect somatic sequence variants associated with disease response; variants will also be compared with somatic variants in CTCs from the same subjects (Aim 2).
b. mRNA from microdissected prostatectomy specimens will be analyzed using Open Array RT-PCR to identify androgen pathway genes whose transcript levels are associated with response; transcript levels will also be compared

to those measured in CTCs from the same subjects.

c. As an exploratory sub-aim, whole transcriptomes from microdissected prostatectomy specimens of 10 responders and 10 non-responders will be amplified and sequenced to discover novel differentially-expressed gene candidates associated with response and resistance to hormonal therapy.

Our multi-investigator team is uniquely positioned to conduct these studies given our broad expertise in the genetic, biological, and clinical facets of prostate cancer as well as our experience with advanced technologies for high throughput sequencing and for CTC capture and analysis in CLIA-certified settings. Most importantly, this large SWOG-led Phase III randomized study utilizing both standard and novel hormonal agents will offer a unique opportunity to identify and validate urgently needed new biomarkers for more effective and personalized hormonal treatment of mPC.

Research Strategy

Significance. Prostate cancer (PC) is the most prevalent and the second most lethal cancer among American men. *(18)* For men with metastatic PC (mPC), androgen deprivation therapy (ADT) with a luteinizing hormone-releasing hormone (LHRH) agonist combined with an androgen receptor (AR) antagonist is the mainstay of treatment. *(19)* While initially effective, the duration of benefit is highly variable, ranging from a few months to several years, and it is difficult to reliably predict which men will benefit and for how long. *(20)* Eventually, mPC progresses to a castrate-resistant state, though androgen-signaling networks remain vitally active in castration-resistant PC (CRPC). *(21)* These networks can be triggered by androgens produced in the adrenal glands and in the tumor itself. In fact, enzymes involved in androgen synthesis are upregulated in CRPC cells, and androgen pathways have emerged as rational therapeutic targets in CRPC. *(22)* Several potent agents targeting this network have been developed, including new AR antagonists such as enzalutamide, and agents targeting androgen synthesis via the CYP17 pathway, such as abiraterone, both of which have improved survival in CRPC. *(23,24,25,26,27,28)* Orteronel (TAK-700) is a novel inhibitor of the CYP17 pathway that is highly selective for CYP17, 20 lyase. This agent has shown promising activity in patients with metastatic CRPC as well as patients with biochemical recurrence, and it may lack some of the common toxicities of CYP17 hydroxylase inhibitors. *(29,30)*

Early experience with these newer hormonal agents has shown their promising therapeutic potential but has also demonstrated, as with traditional hormonal therapies, that there are subgroups of patients more or less likely to respond. *(31)* Currently, there are no reliable biomarkers that predict response to various hormonal agents; hormonal therapy remains empiric and there are few personalized treatment strategies in mPC. Therefore, as the hormonal armamentarium expands, it is increasingly important to establish the optimal use of new agents and identify patients most likely to benefit from their use over standard treatment. The key to identifying predictors of clinical response lies in the biological interaction between ADT and the host and tumor androgenic milieu. For example, germline variations in some androgen pathway genes are associated with response to ADT and castration resistance. *(32,33,34,35,36,37)* Moreover, transcript levels of AR and several steroidogenic enzymes vary significantly in CRPC cells, and resistance to CYP17 inhibitors and AR antagonists was shown to occur through upregulation of CYP17A1 and/or induction of AR and AR splice variants that confer ligand-independent AR transactivation. *(38,39,40,41)* In our own work, we demonstrated a direct effect of luteinizing hormone (LH) and LHRH on androgen synthesis in PC cells, and

SWOG Authorized Document

we found that inherited variants in androgen pathway genes correlate with ADT response in mPC and with PC survival. *(42,43)*

These intriguing preliminary findings suggest that patient-specific variations in androgen pathway genes indeed contribute to susceptibility and resistance to hormonal therapies. However, there has been no comprehensive analysis of these pathways and their effect on clinical outcome in a large prospective cohort. As importantly, the interplay between germline and somatic variants, gene expression, and response to therapy has yet to be elucidated. To address these questions, we propose to test how genes within the androgen pathway govern initial response and subsequent resistance to ADT in **S1216**, a Phase III SWOG-sponsored prospective clinical trial of 1,500 men with hormone-sensitive prostate cancer (HSPC) initiating treatment with leuprolide and either bicalutamide or orteronel. Our multi-investigator team will use high throughput targeted sequencing and gene expression to analyze white blood cells (WBC, Aim 1), circulating tumor cells (CTC, Aim 2), and matched primary tumors (Aim 3) from the 1,500 patients in **S1216** in order to identify and validate androgen pathway genes whose sequence variants or expression levels predict response and eventual resistance to ADT. Additionally, in an exploratory subset of responders and non-responders, whole transcriptomes will be sequenced to identify novel gene candidates and regulatory elements that drive response to ADT. These analyses will yield a comprehensive picture of how clinical response to hormonal therapy is associated with variations in androgen pathway biology in the host (Aim 1), CTCs (Aim 2), and the primary tumor (Aim 3) as the disease progresses from HSPC to CRPC, promoting more rational and mechanism-driven use of hormonal agents and ultimately more personalized and effective treatment.

Innovation

Our proposal brings together several innovative strengths:

Unique SWOG trial opportunity: Large prospective trials in hormone sensitive prostate cancer are quite rare due to the large required cohort size and clinical follow-up. Our proposal takes advantage of a unique window of opportunity: **S1216** is a large, randomized, multi-center Phase III clinical trial of hormonal therapy for mPC in a cohort of nearly 1,500 men, in which disease stage, therapy response, and adverse events will be uniformly prescribed and assessed. Moreover, roughly 20% of patients will be of a racial minority based on prior SWOG accrual patterns. The trial has just opened and will enable us to identify new markers of response to hormonal therapy, advancing precision medicine for the millions of men treated annually with these agents.

Clinically and biologically important endpoints: Clinically, the design and large cohort size of this study will enable not only discovery but also validation of predictive markers, in samples from the 1,500 participants, laying the groundwork for subsequent therapeutic trials that will prospectively use these biomarkers for patient selection or stratification. Biologically, it will help to elucidate mechanisms of PC progression by analyzing androgen gene sequence and expression as the disease evolves over time, from host (WBCs) → localized disease (primary tumor) → hormone-sensitive mPC (CTCs) → castration-resistant mPC (CTCs).

Use of a novel hormonal agent: These studies will yield predictive markers and biological insights about response and resistance not only to established agents, but also to orteronel, a new CYP17, 20 lyase inhibitor.

Experienced multi-specialty team: Our team of investigators has collaborated successfully in prior R01-funded large biomarker studies (SWOG **S0421**) and

SWOG Authorized Document

has the broad expertise and experience necessary to undertake and complete the proposed work: Goldkorn (oncology, CTCs); Pinski (oncology, prostate endocrinology); Ingles (prostate genomics); Triche (biomarkers, next generation sequencing); Tangen (SWOG biostatistics); Agarwal and Quinn (prostate clinical trials); Li and Chen (bioinformatics).

High throughput sequencing: The most common approach to identifying germline genetic variations has been the use of pre-configured single nucleotide polymorphism (SNP) arrays comprised of candidate and/or tag SNPs, a strategy that misses numerous genomic features of potential importance. In this project, we will instead use high throughput deep sequencing of genomic regions of interest, allowing detection of rare genetic variants, insertions and deletions, copy number variations (CNVs) and other structural variants in the context of a CLIA certified, translational genomics core suitable for clinical samples. This approach will be complemented by gene expression analyses using the highly multiplexed Open Array qRT-PCR platform in all samples, as well as whole transcriptome profiling (RNAseq) in a subset of patient responders and non-responders.

Advanced CTC capture and analysis platforms: CTC studies to date have focused either on simple enumeration of CTCs or on measurement of one or two markers, not fully capitalizing on the potential of CTCs as a real-time tumor tissue source in advanced disease. In this study, CTCs will be extensively characterized using advanced, highly effective techniques for enrichment, amplification, and high-throughput analysis. Technologies such as the CLIA-certified LiquidBiopsy platform, the Parylene-C slot microfilter, the Open Array qPCR, and the AmpliSeq targeted sequencing have been extensively used and indeed co-developed by members of our team for CTC analyses in pilot studies and clinical trials.

Approach

Clinical Design of Parent Trial: The patient population will be enrolled in a prospective, multi-center, randomized Phase III, CTEP-approved SWOG trial (**S1216**) with co-investigator Dr. Neeraj Agarwal as the clinical trial's Principal Investigator. This trial will accrue 1486 men with hormone-naïve mPC from SWOG-participating sites (> 500 institutions). Based on our prior experience in this population (**S9346**), we estimate accrual of 28 patients per month lasting just over 4 years. After providing informed consent, these men will be treated with the LHRH agonist leuprolide continued indefinitely per current standard of care. Patients randomized to standard therapy will receive bicalutamide 50 mg by mouth daily and those randomized to the experimental arm will receive orteronel 300 mg by mouth twice daily. PSA will be measured monthly for 4 months then every 3 months until progression. As a quality control measure, testosterone levels will be checked every 3 months, helping to minimize the chance that associations emerge due to poor adherence to therapy.

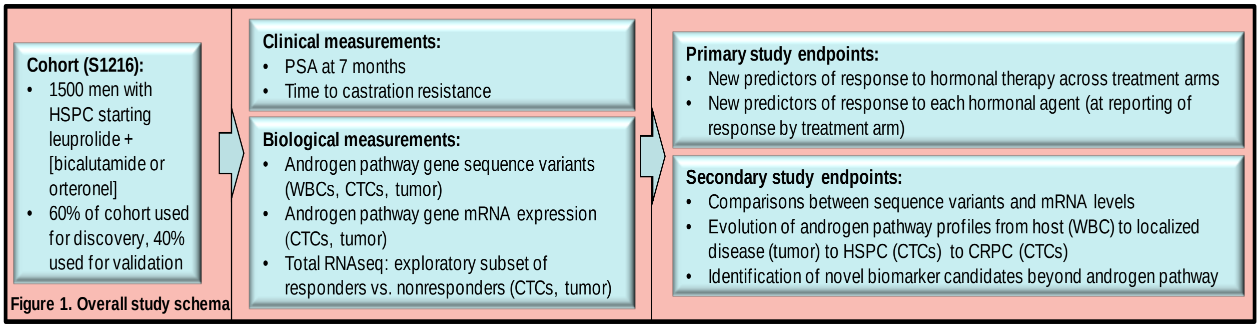

Figure 1. Overall study schema

SWOG Authorized Document

Clinical Endpoints of the Proposed Correlative Studies
The following clinical endpoints from the parent trial (**S1216**) will be used for the proposed project (Figure 1):

1. *PSA value after 7 months of therapy.* Reports from **SWOG-9346** by Hussain et al demonstrated that a PSA value of 4 ng/ml or less after 7 months of androgen deprivation is a strong predictor of survival. *(44)*
2. *Time to castration resistance.* Reports from **SWOG-9346** by Hussain et al also demonstrated that time to castration resistance is a strong predictor of survival. *(45)* Time to castration resistance will be defined as the time from the date of registration to documentation of progression by RECIST 1.1 criteria, PSA criteria (≥ 25% increase from baseline PSA and an absolute increase of ≥ 2 ng/ml from nadir PSA), development of ≥ 2 new lesions on bone scan (confirmed on a subsequent bone scan), symptomatic deterioration requiring discontinuation of therapy or death due to any cause.

Overall Rationale for Androgen Pathway Gene Candidates
We have reported that multiple androgen synthesis enzymes are up or downregulated in PC cells exposed to LH or LHRH, suggesting that hormonal therapies may induce similar adaptive changes in our patients' tumors. *(46)* These data are corroborated by our finding that SNPs in LHRH and LH receptor (LHR) are associated with clinical outcomes in PC. *(47)* Indeed, other groups have recently reported that several variants in androgen biosynthesis enzymes are associated with androgen response and clinical outcomes. *(45,46,53,58)* Similarly, it has been shown recently that truncated AR splice variants that have intact DNA binding and transactivation domains but lack the ligand-binding domain (AR-V proteins) can mediate therapy resistance *in vitro*. *(48)* Based on these collective rationale, we have compiled a comprehensive list of 46 androgen pathway genes (Table 1) that will be analyzed in all aims of this project. These include hypothalamic and pituitary hormones and their receptors, enzymes involved in androgen metabolism and biosynthesis, Phase I and Phase II inactivation, and androgen binding and uptake.

Table 1: List of Androgen Metabolism and Signaling Genes to Be Interrogated

AKR1C1	*AKR1C2*	*AKR1C3*	*AKR1C4*	*AR*	*CYB5*	*CYP11A1*	*CYP11B1*	*CYP17A1*	*CYP19A1*
CYP21A2	*CYP3A4*	*CYP3A43*	*CYP3A5*	*CYP3A7*	*HSD17B1*	*HSD17B10*	*HSD17B11*	*HSD17B12*	*HSD17B13*
HSD17B14	*HSD17B2*	*HSD17B3*	*HSD17B4*	*HSD17B6*	*HSD17B7*	*HSD17B8*	*HSD3B1*	*HSD3B2*	*HSD3B7*
LH	*LHR*	*LHRH*	*LHRHR*	*RDH5*	*SHBG*	*SLCO1B3*	*SLCO2B1*	*SRD5A1*	*SRD5A2*
SRD5A3	*STAR*	*TRMT11*	*UGT2B15*	*UGT2B17*	*UGT2B7*				

SWOG Authorized Document

<u>Aim 1</u>: Analyze germline (WBC) androgen pathway genes for variants associated with ADT response.

Rationale

Germline variations have been extensively documented to associate with cancer susceptibility and more recently with cancer outcomes (e.g. SNPs in 3b-HSD1

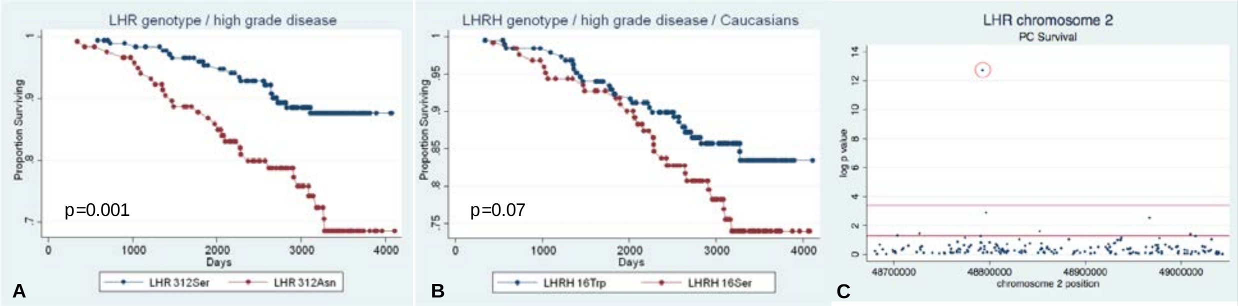

Figure 2. SNPs associated with PC specific survival. A. Association with LHR genotype. **B.** Association with LHRH genotype. **C.** p-values for association between LHR SNPs and PC survival in African American patients.

and HSD17B2 associated with castration resistance and PSA recurrence, respectively). *(49,50,51,52)* Our own studies recently identified variants in the LHR and LHRH genes that predict response to LHRH agonist therapy and survival among men enrolled in a case-control study of PC conducted by Dr. Ingles (Figure 2). *(53)* We investigated these genes in more detail in a subset of 486 African American PC cases and found 9 SNPs in the LHR gene significantly associated with survival at the 0.05 level, among which one survived correction for multiple hypothesis testing, with a $p=2x10^{-13}$ (Figure 2C). In the LHRH gene, 4 SNPs were associated with PC survival at the 0.05 level of significance (data not shown).

In a separate study, presented at the 2011 ASCO Annual Meeting, we measured LHR genotype and testosterone levels for 47 men who had received ADT with an LHRH agonist for advanced PC under our care. Men carrying 1 or 2 (vs. 0) LHR 312 minor alleles had a shorter time to castration resistance. Moreover, peak testosterone (Figure 3A) and nadir testosterone (not shown) were both higher for men with a minor allele at position 312 (Figure 3B). *(54)* Overall in this study, LHR 312 minor allele carriers experienced an inferior castration response to LHRH agonist therapy and a shorter duration of response to therapy. Collectively, these data support a role for LHR and LHRH germline variants in PC outcomes. Therefore, in Aim 1 we propose a more comprehensive interrogation of these genes and other androgen pathway genes to evaluate an association between gene variants and clinical endpoints in men treated with hormone therapy.

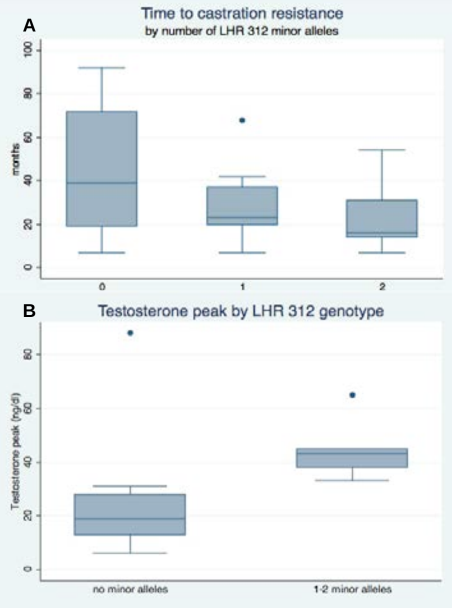

Figure 3. LHR Genotypes. A. Time to castration resistance (months) for men with 0, 1 or 2 minor alleles at LHR 312. **B.** Peak testosterone (ng/dL) for men with 0 vs. at least 1 minor allele.

SWOG Authorized Document

Design

Targeted sequencing of androgen pathway genes. Upon enrollment, a 7.5 ml sample of peripheral blood will be drawn from each patient and shipped at room temperature to the SWOG specimen processing center at Nationwide Children's Hospital Biopathology Center in Columbus, OH. Aliquots of frozen buffy coat will be shipped to USC for DNA extraction and sequencing at the Genomics Core at USC/CHLA (Figure 4). To analyze androgen pathway gene variants, we will take advantage of the massive throughput capabilities of Next Generation (NextGen) sequencers and sequence 1.5 Mb of targeted genomic regions. We have targeted the entire region surrounding each of the 46 selected genes, including introns and upstream & downstream regions that may harbor regulatory elements. The sequencing strategy we have proposed overcomes many limitations of genome-wide association (GWA) studies that historically relied on haplotype tagging SNPs to identify regions harboring common genetic variants associated with disease. Comprehensive targeted sequencing will allow us to detect not only SNPs, but also other types of variation, including insertions and deletions, CNVs and structural variants. Most importantly, we will be able to identify variants too rare to be picked up by GWA but which may have relatively large functional effects. *(55,56)*

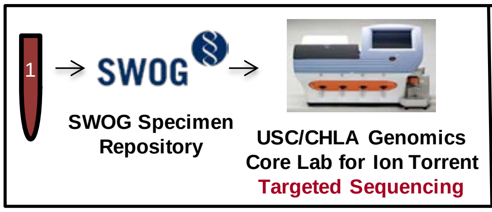

Figure 4. Aim 1 Schema. One 7.5 ml sample will be sent to the SWOG specimen processing center. Aliquots of buffy coat will be sent to the CHLA Genomics core for sequencing.

SWOG Authorized Document

All targeted sequencing for this proposal will be conducted at the USC Children's Hospital LA (USC/CHLA) Genomics Core (Figure 5), a CLIA certified resource created in 1999 by Dr. Triche (co-investigator) which has since processed over 30,000 microarrays of diverse type, performed thousands of Sanger sequences,

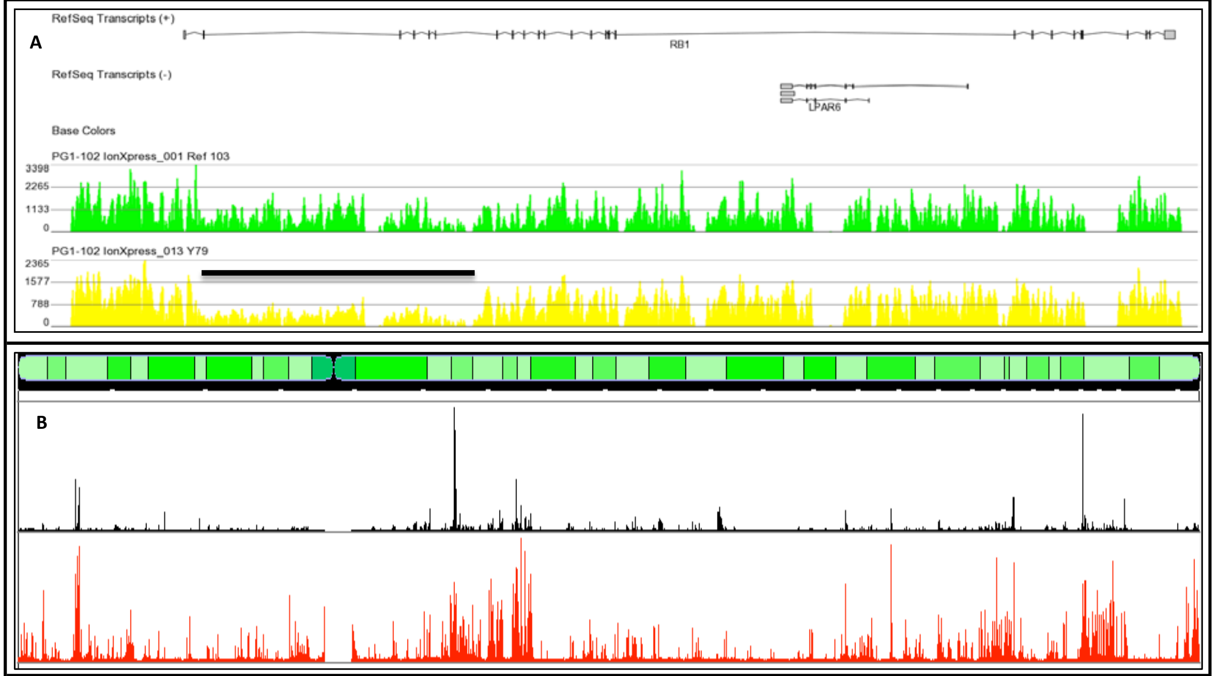

Figure 5. Targeted and genome-wide sequencing. A. Targeted sequencing of RB1 in WBCs (green) vs. tumor (yellow) with heterozygous loss of exons 2-6 (line). B. Genome-wide total RNAseq of tumor (black) and overlaid ChIPseq for H3K4Me3 marks (red). Only chromosome 12 is shown to allow greater image resolution, though a similar pattern is preserved across the genome.

and more recently, implemented NextGen sequencing on the Ion Torrent Personal Genome Machine and the Proton sequencer. Over 100 whole exomes, >100 whole genome methylation analyses, and >200 RNAseqs have been performed. Most recently, targeted methods have been developed for DNA sequencing of genes (RB1, AmpliSeq panel, MYCN, etc.), along with methods to maximize percent of target region covered: 100% exon coverage and 97% total coverage have been achieved for the RB1 gene, for example. Similar optimization is performed for any given target gene or locus. Finally, a comprehensive bioinformatics analytic pipeline, from raw read alignment to biologic integration, has been developed in both software (e.g. Genetrix) and among bioinformatic personnel. Additional targeted sequencing and analytic methods (e.g. sequencing of non-coding RNA/protein/DNA complexes: "ChIRP Seq") are in continual development.

For the current study, targeted sequencing steps will include: 1) generation of amplicons of targeted regions for sequencing using Haloplex, AmpliSeq (LifeTech, Inc.), custom long-range PCR primers, or TargetSeq (LifeTech, Inc.) capture of whole-genome amplified DNA, as determined by the nature of the target regions; all have been successfully employed by the core for targeted sequencing with 100% coverage of the target region. Coverage considerations determine the method of choice; 2) ligation of bar-code adapters to each sample; 3) preparation of 100, 200, 300, or 400 base insert size libraries, depending on coverage requirements; 4) sequencing on the Ion Torrent PGM analyzer with 314, 316, or 318 chips, or on the Proton 1 chip on the Proton analyzer as

SWOG Authorized Document

dictated by depth of coverage required, sample numbers and batch size. Choice of chip and sequencer will be determined to achieve a read depth of at least 100X at each nucleotide for each sample in each batch. Batches will be run weekly to minimize cost yet achieve reasonable turnaround.

Bioinformatics. FASTQ files generated by the analyzer will be aligned using BMA or related aligners to create BAM files for analysis. Sequence quality and alignment will be confirmed with standard quality control measures such as Q score > 20 and subsequently, SNPs will be identified using Ion Reporter, Partek, or Genetrix software. Importantly, methods for identifying SVs, CNVs and InDels from high-throughput sequence data are rapidly evolving. Typically, these methods rely on evaluating depth of pile-ups of 100-400 base sequence reads against the reference genome. Although first generation "depth of coverage" methods can suffer from low sensitivity and specificity, a number of algorithm refinements have been recently implemented to improve performance, e.g. CNVnator and ReadDepth. (64-65) Particularly relevant to our proposed study, Nord et al have described a robust, easy to implement algorithm for combining depth of coverage with analysis of partially mapped reads to detect CNVs and estimate copy number from samples that have been enriched for targeted genomic regions (such as the androgen pathway enrichment proposed in our study). (57) Most recently, Wu et al have provided a Bayesian algorithm that eliminates the need for matched normal samples for read depth normalization to detect CNVs in exon capture sequencing. (58)

Treatment of Genotypes for Statistical Analysis. Genotypes will be examined in several ways. First, we will take a candidate SNP approach, examining a limited number of polymorphisms of documented significance. The major advantage of analyzing these SNPs separately is higher power in the event that one of these SNPs is indeed a causal locus. Secondly, we will examine all common variants (SNPs and CNVs), regardless of known function. Even though this approach results in a number of hypotheses being tested, they are not independent and thus full Bonferroni correction for multiple comparisons is not required. We will apply the Bonferroni correction to the effective number of independent tests, determined by principal components analysis (the "Simple-M" method), to take into account correlation between the variants. *(59)* Finally, we will examine the spectrum of genetic variation (including rare variants) revealed by sequencing. One approach will be mutation-accumulation analysis, which compares the number of observed rare alleles among men with different treatment outcomes. This technique has the potential to reveal an excess of rare variants among men at opposite ends of the phenotypic spectrum. *(60)* However it cannot address the impact of specific variants or combinations of variants. To more closely assess the similarity of DNA sequences among study participants and to relate sequence similarity to treatment outcomes, we will employ the method of multivariate distance matrix regression. *(61)*

Expected Results, Potential Pitfalls, and Alternative Approaches
We will examine associations between clinical outcomes (PSA value after 7 months of therapy and time to castration resistance) and germline (host) genetic variations in androgen pathway genes. We note that various other genes and pathways (e.g. c-myc, PI3K, 8q loci) have been reported as associated with clinical outcomes in PC, but in order to preserve a prospective, hypothesis-driven (androgen pathway) focus, these were not included in the list of genes in Table 1. Similarly, we recognize that epigenetic mechanisms (e.g. non-coding RNA) also may play a role. These potential candidates will be addressed in exploratory

SWOG Authorized Document

Aims 2C and 3C of this proposal, in which RNAseq will be used to compare whole transcriptomes from responders vs. non-responders. Candidates that

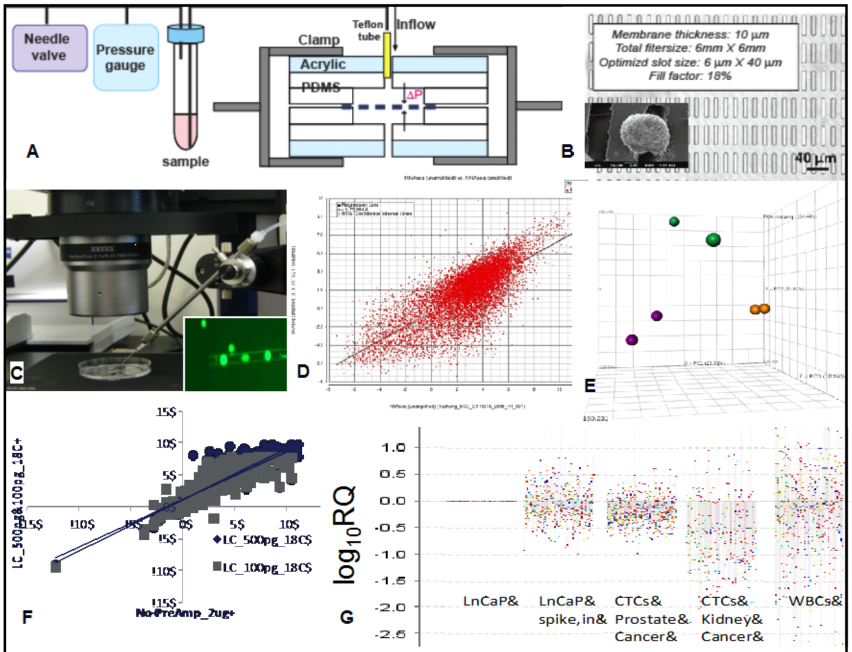

Figure 6. CTC technologies developed and used by our team. A. Filter apparatus. **B.** Slot microfilter with captured cell (SEM) inset. **C.** Motorized micropipette apparatus for single CTC pickup. **D.** Random hexamer linear amplification of total RNA (R^2=0.8) using Nugen Ovation kit. **E.** PCA map of duplicate HiSeq2000 gene expression patterns of ultra-pure CTCs (green) compared to primary tumor cells (orange) vs. metastases (purple) from the same mouse hosts. **F.** Multiplexed PCR pre-amplification (LifeTech) yields R^2=0.71 using 600 cancer gene OpenArray panel. **G.** OpenArray 600 gene expression (from L to R): 25 LNCaP cells, 25 LNCaP cells spiked into blood and retrieved by WBC depletion and micropipette, pure CTCs from a patient with PC, pure CTCs from a patient with kidney cancer, WBC only. Relative quantities (RQs) are normalized to unspiked LNCaP (first column). R^2 =0.87 for unspiked LNCaP vs. spiked and retrieved LNCaP (1st vs. 2nd column).

emerge from those exploratory subaims (and any other genes of interest) can be assayed retrospectively from the stored DNA and RNA samples of the entire cohort.

SWOG has a long track record of minority accrual (approximately 20% expected), which presents both challenges and opportunities. While the prevalence of genetic variants may vary greatly by race, we do not expect that a genetic variant will influence treatment response differently, depending on the patient's race. Yet, we may find that some genetic variants are associated with treatment response among Whites but not African Americans or vice versa. Such heterogeneity may arise when the variant in question is not causally related to the outcome, but is in linkage disequilibrium (LD) with another causal variant and the LD varies by race. To address this issue we will examine haplotypes and LD patterns by racial group. Heterogeneity thus provides us with the opportunity to fine-map correlated genetic variants or narrow down the likely source of a causal association with treatment response. An additional benefit of race-specific analyses will be the possibility of identifying variants prevalent in particular racial groups that may be predictive within that group.

<u>**Aim 2: Ana**</u>lyze androgen pathway genes in CTCs for somatic sequence variants and RNA expression levels associated with response to therapy and castration resistance.

SWOG Authorized Document

Rationale
Capture and analysis of CTCs enables minimally-invasive cancer sampling and real-time tracking of disease biology as it evolves over time. Characterization of CTCs representing advanced disease can complement analysis of primary tumor by offering insights about cancer progression from an early localized state to advanced metastatic disease. *(62,63)* Multiple CTC enrichment and capture technologies have been developed in recent years, based on cell surface markers, size, or electrokinetic properties, and each is attended by unique advantages and disadvantages. *(64,65,66,67)* In PC, CTC enumeration has been shown to provide prognostic and predictive value in advanced disease, and prostate CTCs have been successfully assayed for cancer-specific molecular markers, illustrating the great potential of CTC analysis in the personalized management of PC. *(68,69,70,71,72,73,[74],[75],[76],[77])*

CTC technology
Our group recently developed a Parylene-C slot microfilter which can capture and enrich live CTCs from whole blood rapidly and efficiently based on differential size and deformability (Figure 6A-B). (78) Captured cells can be immunofluorescently stained for cancer-specific protein markers and imaged on the filter using a fully-automated, multi-spectral scanning and imaging platform (Zeiss) specially configured for this purpose. Alternatively, captured cells can be lysed for qPCR analysis, or even picked cell-by-cell using an xyz-motorized micropipette apparatus (Eppendorf) specially configured for this application (Figure 6C). Ultra-pure CTC capture completely eliminates any WBC contamination and enables the type of high throughput analyses described in the current proposal. In several pilot studies, we have used these techniques to pick up ultra-pure (no WBC background) CTCs from human and mouse blood specimens, to extract and amplify their total RNA with high fidelity using either Nugen Ovation random hexamer linear amplification (for total RNA) or LifeTech PreAmp qPCR (for targeted gene panels), and to analyze gene expression using RNAseq or OpenArray platforms, respectively (Figure 6D-G). Other systems in our laboratory include the ClearCell size-based microfluidic CTC platform (Clearbridge, Inc.) and the Stem Cell Technologies immunomagnetic WBC depletion platform. We have also been using the LiquidBiopsy EpCAM-based microfluidic CTC capture platform (Cynvenio, Inc., Westlake Village, CA) to generate CTC genomic data (see below). This technology has been CLIA certified, and a dedicated platform will be placed in our laboratory for collaborative on-site use in the proposed study.

SWOG Authorized Document

CTC Analysis in Clinical Trial *Settings*

To date, we have applied CTC capture and analysis in a variety of clinical trial settings. In mPC, our team proposed and led a large NCI R01 funded project of CTC capture and analysis as part of a North American Intergroup Phase III prospective mCRPC trial, SWOG 0421 (**S0421**). *(79)* As Principal Investigators, our group performed live CTC capture and analysis on over 700 patient blood specimens and also directed the trial (regulatory coordination, specimen shipping, and data collection). As an example of our multi-platform approach,

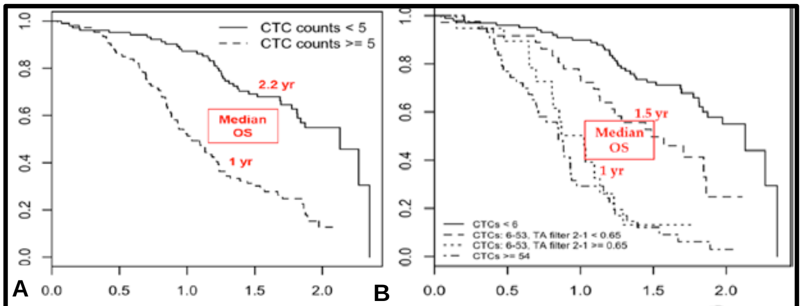

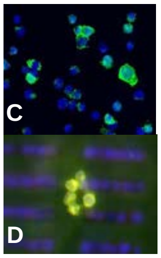

E

	Detection Rate	Transcript Level: Med(Range)
IGF-I	9 (47%)	34.5 (31.2 – 36.7)
IGF-II	10 (53%)	35.9 (1.2 – 39.2)
IGF-IR	5 (26%)	27.2 (25.0 – 32.1)
MET	10 (53%)	37.1 (0.83 – 39.4)
mTOR	8 (42%)	28.9 (26.4 – 35.6)

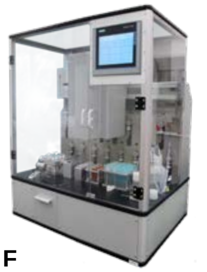

# CTC / 7.5 ml	SNV in CTC (Mut%, Read Depth)	# CTC / 7.5 ml	SNV in CTC (Mut%, Read Depth)
59	IDH1, P118L (2.6%, 662)<br>EGFR, G873R (1.4%, 1053)<br>MET, A179T (2.1%, 12955)<br>RB1, I680Y (1.7%, 1640)<br>JAK3, A127V (11.9%, 697)	217	PIK3CA, I391M (2.0%, 10482)<br>APC, P852S (1.7%, 13495)<br>CSFR1, L309F (1.7%, 9312)<br>ATM, S1691R (1.7%, 12686)
162	KIT, Y494H (1.3%), 620)<br>APC, P1415L (1.5%, 5552)<br>RET, E884V (1.2%, 5729)<br>SMAD4, Q256L (1.7%, 3025)<br>SMAD4, D493V (1.6%, 1706)	17	PTEN V133I (1.2%, 14422)

G

Figure 7. CTC analysis in clinical trial settings. A. Kaplan-Meier (KM) survival curves for number of CTCs captured by EpCAM immunoaffinity in SWOG-0421 from men with metastatic CRPC B. KM curves for CTC telomerase activity (TA) assayed from filter-captured cells in S0421. C. Ephrin B4 immunofluorescent staining of filter-captured cells in a Phase I trial. D. Merged (CK/DAPI/AFP) image of filter-captured CTCs from HCC patient. E. HCC-relevant gene mRNA expression from CTCs in the same trial. F. Cynvenio LiquidBiopsy Platform. G. CTC specimens from 4 patients with HSPC analyzed for mutations using the Cynvenio LiquidBiopsy platform

CTC analysis in **S0421** employed both microfiltration and immunoaffinity (cell surface marker) enrichment technologies in a parallel and complementary manner. Using these techniques, we generated the largest prospective overall survival data to date for immunoaffinity (CellSearch) CTC enumeration in first-line docetaxel-based therapy for mCRPC, and also demonstrated the first CTC-based molecular marker (telomerase activity from microfilter-captured cells) to be prognostic of overall survival in a large prospective trial setting (Figure 7A-B). These findings were reported in several podium presentations and submitted for publication. *(80,81)* We are also conducting CTC enumeration, protein staining and gene expression in several other clinical trial settings (Figure 7C-E): For example, in a V-Foundation funded first-in-human Phase I trial of a novel Ephrin-targeted drug developed at USC (NCT01642342), and in a Whittier Foundation funded trial of CTC biomarker characterization in localized and advanced hepatocellular carcinoma. In another study, we have been using the LiquidBiopsy CTC platform (Cynvenio, Inc), a microfluidic cell surface marker (EpCAM) immunoaffinity based technology that has been specifically developed for a NextGen sequencing workflow (Figure 7F). Using this approach, we captured

SWOG Authorized Document

and analyzed CTCs from a spectrum of malignancies including hormone sensitive PC (HSPC). AmpliSeq targeted sequencing on the Ion Torrent platform (Life Technologies) was used to identify somatic single nucleotide variants (SNV) present in CTCs but not in WBC. CTCs were detected in all 8 HSPC patients (CTC median 64.5, range 17-217), and significant SNVs (occurring in > 1% of DNA in a sample) were found in 4 of 8 patient CTC samples (range 1-5 SNVs/sample, frequency 1.2%-11.9% with 620X-14,422X coverage, Figure 7G). *(82)* Notably, in this pilot cohort, CTCs generated additional SNVs that were not found in matched primary tumor formalin-fixed, paraffin-embedded (FFPE) specimens, further reflecting the evolving biology of disease from early localized tumors to advanced metastatic disease. *(83)*

In summary, our extensive infrastructure and experience with multi-platform CTC specimen handling and molecular analysis in large clinical trial settings will play a key role in the current proposed project. This analysis will allow us to track how androgen pathway gene somatic variants and mRNA expression levels change in mPC over time, from initiation of ADT to emergence of castration resistance. Importantly, these parallel CTC genomic (2A) and expression (2B) studies will help to identify those androgen pathway genes in which particular somatic variants or alterations in expression level are predictive of response to therapy. Furthermore, the additional exploratory full transcriptome sequencing of 10 responders and 10 non-responders (2C) will yield additional candidate biomarkers and drivers of therapy response/resistance beyond androgen pathway genes alone.

Design
Please refer to the specimen collection instructions provided in Section 18.4.

Blood specimens for CTC capture and genomic analysis will be obtained with patient informed consent and central and institutional IRB approval at enrollment (HSPC) and upon progression (CRPC). At each of these 2 time points, 8-10 ml of peripheral blood will be drawn by standard venipuncture into each of 4 tubes (~35 ml total): One K3 vacutube (BD Biosciences, “Tube #1” in Figure 8) stabilized with the LiquidBiopsy stabilization kit, and 3 standard EDTA tubes (#2-4 in Figure 8). Samples will be shipped overnight at room temperature to the Goldkorn Lab at USC and processed within 24 hours of collection as follows:

SWOG Authorized Document

Aim 2A: A 7.5 mL aliquot from the stabilized blood sample will be washed by centrifugation and labeled with EpCAM ferrofluid. After incubation, the sample will be washed again and placed in the CLIA-certified LiquidBiopsy platform where it will be loaded through a CTC flow cell for magnetic capture of labeled cells (Figure 7F). After washing, captured cells will be treated in the CTC flow cell with a permeabilization buffer and incubated in the presence of the nuclear dye 4',6-diamidino-2-phenylindole (DAPI), CD45 antibodies conjugated to allophycocyanin (CD45-APC, InVitrogen) and pan-cytokeratin (CK) antibodies conjugated to fluorescein isothiocyanate (FITC, Sigma Aldrich). The CTC flow cell will then be transferred and scanned with a semi-automated fluorescent microscope scanner (Zeiss) for DAPI+CK+CD45-cells. Captured cells in the CTC flow cell then will be recovered by centrifugation. DNA will be extracted and used for library construction per manufacturer's instruction using a Life Technologies custom designed kit targeting androgen pathway genes (as done routinely by our group using the commercial AmpliSeq 2.0 kit and AmpliSeq Cancer Hot Spot Panel kits, Figure 7G). Ion Torrent sequencing, bioinformatics and treatement of genotypes will be performed in a CLIA certified laboratory as described for Aim 1 (Figure 5).

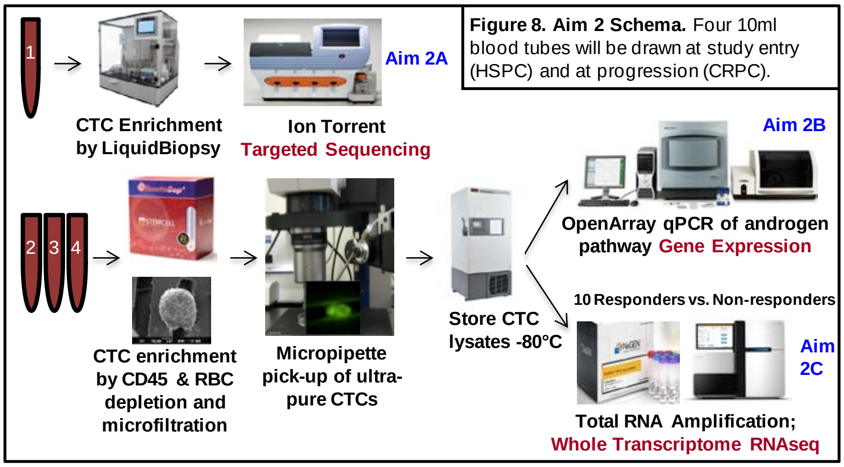

Figure 8. Aim 2 Schema. Four 10ml blood tubes will be drawn at study entry (HSPC) and at progression (CRPC).

Aim 2B-C: Blood from each of the three EDTA tubes will undergo WBC and red blood cell depletion using the Rossette Sep Kit (StemCell Tech, Inc) per manufacturer's instructions as done routinely in our laboratory. WBCs will be further depleted by passing each sample through a Parylene-C slot microfilter using a constant low-pressure delivery apparatus (Figure 6A-B). Cells will be stained using fluorescence-conjugated EpCAM and CD45 antibodies as well as Hoechst nuclear dye; cells will be visualized using a Zeiss AxioZoom V16 xyz motorized multispectral fluorescence microscope coupled to a high-performance AxioCam imaging system, and Hoechst+EpCAM+CD45- cells will be individually captured using an attached xyz motorized micropipette (Eppendorf) engineered for this purpose, as done previously (Figure 6C). Ultra-pure captured CTCs will be lysed for RNA isolation using the Pico-pure RNA isolation kit (Arcturus) and will be stored in XB lysis buffer in aliquots at -80ºC until subsequent RT/amplification steps.

OpenArray mRNA Analysis (Aim 2B): Frozen aliquots of CTC lysates will be thawed for measurement of mRNA transcript levels of androgen pathway genes (analyses will be batched for efficiency). The Open Array RT-PCR platform will be used, which enables highly multiplexed qPCR measurement of hundreds of

SWOG Authorized Document

genes simultaneously. For the purpose of this study, Life/ABI will provide a custom panel of pre-amplification and qPCR primers targeting the androgen pathway genes specified in Table 1 (androgen synthesis, metabolism, signaling and AR and its common splice variants such as AR-V7 and AR-V567es). Gene transcript quantification will be accomplished in two steps: First, mRNA will be mixed with primers targeting the genes of interest (Life/ABI) and combined with a PreAmp Master Mix (Life/ABI) per standard manufacturer's instructions in order to pre-amplify the target androgen pathway genes. Next, the product of the preamplification reaction will be loaded onto an Open-Array Real Time PCR System (Life/ABI) containing individual gene primer pairs in each well (pre-plated in triplicate along with internal control wells). The Open-Array RT-PCR System is available at the USC/Norris Genomics Core with ongoing technical support from Core personnel and Life/ABI (see two letters of support from Life Technologies), and it has been used successfully by our group to analyze CTCs (Figure 6F-G). For each androgen pathway gene, the mRNA expression level will be expressed as a mean of triplicate qPCR threshold cycles (Ct) normalized to housekeeping genes (GAPDH or β-actin).

RNAseq of CTCs from 10 responders vs. 10 non-responders (Aim 2C): During the course of follow-up in **S1216**, 10 patients designated as "non-responders" will be identified; similarly, 10 patients designated as "responders" will be identified. Non-responders will be selected from patients whose PSA nadir was > 4 ng/mL and who clinically progressed within 12 months, based on outcomes described in **SWOG-9346** by Hussain et al. *[84]* Responders will be selected from patients whose PSA nadir was < 1 ng/mL and did not progress within 36 months. For these 20 samples, frozen aliquots of CTC lysates will be thawed and amplified using the Nugen Ovation random hexamer linear amplification kit as done previously by our group (Figure 6D). Amplification products will be verified on an Agilent BioAnalyzer prior to standard library generation and sequencing at the USC/CHLA Genomics Core (Figure 5) or USC/Norris Genomics Core (HiSeq2000) as done previously (Figure 6E). RNA sequence reads will be analyzed using the Tuxedo suite of analysis tools. Reconstructed transcripts will be compared to the RefSeq genome annotation using IGV (Interactive Genome Viewer, Broad Institute), a visualization tool for interactive exploration of a wide variety of genomic data types, or the Genetrix browser, part of our academic software suite of tools developed at USC that also allows statistical analysis of the displayed data. RNA transcripts of any type (coding, non-coding, isoforms, RNA chimers, etc.) that show statistically significant differences in expression or usage between sample types will be included in an integrated pathway analysis (Ingenuity Pathway Analysis and similar) [please also see Statistical Methods section for additional analysis details].

SWOG
Authorized
Document

Expected *Results, Potential Pitfalls, and Alternative Approaches*

In Aim 2 we expect to find that genomic variations (Aim 2A) or mRNA expression levels (Aim 2B) of one or more androgen pathway genes are significantly associated with clinical outcome (PSA after 7 months and time to castration resistance). Also, we expect to glean important insights about androgen pathway genes that may drive progression from HSPC to CRPC, the two time points at which CTCs will be analyzed. Furthermore, we will have an opportunity to discover additional novel genes – beyond those included in the androgen pathway – which play a role in clinical outcome (Aim 2C). One potential challenge in Aim 2 could involve CTC collection from sufficient numbers of patients. Based on our own experience as well as prior studies by others, we expect to find CTCs in a large majority of patients (70% or more) prior to initiation of therapy and at castration resistance. *(85,86,87,88,89,90,91,92,93,94,95,96)* For example, in our pilot study using Cynvenio LiquidBiopsy for genomic analysis (presented ASCO 2013) we detected CTCs in all 8 men with HSPC and in **S0421** using CellSearch we detected CTCs in 73% of men with CRPC. *(97,98,99)* While detection rates can vary between platforms and cohorts, CTCs were detected in a large majority of men across these and other cited studies. *(100,101,102,103,104,105,106,107,108,109,110,111)* Furthermore, we have demonstrated our ability to identify somatic sequence variants in CTCs using a CLIA-certified targeted sequencing workflow (Liquid Biopsy, Figure 7F-G), as well as our ability to use the Open Array platform to successfully amplify and analyze CTC mRNA expression from a large gene panel (Figure 6F-G). For whole transcriptome profiling (Aim 2C) we have successfully performed total RNA amplification and RNAseq from CTCs (Figure 6D-E) using the Nugen Ovation kits, though other published kits (e.g. Clontech Smarter) also are available as an alternative. The quality of amplification products will be monitored using Agilent BioAnalyzer chips, and any potential candidate identified by our downstream bioinformatics/statistical analysis will be further validated in stored frozen samples using standard qPCR techniques.

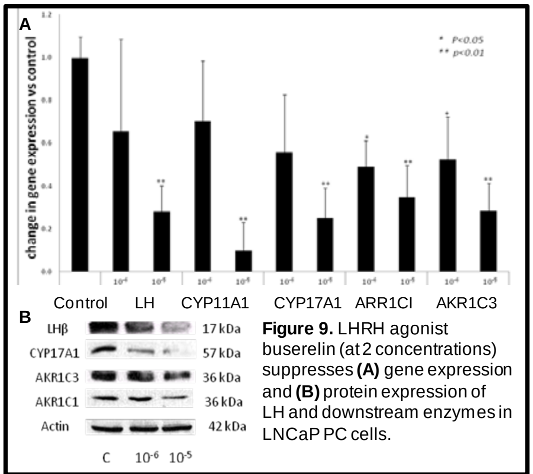

Figure 9. LHRH agonist buserelin (at 2 concentrations) suppresses **(A)** gene expression and **(B)** protein expression of LH and downstream enzymes in LNCaP PC cells.

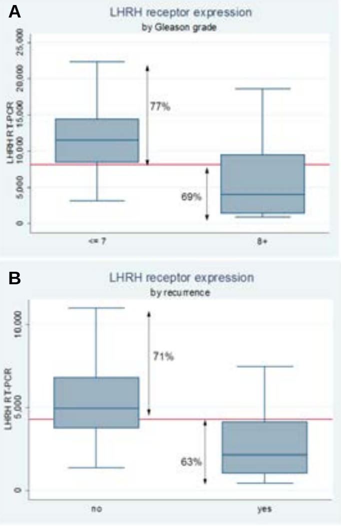

Figure 10. Relative LHRH-R mRNA expression by Gleason grade **(A)** and disease recurrence **(B)**.

Aim 3: Analyze androgen pathway genes in primary tumor cells for somatic sequence variants and RNA expression levels associated with response to therapy.

Rationale

Analyzing primary tumors for predictive biomarkers in mPC yields a two-fold benefit: 1. For treatment purposes, identifying predictive genes in primary tumor ultimately may enable use of abundant, readily available prostatectomy tissue to guide subsequent hormone therapy at the time of progression to metastatic disease. 2. From a biological perspective, comparing androgen pathway gene profiles in primary tumor (early localized disease) to those of CTCs (mPC) from the same patients would offer new insights into the changing biology of the androgen pathway in PC as it progresses from early localized to advanced metastatic disease.

Androgen pathway genes garnered attention because of the recent realization that androgen-related gene networks and AR remain active in CRPC cells. (91-92) Androgens responsible for this signaling can either be converted from adrenal precursors or synthesized de novo intratumorally, driving the tumor to castration resistance. *(112)* Higher levels of testosterone, DHT and steroidogenic enzymes were observed in primary PC tissue compared with benign prostate tissue, and in metastatic CRPC tissue compared with primary PC tissue. One important enzyme in this pathway, CYP17, is the target of orteronel and abiraterone, yet other enzymes and receptors in this pathway remain unexplored. A recent publication compared gene expression profiles and DNA copy number alteration (CNA) data from 29 normal prostate tissue samples, 127 primary PC, and 19 mPC. *(113)* The authors found that mPC expressed higher transcript levels for AR and several steroidogenic enzymes, including SRD5A1, SRD5A3, and AKR1C3, whereas expression of SRD5A2, CYP3A4, CYP3A5, and CYP3A7 was decreased. These findings support the growing consensus that gonadal suppression with an LHRH agonist does not eliminate androgens entirely, and that PC cells continue to encounter an androgen milieu to which they adapt by exploiting advantageous variations in their androgen pathway genes. These initial reports provide strong rationale for a more comprehensive assessment of androgen pathway as outlined in this proposal.

In addition to androgen deprivation through the hypothalamic-pituitary-gonadal axis, LHRH agonists have a direct effect on PC cells. *(114,115)* Our preliminary data show that treatment of PC cells with LHRH agonists decreases the expression of genes within the androgen pathway: Gene expression levels of LH and several enzymes related to androgen synthesis, including CYP11A1, CYP17A1, AKR1C1 and AKR1C3 were significantly down-regulated in LNCaP cells exposed to an LHRH agonist (Figure 9A), with a corresponding decrease in protein expression (Figure 9B). Our studies also suggest that expression differences in androgen pathway genes may identify subgroups of patients less likely to achieve a long-lasting response to ADT. LHRH receptor (LHRH-R) is expressed on PC cells and we have extensively studied and reported the LHRH-R phenotype in PC. *(116,117,118,119)* Clinically, our data demonstrate an association between LHRH-R expression and both Gleason grade and PC recurrence (Figure 10): Paraffin embedded specimens from 40 men treated with prostatectomy for PC were microdissected for areas of cancer and RNA was extracted. LHRH-R expression was analyzed using real-time PCR and was significantly higher in men with Gleason grade ≤ 7 as compared to men with

SWOG Authorized Document

Gleason > 7 (p<0.02, Figure 10A). Higher LHRH-R expression was also associated with lower disease recurrence (Figure 10B). Thus, we have shown that LHRH agonists decrease expression of androgen pathway genes in PC cells, and that low LHRH-R expression portends poor prognosis. Based on these findings, we reason that a reduced response to LHRH agonist therapy may be mediated by low LHRH-R expression, which prevents LHRH agonists from effectively down-regulating androgen synthesis genes, supporting our investigation of these genes as predictive markers.

In other work, we showed that PC cells express LHR and produce LH in an autocrine manner. *(120)* In both androgen-sensitive (LNCaP) and androgen-independent PC cells (C4-2B and 22RV1), we demonstrated that exposure to LH produced a significant dose-dependent increase in expression of androgen pathway genes at both the mRNA and protein level, as well as increased androgen synthesis. Conversely, siRNA knock-down of LHR produced a significant decrease in the expression of the same genes and proteins. (99) Based on these data, we expect that increased expression of LH and LHR could drive PC cells toward a castrate resistant state by increasing de novo androgen synthesis and may predict poor response to ADT.

Design

In Aim 3, we will analyze DNA and RNA from microdissected prostatectomy specimens by targeted sequencing for somatic variants and by Open Array PCR for gene transcript levels and androgen pathway (Figure 11). We will identify those androgen pathway genes in which particular somatic variants or alterations in expression level are predictive of disease response to therapy (PSA value after 7 months of therapy and time to castration resistance). In addition, these results will be compared with somatic variants and transcript levels measured in CTCs from the same subjects. As an exploratory sub-aim, whole transcriptomes from microdissected prostatectomy specimens of 10 responders and 10 non-responders will be amplified and sequenced to discover novel differentially-expressed gene candidates that predict and possibly drive response and resistance to hormonal therapy. The analysis will be conducted using FFPE prostatectomy specimens

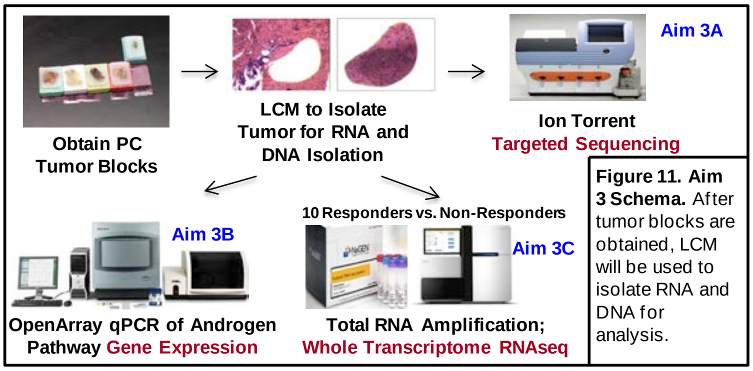

Figure 11. Aim 3 Schema. After tumor blocks are obtained, LCM will be used to isolate RNA and DNA for analysis.

Tissue procurement: For patients with a history of radical prostatectomy, tissue blocks will be obtained from the institution where their surgery was performed. Tumor DNA and RNA will be extracted from FFPE blocks of prostatectomy specimens as follows: Ten 10 µM sections will be cut and mounted on glass slides without cover slips. H&E staining will be used to identify areas of tumors cells in the section. The slide to be microdissected (a "sister cut") and stained with nuclear fast red, and malignant cells will be excised and collected for extraction of DNA and RNA. Dr. Vallone, a board-certified pathologist at USC, will be responsible for examining the stained slides and identifying areas of tumor

SWOG Authorized Document

cells to be excised. Using the PixCell II LCM System (Arcturus), we will collect an average of 2000-4000 cells per malignant epithelial gland using CapSure LCM caps.

DNA sequence variants (Aim 3A): DNA will be purified from the tissue lysate by buffer-saturated phenol (Thermo/Fisher) extraction. RNase A (100ug/ml, 1 hour at 37°C) will be then added to eliminate contaminating RNA. Additional phenol extractions following incubation with RNase A will be used to remove any remaining enzyme. DNA will be precipitated by sodium acetate and isopropanol, followed by washing with 70% ethanol. Purified DNA will be stored at -80°C and thawed in batches for for library construction and targeted sequencing using the AmpiSeq and Ion Torrent platforms in a CLIA certified laboratory as described in Aim 1 (Figure 5).

RNA expression analysis (Aim 3B): RNA will be purified from the microdissected tissue using Qiagen AllPrep RNA FFPE Kit per manufacturer's instructions (we have extensive experience with these methods). (100-101) Purified RNA will be stored at 50 ng/□L in RNase-free water at -80°C and thawed in batches for RT-PCR analysis of androgen pathway gene transcript levels, and at the time of thawing RNA integrity will be assessed by RT-PCR for RPL13A using the QuantiTect Primer Assay QT00089915 (Qiagen). Pre-amplification and quantitation of androgen pathway genes (Table 1) and an internal reference gene (GAPDH or β-actin) will be done in triplicate using the Open Array Real Time PCR System (Life/ABI) as described in Aim 2 (Figure 6F-G).

Whole transcriptome analysis (Aim 3C): In an exploratory experiment on 10 responders and 10 non-responders (as defined in Aim 2), we will amplify and sequence whole transcriptomes to discover novel differentially-expressed gene candidates that predict and possibly drive response and resistance to hormonal therapy (Figure 5). To accomplish this, stored RNA samples extracted from microdissected specimens (see above) will be thawed in batches and used for RNA sequencing as described in Aim 2. Whereas preamplification steps are already built into targeted sequencing and OpenArray analysis (Aim 3A and 3B, respectively), we may have enough total RNA extracted for Aim 3C to proceed with RNAseq without the prior whole transcriptome preamplification that is necessary in Aim 2C, which uses CTCs.

Expected Results, Potential Pitfalls, and Alternative Approaches
We expect to find androgen pathway genes whose somatic expression levels or sequence variation will be associated with clinical response to ADT (PSA value after 7 months of therapy and time to castration resistance). One challenge inherent to extraction of RNA from FFPE tissues is RNA degradation. Based on our previous experience with FFPE samples, we anticipate degradation in 5-10% of samples for which we adjusted our sample size. Moreover, RNA integrity will be assessed by RT-PCR for RPL13A using the QuantiTect Primer Assay QT00089915 (Qiagen) as described.

Another challenge encountered when using FFPE samples is the possibility of artifactual mutations that may be produced due to formalin fixation and long-term storage. (121) Possible mechanisms include cytosine deaminations, guanine oxidations, and, most importantly, recombination between degraded DNA fragments ("PCR template jumping"), which can be suppressed by reducing the PCR amplicon size. (104) In sequence data, the presence of artifactual mutations can be recognized by a higher *global* nucleotide mismatch rate among aligned

SWOG Authorized Document

reads in tumor compared to germline sequence. Using global and local mismatch rates, Yost et al have designed easily-implemented post-alignment filters to remove false positive calls, thus allowing identification of somatic mutations in the face of FFPE induced DNA damage. *([122])*

References

1 Fontana A, Delmas PD. Markers of bone turnover in bone metastases. Cancer 88(12 Suppl):2952-2960, 2000.

2 Lara PN, Jr., Stadler WM, Longmate J, et al. A randomized phase II trial of the matrix metalloproteinase inhibitor BMS-275291 in hormone-refractory prostate cancer patients with bone metastases. Clin Cancer Res 12(5):1556-1563, 2006.

3 Kamiya N, Suzuki H, Yano M, et al. Implications of serum bone turnover markers in prostate cancer patients with bone metastasis. Urology 75(6):1446-1451, 2010.

4 Thurairaja R, Iles RK, Jefferson K, McFarlane JP, Persad RA. Serum amino-terminal propeptide of type 1 procollagen (P1NP) in prostate cancer: a potential predictor of bone metastases and prognosticator for disease progression and survival. Urol Int 76(1):67-71, 2006.

5 Smith JA, Jr., Lange PH, Janknegt RA, Abbou CC, deGery A. Serum markers as a predictor of response duration and patient survival after hormonal therapy for metastatic carcinoma of the prostate. J Urol 157(4):1329-1334, 1997.

6 Somner J, McLellan S, Cheung J, et al. Polymorphisms in the P450 c17 (17-hydroxylase/17,20-Lyase) and P450 c19 (aromatase) genes: association with serum sex steroid concentrations and bone mineral density in postmenopausal women. J Clin Endocrinol Metab 89(1): 344-351, 2004.

7 White PC, New MI, Dupont B. Congenital adrenal hyperplasia. N Engl J Med 316(24):1519-1524, 1987.

8 Guo CY, Weetman AP, Eastell R. Bone turnover and bone mineral density in patients with congenital adrenal hyperplasia. Clin Endocrinol (Oxf) 45(5):535-541, 1996.

9 Crawford ED, Eisenberger MA, McLeod DG, et al. A controlled trial of leuprolide with and without flutamide in prostatic carcinoma. N Engl J Med 321: 419-24, 1989.

10 Scher HI, Fizazi K, Saad F, et al. Increased survival with enzalutamide in prostate cancer after chemotherapy. N Engl J Med 367: 1187-97, 2012.

11 Ryan CJ, Smith MR, de Bono JS, et al. Abiraterone in metastatic prostate cancer without previous chemotherapy. N Engl J Med 368: 138-48, 2013.

12 Chang K-H, Li R, Kuri B, et al. A Gain-of-Function Mutation in DHT Synthesis in Castration-Resistant Prostate Cancer. Cell 154: 1074-1084, 2013.

13 Kohli M, Riska SM, Mahoney DW, et al. Germline predictors of androgen deprivation therapy response in advanced prostate cancer. Mayo Clin Proc 87: 240-46, 2012.

14 Mostaghel EA, Marck BT, Plymate SR, et al. Resistance to CYP17A1 inhibition with abiraterone in castration-resistant prostate cancer: induction of steroidogenesis and androgen receptor splice variants. Clin Cancer Res 17: 5913-25, 2011.

15 Li Y, Chan SC, Brand LJ, et al. Androgen receptor splice variants mediate enzalutamide resistance in castration-resistant prostate cancer cell lines. Cancer Res 73: 483-89, 2013.

16 Liu SV, Pinski J, Ingles S. Prostate cancer outcomes and polymorphism in LHRH and LH receptors and SHBG. ASCO Genitourinary Cancers Symposium 2011, abstract 5.

SWOG Authorized Document

17 Liu SV, Dorff TB, Wang Q, et al. LH receptor polymorphisms and response to androgen deprivation therapy in prostate cancer. ASCO Annual Meeting 2011, abstract 4597.

18 Siegel R, Naishadham D, Jemal A. Cancer statistics, 2012. CA Cancer J Clin 62:10-29, 2012.

19 Crawford ED, Eisenberger MA, McLeod DG, et al. A controlled trial of leuprolide with and without flutamide in prostatic carcinoma. N Engl J Med 321: 419-24, 1989.

20 Ross RW, Xie W, Regan MM, et al. Efficacy of androgen deprivation therapy (ADT) in advanced prostate cancer: association with Gleason score, prostate-specific antigen level, and prior ADT exposure with duration of ADT effect. Cancer 112: 1247-53, 2008.

21 Stanbrough M, Bubley GJ, Ross K, et al. Increased expression of genes converting adrenal androgens to testosterone in androgen-independent prostate cancer. Cancer Res 66: 2815-25, 2006.

22 Montgomery RB, Mostaghel EA, Vessella R, et al. Maintenance of intratumoral androgens in metastatic prostate cancer: a mechanism for castration-resistant tumor growth. Cancer Res 68: 4447-54, 2008.

23 Chang K-H, Li R, Kuri B, et al. A Gain-of-Function Mutation in DHT Synthesis in Castration-Resistant Prostate Cancer. Cell 154: 1074-1084, 2013.

24 Kohli M, Riska SM, Mahoney DW, et al. Germline predictors of androgen deprivation therapy response in advanced prostate cancer. Mayo Clin Proc 87: 240-46, 2012.

25 Mostaghel EA, Marck BT, Plymate SR, et al. Resistance to CYP17A1 inhibition with abiraterone in castration-resistant prostate cancer: induction of steroidogenesis and androgen receptor splice variants. Clin Cancer Res 17: 5913-25, 2011.

26 Li Y, Chan SC, Brand LJ, et al. Androgen receptor splice variants mediate enzalutamide resistance in castration-resistant prostate cancer cell lines. Cancer Res 73: 483-89, 2013.

27 Liu SV, Pinski J, Ingles S. Prostate cancer outcomes and polymorphism in LHRH and LH receptors and SHBG. ASCO Genitourinary Cancers Symposium 2011, abstract 5.

28 Liu SV, Dorff TB, Wang Q, et al. LH receptor polymorphisms and response to androgen deprivation therapy in prostate cancer. ASCO Annual Meeting 2011, abstract 4597.

29 Agus DA, Stadler WM, Shevrin DH, et al. Safety, efficacy, and pharmacodynamics of the investigational agent orteronel (TAK-700) in metastatic castration-resistant prostate cancer (mCRPC): Updated data from a phase I/II study. J Clin Oncol 30: (suppl 5; abstr 98), 2012.

30 Hussain M, Corn P, Michaelson D, et al. Activity and safety of the investigational agent orteronel in men with nonmetastatic castration-resistant prostate cancer and rising prostate-specific antigen: results of a phase 2 study. 27th Annual European Association of Urology, Paris, France, 24–28: poster 124, 2012.

31 Ross RW, Xie W, Regan MM, et al. Efficacy of androgen deprivation therapy (ADT) in advanced prostate cancer: association with Gleason score, prostate-specific antigen level, and prior ADT exposure with duration of ADT effect. Cancer 112: 1247-53, 2008.

32 Chang K-H, Li R, Kuri B, et al. A Gain-of-Function Mutation in DHT Synthesis in Castration-Resistant Prostate Cancer. Cell 154: 1074-1084, 2013.

SWOG Authorized Document

33 Kohli M, Riska SM, Mahoney DW, et al. Germline predictors of androgen deprivation therapy response in advanced prostate cancer. Mayo Clin Proc 87: 240-46, 2012.

34 Mostaghel EA, Marck BT, Plymate SR, et al. Resistance to CYP17A1 inhibition with abiraterone in castration-resistant prostate cancer: induction of steroidogenesis and androgen receptor splice variants. Clin Cancer Res 17: 5913-25, 2011.

35 Li Y, Chan SC, Brand LJ, et al. Androgen receptor splice variants mediate enzalutamide resistance in castration-resistant prostate cancer cell lines. Cancer Res 73: 483-89, 2013.

36 Liu SV, Pinski J, Ingles S. Prostate cancer outcomes and polymorphism in LHRH and LH receptors and SHBG. ASCO Genitourinary Cancers Symposium 2011, abstract 5.

37 Liu SV, Dorff TB, Wang Q, et al. LH receptor polymorphisms and response to androgen deprivation therapy in prostate cancer. ASCO Annual Meeting 2011, abstract 4597.

38 Mostaghel EA, Marck BT, Plymate SR, et al. Resistance to CYP17A1 inhibition with abiraterone in castration-resistant prostate cancer: induction of steroidogenesis and androgen receptor splice variants. Clin Cancer Res 17: 5913-25, 2011.

39 Li Y, Chan SC, Brand LJ, et al. Androgen receptor splice variants mediate enzalutamide resistance in castration-resistant prostate cancer cell lines. Cancer Res 73: 483-89, 2013.

40 Mitsiades N, Sung CC, Schultz N, et al. Distinct patterns of dysregulated expression of enzymes involved in androgen synthesis and metabolism in metastatic prostate cancer tumors. Cancer Res 72: 6142-52, 2012.

41 Cai C, Chen S, Ng P, et al. Intratumoral de novo steroid synthesis activates androgen receptor in castration-resistant prostate cancer and is upregulated by treatment with CYP17A1 inhibitors. Cancer Res 71: 6503-13, 2011.

42 Liu SV, Pinski J, Ingles S. Prostate cancer outcomes and polymorphism in LHRH and LH receptors and SHBG. ASCO Genitourinary Cancers Symposium 2011, abstract 5.

43 Pinski J, Xiong S, Wang Q, et al. Effect of luteinizing hormone on the steroidogenic pathway in prostate cancer. Prostate 71: 892-98, 2011.

44 Hussain M, Tangen CM, Higano C, et al. Absolute prostate-specific antigen value after androgen deprivation is a strong independent predictor of survival in new metastatic prostate cancer: data from Southwest Oncology Group Trial 9346 (INT-0162). J Clin Oncol 24: 3984-90, 2006.

45 Hussain M, Goldman B, Tangen C, et al. Prostate-specific antigen progression predicts overall survival in patients with metastatic prostate cancer: data from Southwest Oncology Group trials 9346 (Intergroup study 0162) and 9916. J Clin Oncol 27: 2450-2456, 2009.

46 Pinski J, Xiong S, Wang Q, et al. Effect of luteinizing hormone on the steroidogenic pathway in prostate cancer. Prostate 71: 892-98, 2011.

50 Manolio TA. Genomewide association studies and assessment of the risk of disease. N Engl J Med 363: 166-76, 2010.

51 Shete S, Lau CC, Houlston RS, et al. Genome-wide high-density SNP linkage search for glioma susceptibility loci: results from the Gliogene Consortium. Cancer Res 71: 7568-75, 2011.

52 Audet-Walsh E, Bellemare J, Lacombe L, et al. The impact of germline genetic variations in hydroxysteroid (17-beta) dehydrogenases on prostate cancer outcomes after prostatectomy. Eur Urol 62: 88-96, 2012.

53 Liu SV, Pinski J, Ingles S. Prostate cancer outcomes and polymorphism in LHRH and LH receptors and SHBG. ASCO Genitourinary Cancers Symposium 2011, abstract 5.

54 Liu SV, Dorff TB, Wang Q, et al. LH receptor polymorphisms and response to androgen deprivation therapy in prostate cancer. ASCO Annual Meeting 2011, abstract 4597.

55 Schork NJ, Murray SS, Frazer KA, Topol EJ. Common vs. rare allele hypotheses for complex diseases. Current Opinions in Genetics & Development 19: 212-19, 2009.

56 Manolio TA, Collins FS, Cox NJ, Get al. Finding the missing heritability of complex diseases. Nature 461: 747–53, 2009.

57 Nord AS, Lee M, King M-C, Walsh T. Accurate and exact CNV identification from targeted high-throughput sequence data. BMC Genomics 12: 184, 2011.

58 Wu J, Grzeda KR, Stewart C, et al. Copy Number Variation detection from 1000 genomes project exon capture sequencing data. BMC Bioinformatics 13: 305, 2012.

59 Gao X, Starmer J, Martin E. A multiple testing correction method for genetic association studies using correlated single nucleotide polymorphisms. Genetic Epidemiology 32: 361-69, 2008.

60 Johansen CT, Wang J, Lanktree MB, et al. Excess of rare variants in genes identified by genome-wide association study of hypertriglyceridemia. Nat Genet 42: 684-98, 2010.

61 Schork NJ, Wessel J, Maio N. DNA Sequence-based phenotypic association analysis. Advances in Genetics 60: 195-217, 2008.

62 Ali A, Furusato B, Ts'o PO, et al. Assessment of circulating tumor cells (CTCs) in prostate cancer patients with low-volume tumors. Pathol Int 60: 667-72. PubMed, 2010.

63 Okegawa T, Nutahara K, Higashihara E. Prognostic significance of circulating tumor cells in patients with hormone refractory prostate cancer. J Urol. 181: 1091-97, 2009.

64 Alix-Panabieres C, Schwarzenbach H, Pantel K. Circulating tumor cells and circulating tumor DNA. Annu Rev Med 63: 199-215, 2012.

68 Ali A, Furusato B, Ts'o PO, et al. Assessment of circulating tumor cells (CTCs) in prostate cancer patients with low-volume tumors. Pathol Int 60: 667-72. PubMed, 2010.

69 Okegawa T, Nutahara K, Higashihara E. Prognostic significance of circulating tumor cells in patients with hormone refractory prostate cancer. J Urol. 181: 1091-97, 2009.

70 de Bono JS, Scher HI, Montgomery RB, et al. Circulating tumor cells predict survival benefit from treatment in metastatic castration-resistant prostate cancer. Clin Cancer Res 2008; 14: 6302-9. Erratum in: Clin Cancer Res 15: 1506, 2009.

71 Danila DC, Anand A, Sung CC, et al. TMPRSS2-ERG status in circulating tumor cells as a predictive biomarker of sensitivity in castration-resistant prostate cancer patients treated with abiraterone acetate. Eur Urol 60: 897-904, 2011.

72 Jiang Y, Palma JF, Agus DB, Wang Y, Gross ME. Detection of androgen receptor mutations in circulating tumor cells in castration-resistant prostate cancer. Clin Chem 56: 1492-95, 2010.

73 Shaffer DR, Leversha MA, Danila DC, et al. Circulating tumor cell analysis in patients with progressive castration-resistant prostate cancer. Clin Cancer Res 13: 2023-29, 2007.

74 Goodman OB Jr, Symanowski JT, Loudyi A, et al. Circulating tumor cells as a predictive biomarker in patients with hormone-sensitive prostate cancer. Clin Genitourin Cancer 9: 31-38, 2011.

75 Scher HI, Jia X, de Bono JS, et a.. Circulating tumour cells as prognostic markers in progressive, castration-resistant prostate cancer: a reanalysis of IMMC38 trial data. Lancet Oncol 10: 233–39, 2009.

76 Scher HI, Heller G, Molina A, et al. Evaluation of circulating tumor cell (CTC) enumeration as an efficacy response biomarker of overall survival (OS) in metastatic castration-resistant prostate cancer (mCRPC): Planned final analysis (FA) of COU-AA-301, a randomized double-blind, placebo-controlled phase III study of abiraterone acetate (AA) plus low-dose prednisone (P) post docetaxel. J Clin Oncol 29:(suppl; abstr LBA4517), 2011.

77 Danila DC, Heller G, Gignac GA, et al. Circulating tumor cell number and prognosis in progressive castration-resistant prostate cancer. Clin Cancer Res 13: 7053-58, 2007.

78 Moreno JG, Miller MC, Gross S, et a.. Circulating tumor cells predict survival in patients with metastatic prostate cancer. Urology 65: 713-18, 2005.

79 Xu T, Lu B, Tai YC, Goldkorn A. A cancer detection platform which measures telomerase activity from live circulating tumor cells captured on a microfilter. Cancer Res 70: 6420-26, 2010.

80 Goldkorn A, Xu T, Lu B, et al. Circulating tumor cell capture and analysis in a multicenter SWOG-coordinated prostate cancer trial. J Clin Oncol 28:15s, 2010 (suppl; abstr TPS342) ASCO Annual Meeting 2010.

83 Liu SV, Dempsey PW, Strauss W, Xu Y, et al. Targeted next generation sequencing (NGS) of circulating tumor cells (CTCs) in hormone-sensitive prostate cancer (HSPC). ASCO Annual Meeting 2013, abstract 11040.

84 Hussain M, Tangen CM, Higano C, et al. Absolute prostate-specific antigen value after androgen deprivation is a strong independent predictor of survival in new metastatic prostate cancer: data from Southwest Oncology Group Trial 9346 (INT-0162). J Clin Oncol 24: 3984-90, 2006.

85 de Bono JS, Scher HI, Montgomery RB, et al. Circulating tumor cells predict survival benefit from treatment in metastatic castration-resistant prostate cancer. Clin Cancer Res 2008; 14: 6302-9. Erratum in: Clin Cancer Res 15: 1506, 2009.

86 Danila DC, Anand A, Sung CC, et al. TMPRSS2-ERG status in circulating tumor cells as a predictive biomarker of sensitivity in castration-resistant prostate cancer patients treated with abiraterone acetate. Eur Urol 60: 897-904, 2011.

87 Jiang Y, Palma JF, Agus DB, Wang Y, Gross ME. Detection of androgen receptor mutations in circulating tumor cells in castration-resistant prostate cancer. Clin Chem 56: 1492-95, 2010.

88 Shaffer DR, Leversha MA, Danila DC, et al. Circulating tumor cell analysis in patients with progressive castration-resistant prostate cancer. Clin Cancer Res 13: 2023-29, 2007.

89 Goodman OB Jr, Symanowski JT, Loudyi A, et al. Circulating tumor cells as a predictive biomarker in patients with hormone-sensitive prostate cancer. Clin Genitourin Cancer 9: 31-38, 2011.

90 Scher HI, Jia X, de Bono JS, et a.. Circulating tumour cells as prognostic markers in progressive, castration-resistant prostate cancer: a reanalysis of IMMC38 trial data. Lancet Oncol 10: 233–39, 2009.

91 Scher HI, Heller G, Molina A, et al. Evaluation of circulating tumor cell (CTC) enumeration as an efficacy response biomarker of overall survival (OS) in metastatic castration-resistant prostate cancer (mCRPC): Planned final analysis (FA) of COU-AA-301, a randomized double-blind, placebo-controlled phase III study of abiraterone acetate (AA) plus low-dose prednisone (P) post docetaxel. J Clin Oncol 29:(suppl; abstr LBA4517), 2011.

92 Danila DC, Heller G, Gignac GA, et al. Circulating tumor cell number and prognosis in progressive castration-resistant prostate cancer. Clin Cancer Res 13: 7053-58, 2007.

93 Goldkorn A, Xu T, Lu B, et al. Circulating tumor cell capture and analysis in a multicenter SWOG-coordinated prostate cancer trial. J Clin Oncol 28:15s, 2010 (suppl; abstr TPS342) ASCO Annual Meeting 2010.

94 Vogelzang NJ, Ely B, Fink LM, et al. Circulating tumor cell counts (CTC) as prognostic of overall survival (OS) in SWOG S0421-docetaxel with or without atrasentan for metastatic castration resistant prostate cancer (mCRPC). J Clin Oncol 30 (suppl; abstr 10503), 2012.

97 Goldkorn A, Xu T, Lu B, et al. Circulating tumor cell capture and analysis in a multicenter SWOG-coordinated prostate cancer trial. J Clin Oncol 28:15s, 2010 (suppl; abstr TPS342) ASCO Annual Meeting 2010.

98 Vogelzang NJ, Ely B, Fink LM, et al. Circulating tumor cell counts (CTC) as prognostic of overall survival (OS) in SWOG S0421-docetaxel with or without atrasentan for metastatic castration resistant prostate cancer (mCRPC). J Clin Oncol 30 (suppl; abstr 10503), 2012.

99 Goldkorn A, Vogelzang NJ, Fink LM, et al. Circulating tumor cell (CTC) counts and CTC telomerase activity (TA) as prognostic markers of overall survival (OS) in SWOG S0421: docetaxel with or without atrasentan for metastatic castration-resistant prostate cancer (mCRPC). J Clin Oncol 30 (suppl 30; abstr 1), 2012.

100 de Bono JS, Scher HI, Montgomery RB, et al. Circulating tumor cells predict survival benefit from treatment in metastatic castration-resistant prostate cancer. Clin Cancer Res 2008; 14: 6302-9. Erratum in: Clin Cancer Res 15: 1506, 2009.

101 Danila DC, Anand A, Sung CC, et al. TMPRSS2-ERG status in circulating tumor cells as a predictive biomarker of sensitivity in castration-resistant prostate cancer patients treated with abiraterone acetate. Eur Urol 60: 897-904, 2011.

102 Jiang Y, Palma JF, Agus DB, Wang Y, Gross ME. Detection of androgen receptor mutations in circulating tumor cells in castration-resistant prostate cancer. Clin Chem 56: 1492-95, 2010.

103 Shaffer DR, Leversha MA, Danila DC, et al. Circulating tumor cell analysis in patients with progressive castration-resistant prostate cancer. Clin Cancer Res 13: 2023-29, 2007.

104 Goodman OB Jr, Symanowski JT, Loudyi A, et al. Circulating tumor cells as a predictive biomarker in patients with hormone-sensitive prostate cancer. Clin Genitourin Cancer 9: 31-38, 2011.

105 Scher HI, Jia X, de Bono JS, et a.. Circulating tumour cells as prognostic markers in progressive, castration-resistant prostate cancer: a reanalysis of IMMC38 trial data. Lancet Oncol 10: 233–39, 2009.

106 Scher HI, Heller G, Molina A, et al. Evaluation of circulating tumor cell (CTC) enumeration as an efficacy response biomarker of overall survival (OS) in metastatic castration-resistant prostate cancer (mCRPC): Planned final analysis (FA) of COU-AA-301, a randomized double-blind, placebo-controlled phase III study of abiraterone acetate (AA) plus low-dose prednisone (P) post docetaxel. J Clin Oncol 29:(suppl; abstr LBA4517), 2011.

107 Danila DC, Heller G, Gignac GA, et al. Circulating tumor cell number and prognosis in progressive castration-resistant prostate cancer. Clin Cancer Res 13: 7053-58, 2007.

108 Goldkorn A, Xu T, Lu B, et al. Circulating tumor cell capture and analysis in a multicenter SWOG-coordinated prostate cancer trial. J Clin Oncol 28:15s, 2010 (suppl; abstr TPS342) ASCO Annual Meeting 2010.

111 Liu SV, Dempsey PW, Strauss W, Xu Y, et al. Targeted next generation sequencing (NGS) of circulating tumor cells (CTCs) in hormone-sensitive prostate cancer (HSPC). ASCO Annual Meeting 2013, abstract 11040.

112 Montgomery RB, Mostaghel EA, Vessella R, et al. Maintenance of intratumoral androgens in metastatic prostate cancer: a mechanism for castration-resistant tumor growth. Cancer Res 68: 4447-54, 2008.

113 Hussain M, Corn P, Michaelson D, et al. Activity and safety of the investigational agent orteronel in men with nonmetastatic castration-resistant prostate cancer and rising prostate-specific antigen: results of a phase 2 study. 27th Annual European Association of Urology, Paris, France, 24–28: poster 124, 2012.

114 Locke JA, Guns ES, Lubik AA, et al. Androgen levels increase by intratumoral de novo steroidogenesis during progression of castration-resistant prostate cancer. Cancer Res 68: 6407-15, 2008.

115 Limonta P, Moretti RM, Marelli MM, et al. The luteinizing hormone-releasing hormone receptor in human prostate cancer cells: messenger ribonucleic acid expression, molecular size, and signal transduction pathway. Endocrinology 140: 5250-56, 1999.

116 Kraus S, Naor Z, Seger R. Gonadotropin-releasing hormone in apoptosis of prostate cancer cells. Cancer Lett 234: 109-23, 2006.

117 Dirnhofer S, Berger C, Hermann M, et al. Coexpression of gonadotropic hormones and their corresponding FSH- and LH/CG-receptors in the human prostate. Prostate 35: 212-20, 1998.

118 Tao YX, Bao S, Ackermann DM, et al. Expression of luteinizing hormone/human chorionic gonadotropin receptor gene in benign prostatic hyperplasia and in prostate carcinoma in humans. Biol Reprod 56: 67-72, 1997.

119 Halmos G, Arencibia JM, Schally AV, Davis R, Bostwick DG. High incidence of receptors for luteinizing hormone-releasing hormone (LHRH) and LHRH receptor gene expression in human prostate cancers. J of Urol 163: 623-29, 2000.

120 Liu SV, Pinski J, Ingles S. Prostate cancer outcomes and polymorphism in LHRH and LH receptors and SHBG. ASCO Genitourinary Cancers Symposium 2011, abstract 5.

121 Mori R, Xiong S, Wang Q, Tet al. Gene profiling and pathway analysis of neuroendocrine transdifferentiated prostate cancer cells. Prostate 69: 12-23, 2009.

122 Lamy A, Blanchard F, Le Pessot F, et al. Metastatic colorectal cancer KRAS genotyping in routine practice: results and pitfalls. Mod Pathol 24: 1090-100, 2011.

SWOG Authorized Document

18.3 Instructions for blood sample processing, packaging, and shipping for circulating tumor cell (CTC) analysis in **S1216**

For questions or to order kits, please contact (in order of preference):
Yucheng Xu, Ph.D. (Goldkorn Lab): yuchengx@usc.edu
Tong Xu, Ph.D. (Goldkorn Lab): tongxu@usc.edu
Ahva Shahabi, Ph.D.: ahvashah@usc.edu
Amir Goldkorn, M.D.: agoldkor@usc.edu
Tel: 323 442-7722

Please collect blood into 1 CellSave and 3 EDTA tubes (provided in kit) and label them with subject ID and date. After collection, one of the 3 EDTA tubes will be immediately processed as outlined below in the LiquidBiopsy Sample Processing Protocol. The processed specimen will be packaged and shipped along with the remaining CellSave and 2 EDTA tubes (see schema below).

Sample collection schema

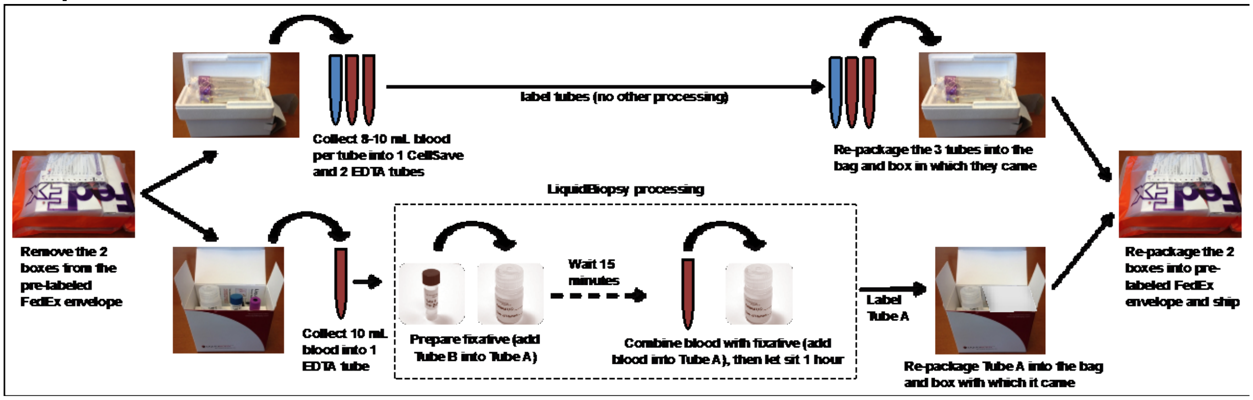

Blood Collection

1. Remove the 1 CellSave and 3 EDTA tubes (4 tubes total) provided in the pre-packaged kits.

2. Collect 8-10 mL blood per tube and label with subject ID and date.

3. The CellSave and 2 of the EDTA tubes require no additional processing and are ready for shipping (see Packaging and Shipping, next page).

4. Use the third EDTA tube for LiquidBiopsy processing as outlined below.

LiquidBiopsy^{TM} Processing

<u>Prepare Fixative</u>

1. Prepare a working fixative solution by adding the entire contents of Tube B into Tube A.

2. Mix the two solutions GENTLY by inversion.

3. Allow the solution to stand at room temperature for **15 minutes to activate**.

SWOG Authorized Document

Combine Blood with Fixative

4. Once the fixative is activated, empty the entire contents of the EDTA blood tube into the Tube A working fixative solution.

5. Secure Tube A lid tightly. Do not add sealants or parafilm to threads.

Label the Sample

6. Write the Subject ID, Date of Harvest, and Lab/Clinic Info on the Tube A label using a permanent marker.

7. Invert Tube A GENTLY by hand 3 times to mix.

8. Allow the specimen to sit undisturbed for 1 hour.

LiquidBiopsy Kit Warnings

- This kit contains chemicals OSHA designates as hazardous or irritants. For specific details and handling instructions, see the Material Safety Data Sheets available at www.cynvenio.com/sample-handling.
- Use good laboratory practices when handling, including proper personal protective equipment and universal precautions.
- In case of contact, rinse immediately with excess water.

Packaging & Shipping Instructions for All Tubes

1. All tubes are stored and shipped at room temperature.

2. Re-package the 1 CellSave tube and 2 EDTA tubes (3 total) into the biohazard bag and Styrofoam shipping container in which these tubes originally came.

3. Re-package Tube A into the biohazard bag and LiquidBiopsy™ box in which it originally came.

4. Place the closed Styrofoam container (3 tubes) and closed LiquidBiopsy™ box (1 tube) into the pre-labeled FedEx Clinical Pak in which they originally came, and seal the pack.

5. Call FedEx at 800-463-3339 for courier pick-up no later than 3PM Monday - Friday.

Note: Do not place the shipment in a drop box.

SWOG Authorized Document

18.4 New York Heart Association Classifications

Class	Cardiac Symptoms	Limitations	Need for Additional Rest*	Physical Ability To Work**
I	None	None	None	Full Time
II	Only moderate	Slight	Usually only slight or occasional	Usually full time
III	Defined, with less than ordinary activity	Marked	Usually moderate	Usually part time
IV	May be present even at rest, & any activity increases discomfort	Extreme	Marked	Unable to work

* To control or relieve symptoms, as determined by the patient, rather than as advised by the physician.

** At accustomed occupation or usual tasks.

SWOG Authorized Document

18.5 Specimen Supplement Sheet

Collection Tube(s)	Amount	Type of Sample	Size and # to ship	Time point for Collection	Collection Time Window	Where to Send	Specimen handling, storage and shipping instructions	Additional Comments
Lavender top (EDTA)	15 mL	Buffy Coat	Cryovial: 2 mL, ~1 vial	BSLN	BSLN: After consent, <= 14 days prior to tx M7/Off Tx: +/- 14 days	Lab 201, Columbus, OH	Processed ≤2 hrs post venipuncture; Centrifuge at 400 x g for 10 min; Store in freezer -70°C to -80°C; Ship on dry ice overnight delivery;	
		Plasma	Cryovial: 2 mL each, ~4 vials	BSLN, M7, Off Tx		Lab 201, Columbus, OH	Processed ≤2 hrs post venipuncture; Centrifuge at 900 x g for 10 min; Store in freezer -70°C to -80°C; Ship on dry ice overnight delivery;	
---	1	Tissue Block		BSLN	BSLN: After consent, <= 14 days prior to tx	Lab 201, Columbus, OH		**Preferred** *from radical prostatectomy or biopsy
---	30 slides	Unstained slides	30 glass slides, each containing a section of 5 micron thickness	BSLN		Lab 201, Columbus, OH	From FFPE tissue block with highest % of tumor tissue representative of Gleason score, 30 sections of 5 micron thickness mounted onto glass slides (no coverslips)	**Alternative for tissue block** *30 slides from radical prostatectomy or 10-15 slides from biopsy
red or red/black marble top (SST)	6 mL	Serum	Cryovial: 1 mL each, ~2 vials	BSLN, M7, M13 or Off Tx	BSLN: After consent, <= 14 days prior to tx M13/Off Tx: +/- 14 days	Lab 208, Salt Lake City, UT	Allow blood to clot for 30-60 min; Centrifuge at 900 x g for 10-15 min; Store in freezer -20°C; Ship on dry ice overnight delivery;	3rd specimen submission = M13 or progression (whichever occurs first) *Serum collection is NOT required for patients registered on or after 9/23/2014. Patients registered prior to 9/23/2014 are required to submit serum for all 3 designated timepoints.
1 CellSave, 3 EDTA	35 mL	Whole Blood	CellSave: 8-10 mL, ~1 tube EDTA: 8-10 mL each, ~2 tubes LiquidBiopsy: ~1 tube (fixative plus 8-10 mL blood transferred from 1 EDTA tube)	BSLN, Off Tx	BSLN: After consent, <= 14 days prior to tx Off Tx: +/- 14 days	Lab 181, Los Angeles, CA	Store at room temp (do not freeze) Ship **on day of collection** by overnight delivery. Ship Mon-Thurs ONLY.	**Kits provided.** Contact Lab 181 to order kits (*see protocol 15.2.c*)

SWOG Authorized Document
