# Supplementary material for: Race and Treatment Outcomes in Patients With Metastatic Castration-Sensitive Prostate Cancer: A Secondary Analysis of the SWOG 1216 Phase 3 Trial
Source: JAMA Netw Open. 2023 Aug 1;6(8):e2326546. doi: 10.1001/jamanetworkopen.2023.26546 (PMC10394570; doi:10.1001/jamanetworkopen.2023.26546)
Supplement: Supplement 2. — eFigure 1. CONSORT Diagram for the S1216 Trial eFigure 2. Kaplan-Meier Estimates of Overall Survival and Progression-Free Survival by Race [file jamanetwopen-e2326546-s002.pdf]

Supplemental Online Content

Sayegh N, Swami U, Jo Y, et al. Race and treatment outcomes in patients with metastatic castration-sensitive prostate cancer: a secondary analysis of the SWOG 1216 phase 3 trial. *JAMA Netw Open.* 2023;6(8):e2326546.
doi:10.1001/jamanetworkopen.2023.26546

eFigure 1. CONSORT Diagram for the S1216 Trial

eFigure 2. Kaplan-Meier Estimates of Overall Survival and Progression-Free Survival by Race

This supplemental material has been provided by the authors to give readers additional information about their work.

© 2023 Sayegh N et al. *JAMA Network Open.*

Supplementary Figure 1. CONSORT diagram for the S1216 trial.

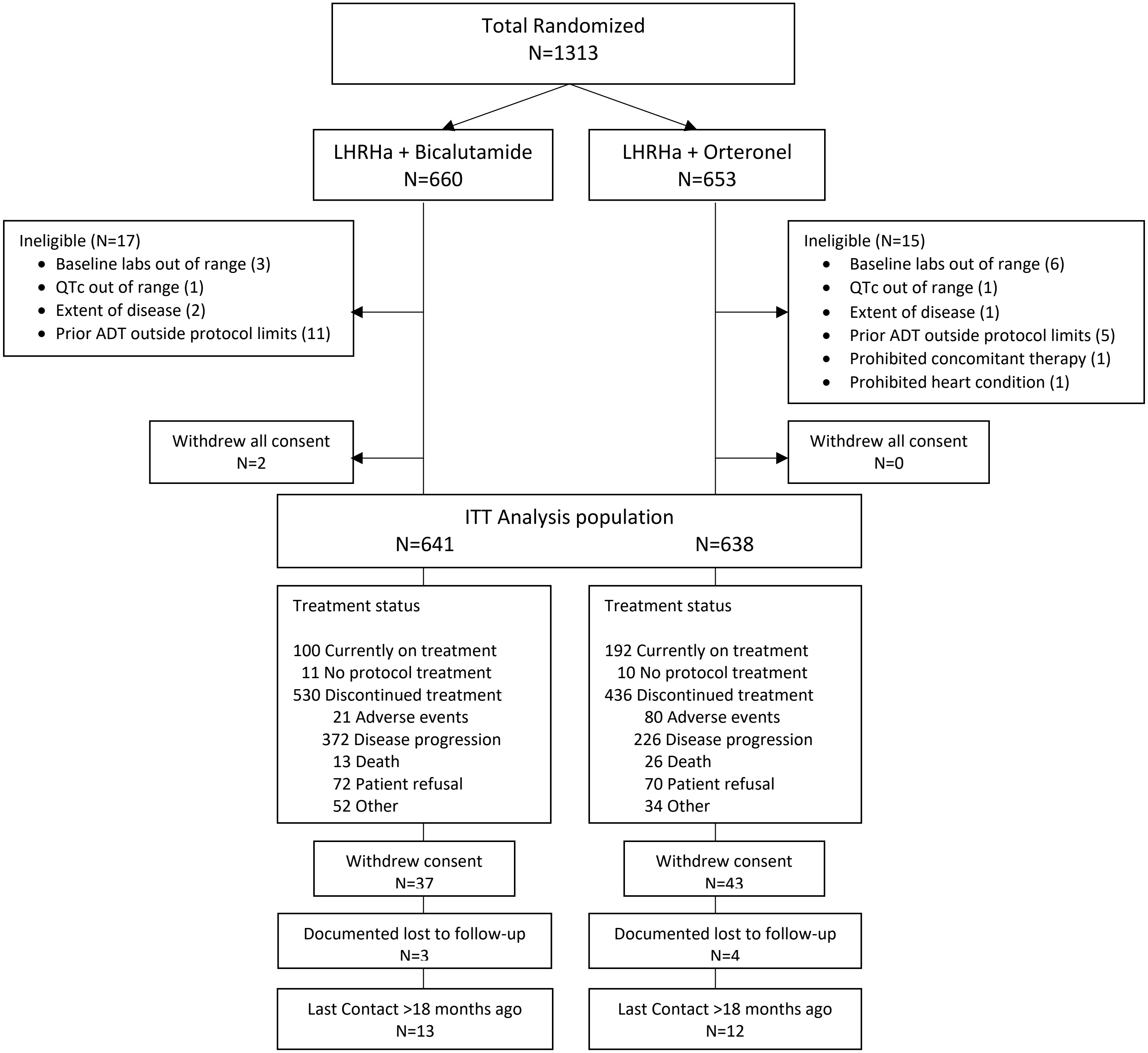

© 2023 Sayegh N et al. *JAMA Network Open.*

eFigure 2. Kaplan-Meier estimates of overall survival (OS; in the treatment arm [**A**], and the control arm [**B**]) and progression-free survival (PFS; in the treatment arm [**C**], and the control arm [**D**]) by race.

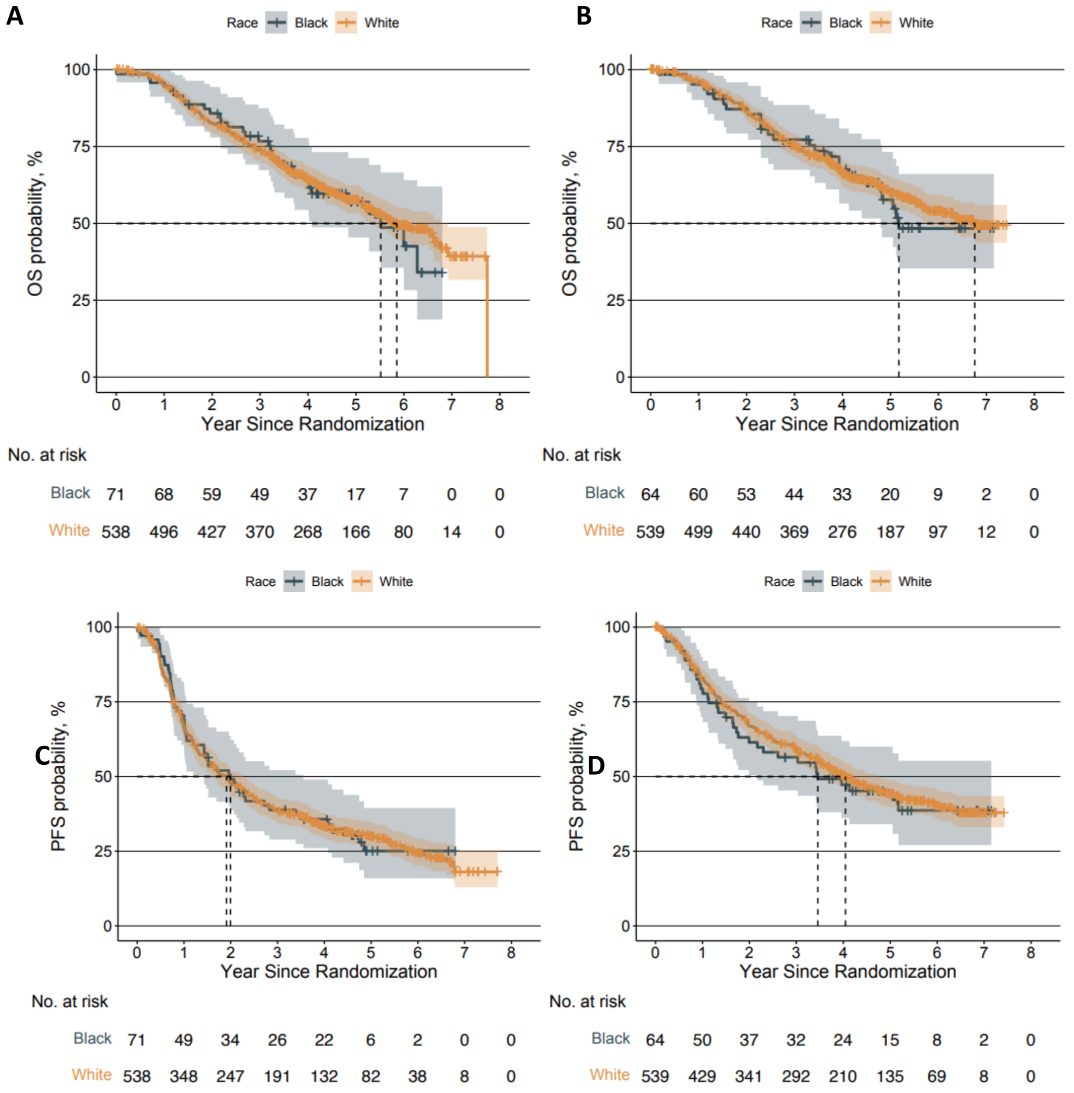

© 2023 Sayegh N et al. *JAMA Network Open.*
