# Supplementary material for: Race and Treatment Outcomes in Patients With Metastatic Castration-Sensitive Prostate Cancer: A Secondary Analysis of the SWOG 1216 Phase 3 Trial
Source: JAMA Netw Open. 2023 Aug 1;6(8):e2326546. doi: 10.1001/jamanetworkopen.2023.26546 (PMC10394570; doi:10.1001/jamanetworkopen.2023.26546)
Supplement: Supplement 3. — Data Sharing Statement [file jamanetwopen-e2326546-s003.pdf]

Data Sharing Statement

Sayegh. Race and Treatment Outcomes in Patients With Metastatic Castration-Sensitive Prostate Cancer. *JAMA Netw Open*. Published August 01, 2023. doi:10.1001/jamanetworkopen.2023.26546

Data
Data available: No

Additional Information
Explanation for why data not available: This is a NCI funded SWOG trial. The data will appear in the public domain in the due course of time after the analysis of all pre-specified endpoints have been reported. However, we do not know exactly when this will happen.
